# Integrating buccal and occlusal dental microwear with isotope analyses for a complete paleodietary reconstruction of Holocene populations from Hungary

**DOI:** 10.1038/s41598-021-86369-x

**Published:** 2021-03-29

**Authors:** Raquel Hernando, Beatriz Gamarra, Ashley McCall, Olivia Cheronet, Daniel Fernandes, Kendra Sirak, Ryan Schmidt, Marina Lozano, Tamás Szeniczey, Tamás Hajdu, Annamária Bárány, András Kalli, Eszter K. Tutkovics, Kitti Köhler, Krisztián Kiss, Judit Koós, Piroska Csengeri, Ágnes Király, Antónia Horváth, Melinda L. Hajdu, Krisztián Tóth, Róbert Patay, Robin N. M. Feeney, Ron Pinhasi

**Affiliations:** 1 Departament d’Història i Història de l’Art, Universitat Rovira i Virgili, Avinguda de Catalunya 35, 43002 Tarragona, Spain; 2 Institut Català de Paleoecologia Humana i Evolució Social (IPHES-CERCA), Zona Educacional 4, Campus Sescelades URV (Edifici W3), 43007 Tarragona, Spain; 3 School of Archaeology and Earth Institute, University College Dublin, Belfield, Dublin 4, Ireland; 4 Department of Evolutionary Anthropology, University of Vienna, AltrantraBe 14, Vienna, Austria; 5 CIAS, Department of Life Sciences, University of Coimbra, 3000-456 Coimbra, Portugal; 6 Department of Genetics, Harvard Medical School, Boston, MA 02115 USA; 7 Department of Human Evolutionary Biology, Harvard University, Cambridge, MA 02138 USA; 8 CIBIO-InBIO, Centro de Investigação em Biodiversidade e Recursos Genéticos, Universidade do Porto, Rua Padre Armando Quintas 7, 4485-661 Vairao, Porto, Portugal; 9 Department of Biological Anthropology, Eötvös Loránd University, Pázmány Péter sétány 1/c, Budapest, 1117 Hungary; 10 Department of Anthropology, Hungarian Natural History Museum, Ludovika tér 2, Budapest, 1083 Hungary; 11 Department of Archaeology, Hungarian National Museum, Múzeum krt. 14-16, Budapest, 1088 Hungary; 12 Várkapitányság Integrált Területfejlesztési Központ Nonprofit Zrt., Daróczi Út 3., Budapest, 1113 Hungary; 13 Rétközi Museum, Csillag u. 5., Kisvárda, 4600 Hungary; 14 Institute of Archaeology, Research Centre for the Humanities, Loránd Eötvös Research Network, Tóth Kálmán utca 4, Budapest, 1097 Hungary; 15 Herman Ottó Museum, Görgey Artúr u. 28, Miskolc, 3529 Hungary; 16 Dornyay Béla Museum, Múzeum tér 2., Salgótarján, 3100 Hungary; 17 Department of Archaeology, Ferenczy Museum Center, Fő tér 2–5, Szentendre, 2000 Hungary; 18 School of Medicine, University College Dublin, Belfield, Dublin 4, Ireland

**Keywords:** Anthropology, Archaeology

## Abstract

Dietary reconstruction is used to make inferences about the subsistence strategies of ancient human populations, but it may also serve as a proxy to characterise their diverse cultural and technological manifestations. Dental microwear and stable isotope analyses have been shown to be successful techniques for paleodietary reconstruction of ancient populations but, despite yielding complementary dietary information, these techniques have rarely been combined within the same study. Here we present for the first time a comprehensive approach to interpreting ancient lifeways through the results of buccal and occlusal microwear, and δ^13^C and δ^15^N isotope analyses applied to the same individuals of prehistoric populations of Hungary from the Middle Neolithic to the Late Bronze Age periods. This study aimed to (a) assess if the combination of techniques yields a more precise assessment of past dietary and subsistence practices, and (b) contribute to our understanding of the dietary patterns of the prehistoric Hungarian populations. Overall, no correlations between microwear and δ^13^C and δ^15^N isotope variables were observed, except for a relationship between nitrogen and the vertical and horizontal index. However, we found that diachronic differences are influenced by the variation within the period. Particularly, we found differences in microwear and isotope variables between Middle Neolithic sites, indicating that there were different dietary practices among those populations. Additionally, microwear results suggest no changes in the abrasiveness of the diet, neither food processing methods, despite higher C_4_ plant resource consumption shown by carbon isotopic signal. Thus, we demonstrate that the integration of dental microwear and carbon and nitrogen stable isotope methodologies can provide complementary information for making inferences about paleodietary habits.

## Introduction

The transitions between the Neolithic, Copper, and Bronze Ages constitute key periods of important social changes in the configuration of European societies that included the creation of hierarchies and the establishment of agriculture^1–3^. The adoption of an agriculture and/or a pastoral lifestyle is one of the most important events in human history, resulting in significant biological, environmental, cultural and health changes^3–6^. By reconstructing the diet of these transitional populations, we can better understand the changes these societies experienced at different levels, including subsistence strategies, landscape use, health, and/or social stratification^7–12^.

In the case of the Great Hungarian Plain (GHP), a series of changes in settlement patterns and subsistence strategies occurred from the Neolithic through the Copper Age and into the Bronze Age^13,14^ (Table 1). During the transition from the Neolithic to the Copper Age, people dispersed from large settlements to smaller nuclear villages^13,15^. There was an increased focus on animal husbandry and less reliance on agricultural products, which included an increase in the use of secondary products such as milk and wool^16^. This shift was also accompanied by a diversification of material culture^17,18^. Afterwards, over the course of the Bronze Age, the intensification of agriculture and building of extensive trade networks took place due to the arrival of bronze metallurgy from the northern Pontic and Balkan Peninsulas^19,20^. In addition, there was an increase in millet cultivation in the Middle and Late Bronze Age^21,22^. During the last phase of the Bronze Age, great economic changes occurred, associated with cultural connections to Central Europe^23–25^.

**Table 1:** Summary of the prehistoric time periods and their and subsistence practices in the Great Hungarian Plain.

Time period	Time range (BC)	Subsistence strategies
Middle Neolithic	5500–5000	Grain cultivation (wheat, barley, einkorn) and animal husbandry (major reliance on cattle)
Late Neolithic	5000–4500	Grain cultivation (wheat, barley, einkorn); animal husbandry with emphasis on domesticated cattle)
Early Copper Age	4500–4000	Focus on animal husbandry (mainly cattle)
Middle Copper Age	4000–3500	Focus on animal husbandry (mainly cattle)
Late Copper Age	3500–2800	Focus on animal husbandry (mainly cattle)
Transitional Period^a^	2800–2600	Horse domestication (?), nomadic (mobile) way of life and livestock (cattle and sheep ?) husbandry
Early Bronze Age	2600/2550–2000/1900	Intensive crop cultivation and animal husbandry
Middle Bronze Age	2000/1900–1450/1400	Intensive crop cultivation and animal husbandry; millet consumption
Late Bronze Age	1450/1400–800	Intensive crop cultivation; millet as stable crop

^a^Transition to the Bronze Age after the constant East European (Yamnaya) impact between 3100/3000 and 2800 BC (Refs.^26–32^).

Dietary patterns and subsistence practices of prehistoric populations have long been inferred from cultural and archaeological remains^33–35^, but these studies are now most often being complemented by a variety of bioarchaeological and molecular approaches^7,8,36–41^. Dental microwear and stable isotope analyses have been largely used to infer dietary practices in Holocene populations^36,37,42–47^. Dental microwear studies on buccal and occlusal surfaces of teeth provide insight into the physical properties of the food ingested by humans and other animals by yielding information about the abrasiveness of the food coupled with how these foods were processed prior to consumption^48–53^. During chewing, pits of different sizes and striations of different lengths and orientations are formed across enamel surfaces caused by particles harder than enamel, such as plant phytoliths, grit, or quartz dust ^43,50,54–57^. As such, distinct dietary habits and food processing methods result in different microwear patterns, allowing for the distinction, for example, between individuals who ingested foodstuffs obtained through different subsistence practices, such as foraging and agriculture, or between farming and pastoralism^36,37,58,59^.

Carbon and nitrogen stable isotope analyses, in turn, are based on the principle that the biochemical composition of the food consumed by animals and humans is preserved in their body tissues^60–62^. The isotopic signature in archaeological human tissues differs from resources consumed and consumers in a predictable isotopic fractionation, with a generally accepted stepwise increase of 0–2‰ for carbon^63^ and 3–5‰ for nitrogen isotopic signatures^64–66^. Carbon (δ^13^C) and nitrogen (δ^15^N) isotope ratios measured from bone collagen of archaeological samples are indicative of the protein consumed by the individual about 10–15 years prior to death^67^. In brief, δ^13^C is primarily used to identify the consumption of plants that use different metabolic pathways for fixing carbon during photosynthesis and the animals raised on them. These δ^13^C differences can then be used to identify the consumption of C_3_ versus C_4_ terrestrial resources in past diets^68–70^. Values of carbon ratios may also inform about the consumption of marine foods^71–74^ and freshwater resources^75–77^.

In addition to carbon isotope signals, values of δ^15^N in bone collagen are primarily used to analyse the relative presence of plant versus animal protein in the diet^63,78^. It is based on the observed increase of δ^15^N in tissues with the increase of trophic level^63,79^. Nitrogen isotope signals are also used to distinguish between the consumption of terrestrial foods and marine and freshwater resources^64,75^. Other environmental factors can also influence δ^15^N in tissues, such as the consumption of fertilized crops^80,81^, the health of the individual^82^, nursing practices^83^, and/or other environmental factors^84–86^.

Both approaches offer different insights into dietary reconstruction, and although they are complementary, their combined use for paleodietary reconstruction is rare^46,87–90^. Still, some research has been done in this line^46,89,90^, showing interesting results of the combination of buccal dental microwear and stable isotopes and enhancing the importance of integrating both methods. Within dental microwear, analyses combining buccal and occlusal surfaces are also becoming more popular^91,92^ due to differences in indentation enamel processes, which can provide dietary information over both short and long time scales^48,54,55,93–96^. The central aim of this study is to assess whether the combined use of dental microwear and isotope data provide a better understanding of the dietary patterns and the types of food consumed by past Hungarian populations. Remarkably, this study takes a step further, not only by including information from the occlusal surface too, but also and unlike previous studies^88,89^, by employing the same individuals for all the data obtained. The integration of the data obtained from these three proxies contributes for a dietary approximation with a more fine-grained approach.

Here, we contribute to a better understanding of the dietary practices of people in the GHP from the Middle Neolithic to the Late Bronze Age by studying their diets at different levels: (a) at the individual level, by comparing both techniques in the same individual; (b) at the diachronic level, by observing potential differences between chronologies; and (c) at the regional level, by comparing contemporaneous sites.

## Materials

**Figure 1:**
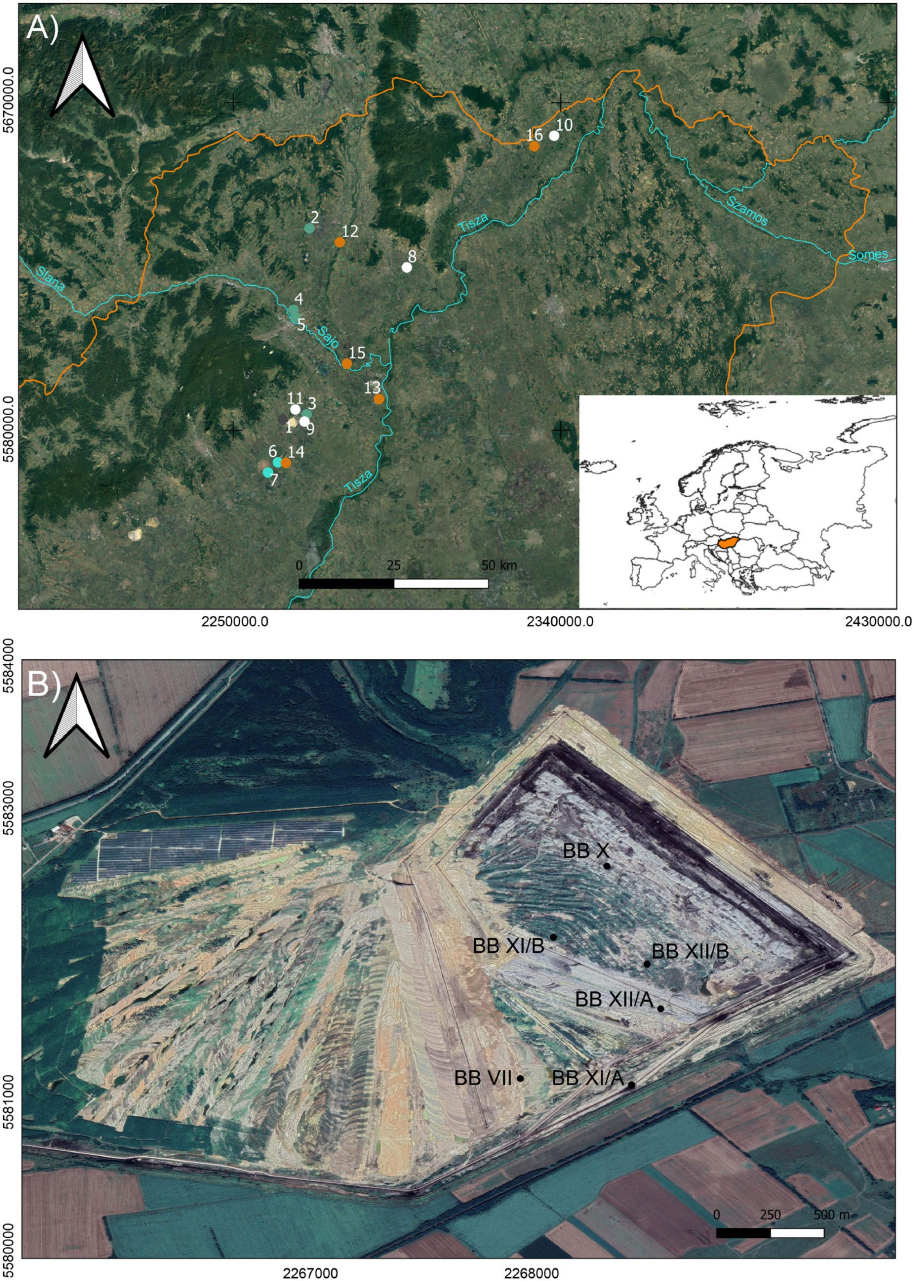
Hungarian map showing the location of sites analysed in the study. QGis free software (version 3.10), (https://www.qgis.org/es/site/) was used to create this map, with the coordinate system ETR89/UTM Zone 30 N. (**A**) Scale 1:1.200,000; archaeological sites represented: Middle Neolithic (1. Bükkábrány-Bánya; 2. Rásonysápberencs-Szőlő alja; 3. Csincse-Gomba Barna földje (M3- site 14–16); 4. Arnót-Nagy-bugyik; 5. Arnót-Arnóti-oldal Dél); Middle Copper Age (6. Mezőkövesd-Klementina (Szentistván-Reptér); 7. Mezőkövesd-Patakra járó dűlő); Middle Bronze Age (8. Mezőzombor-Községi temető; 9. Mezőkeresztes-Csincsetanya; 10. Nagyrozvágy-Papdomb; 11. Vatta-Dobogó); Late Bronze Age (12. Felsődobsza-site 2; 13. Oszlár-Nyárfaszög (M3-site 32); 14. Mezőkeresztes-Cet halom (M3-site 10); 15. Köröm-Kápolna-domb; 16. Pácin-Alsókenderszer). (**B**) Scale 1:10,000; Bükkábrány-Bánya lignite mine area with archaeological sites (BB) represented.

Archaeological human skeletal samples of both sexes from a wide range of ages at death, spanning from the Middle Neolithic to the Late Bronze Age, along with faunal material, were sampled from 17 sites across the GHP (Fig. 1; see details in “Supplementary Information Archaeological sites descriptions” and Supplementary Table S9). Postcranial elements, petrous bones, and dental remains were collected for each individual, when possible, from the osteological collection of the Herman Ottó Museum (Miskolc, Hungary). Sex and age-at-death were estimated based on established bioanthropological methods (described in “Supplementary Information Methods”). Individuals were also assigned sex using ancient DNA (aDNA) methods. In the case of divergence, aDNA sex results were favoured. For those individuals whose sex could not be identified using aDNA, osteological information was used (see more details in “Supplementary Information Methods”).

As in other dental microwear studies^92,97,98^, there was a great amount of teeth in which dental microwear could not be observed for different reasons (e.g. enamel preservation). This fact made that small sample size be a typical unavoidable reality. From a total number of 65 individuals, buccal and occlusal well preserved microwear were obtained in 49 human posterior teeth from different individuals of both sexes and various ages. Teeth present different post-depositional modification, from no detectable to severe modification. Although, experimental studies have shown that *post-mortem* damage could be discerned from *ante-mortem* microwear^99,100^, teeth with severe evidence of *post-mortem* damage were excluded from the analysis. In the case of occlusal surface, enamel wear related with abrasive diets or the effect of age, affect directly to this surface and obscure dental microwear as wear progresses^93^. Lower molars were preferred as sample size was higher. Nevertheless, when dental microwear was not possible to be observed in the lower molars, upper molars from the same individual were chosen instead. Carbon and nitrogen stable isotope analysis was carried out on collagen from bone samples of 89 human individuals from both sexes and various ages, along with 29 faunal bone samples (Table 2).

**Table 2:** List of individuals employed in this study.

ID	Grave number	Site	Period	Culture	Age (years)	Age range category	DNA sex	Osteo Sex	Isotope analyses	Microwear analyses
HUNG870	S308	Bükkábrány-Bánya VII	MN	ALBK 1st phase	30–34	Adult	Failed	?	S,P	
HUNG871	S249	Bükkábrány-Bánya VII	MN	ALBK 1st phase	7–8	Infant II	–	–		√
HUNG872	S241	Bükkábrány-Bánya VII	MN	ALBK 1st phase	15–20	Juvenile	**M**	?	√	√
HUNG873	250	Bükkábrány-Bánya VII	MN	ALBK 1st phase	15–25	Juvenile-**Adult**	Failed	?	S,P	√
HUNG874	S478	Bükkábrány-Bánya VII	MN	ALBK 1st phase	8–9	Infant II	M	–	√	
HUNG876	S287	Bükkábrány-Bánya VII	MN	ALBK 1st phase	15–20	**Juvenile**-Adult	–	–	S,P	
HUNG877	S254	Bükkábrány-Bánya VII	MN	ALBK 1st phase	20–30	Adult	Failed	?	S,P	√
HUNG878	S248	Bükkábrány-Bánya VII	MN	ALBK 1st phase	20–40	Adult	Failed	?	S,P	√
HUNG879	S480	Bükkábrány-Bánya VII	MN	ALBK 1st phase	3–5	Infant I	Failed	–		
HUNG880	S25	Bükkábrány-Bánya X	MN	ALBK	Adult	Adult	Failed	?	P	√
HUNG882	S11	Bükkábrány-Bánya XI/A	MN	ALBK 1st phase	1–6	Infant I	U	–		
HUNG883	S318	Bükkábrány-Bánya XI/A	MN	ALBK 1st phase	30–50	Adult-Mature	Failed	**M**	√	
HUNG884	S101	Bükkábrány-Bánya XI/A	MN	ALBK 1st phase	30–40	Adult	**M**	?	√	√
HUNG885	S379	Bükkábrány-Bánya XI/A	MN	ALBK 1st phase	35–45	Adult–Mature	**F**	?	√	√
HUNG887	S414	Bükkábrány-Bánya XI/A	MN	ALBK 1st phase	35–45	Adult	**M**	F	√	
HUNG888	S52	Bükkábrány-Bánya XI/A	MN	ALBK 1st phase	11–13	Infant II	Failed	–	S,P	√
HUNG889	S435	Bükkábrány-Bánya XI/A	MN	ALBK 1st phase	20–30	Adult	**M**	?	√	√
HUNG890	S102	Bükkábrány-Bánya XI/A	MN	ALBK 1st phase	8–9	Infant II	F	–	√	OM
HUNG891	S311	Bükkábrány-Bánya XI/A	MN	ALBK 1st phase	1–6	Infant I	F	–		
HUNG892	S413	Bükkábrány-Bánya XI/A	MN	ALBK 1st phase	1–3	Infant I	F	–		
HUNG893	S217	Bükkábrány-Bánya XI/A	MN	ALBK 1st phase	11–12	Infant II	M	–	√	√
HUNG894	S273	Bükkábrány-Bánya XI/A	MN	ALBK 1st phase	25–30	Adult	F	F	√	√
HUNG895	S272	Bükkábrány-Bánya XI/A	MN	ALBK 1st phase	Adult	Adult	**M**	?	√	√
HUNG913	S1171	Bükkábrány-Bánya XII/A	MN	ALBK	9–12	Infant II	Failed	–	P	√
HUNG920	S308	Bükkábrány-Bánya XII/B	MN	ALBK	Adult	Adult	U	?	S,P	√
HUNG921	S164	Bükkábrány-Bánya XII/B	MN	ALBK	Adult	Adult	M	?	√	
HUNG922	S273	Bükkábrány-Bánya XII/B	MN	ALBK	Adult	Adult	Failed	?	S,P	BM
HUNG923	S277	Bükkábrány-Bánya XII/B	MN	ALBK	20–30	Adult	–	?	S,P	
HUNG924	S300	Bükkábrány-Bánya XII/B	MN	ALBK	Adult	Adult	Failed	**F**	√	√
HUNG926	S320	Bükkábrány-Bánya XII/B	MN	ALBK	Adult	Adult	–	M	√	
HUNG927	S299	Bükkábrány-Bánya XII/B	MN	ALBK	Adult	Adult	Failed	?		
HUNG928	S33	Bükkábrány-Bánya XII/B	MN	ALBK	1–6	Infant I	F	–		
HUNG929	S306	Bükkábrány-Bánya XII/B	MN	ALBK	Adult	Adult	Failed	?	S,P	
HUNG930	S276	Bükkábrány-Bánya XII/B	MN	ALBK	Adult	Adult	Failed	?	S,P	
HUNG931	S112	Bükkábrány-Bánya XII/B	MN	ALBK	1–6	Infant I	F	–		
HUNG932	S278	Bükkábrány-Bánya XII/B	MN	ALBK	12–15	**Infant II**-Juvenile	Failed	–	S,P	OM
HUNG941	S237	Rásonysápberencs-Szőlő alja	MN	Tiszadob-Bükk	Adult	Adult	**M**	?	√	
HUNG942	S379	Rásonysápberencs-Szőlő alja	MN	Tiszadob-Bükk	Adult	Adult	**F**	?	√	
HUNG943	S215	Rásonysápberencs-Szőlő alja	MN	Tiszadob-Bükk	9–12	Infant II	F	–	√	
HUNG948	4	Csincse-Gomba Barna földje (M3–site14-16	MN	ALBK Late phase/Early Tiszadob	Adult	Adult	Failed	**F**		
HUNG953	S18	Arnót-Nagy-bugyik	MN	ALBK	20–35	Adult	Failed	?	P	
HUNG955	S20	Arnót-Nagy-bugyik	MN	ALBK	2–5	Infant I	M	–		
HUNG956	S127	Arnót-Arnóti-oldal Dél	MN	ALBK	30–50	Adult-Mature	F	?	P,G	
HUNG896	S725	Bükkábrány-Bánya XI/B	MCA	Bodrogkeresztúr	Adult	Adult	–	?		√
HUNG897	S1168	Bükkábrány-Bánya XI/B	MCA	Bodrogkeresztúr	Adult	Adult	M	M	√	√
HUNG898	S475	Bükkábrány-Bánya XI/B	MCA	Bodrogkeresztúr	Adult	Adult	**F**	?	√	√
HUNG899	S361	Bükkábrány-Bánya XI/B	MCA	Bodrogkeresztúr	20–40	Adult	**F**	?	√	√
HUNG900	S359	Bükkábrány-Bánya XI/B	MCA	Bodrogkeresztúr	Adult	Adult	Failed	?	S,P	
HUNG901	S484	Bükkábrány-Bánya XI/B	MCA	Bodrogkeresztúr	Adult	Adult	Failed	?	S,P	BM
HUNG902	S373	Bükkábrány-Bánya XI/B	MCA	Bodrogkeresztúr	Adult	Adult	U	?	S,P	
HUNG903	S371-1.11	Bükkábrány-Bánya XI/B	MCA	Bodrogkeresztúr	17–25	Juvenile–**Adult**	Failed	**F**	√	√
HUNG904	S371-3.11	Bükkábrány-Bánya XI/B	MCA	Bodrogkeresztúr	45–49	Mature	**M**	?	√	√
HUNG908	S349	Bükkábrány-Bánya XI/B	MCA	Bodrogkeresztúr	Adult	Adult	Failed	?	S,P	√
HUNG909	S360	Bükkábrány-Bánya XI/B	MCA	Bodrogkeresztúr	Adult	Adult	Failed	?	S,P	√
HUNG910	S288	Bükkábrány-Bánya XI/B	MCA	Bodrogkeresztúr	Adult	Adult	Failed	?	S,P	√
HUNG911	S289	Bükkábrány-Bánya XI/B	MCA	Bodrogkeresztúr	5–7	**Infant I**–Infant II	Failed	–		√
HUNG912	S472	Bükkábrány-Bánya XI/B	MCA	Bodrogkeresztúr	Adult	Adult	–	?		**√**
HUNG939	1	Mezőkövesd-Klementina (Szentistván-Reptér)	MCA	Bodrogkeresztúr	12–18	Infant II–**Juvenile**	F	–	P,G	
HUNG940	3	Mezőkövesd-Klementina (Szentistván-Reptér)	MCA	Bodrogkeresztúr	Adult	Adult	**M**	?	P,G	
HUNG961	4/A	Mezőkövesd-Patakra járó dűlő	MCA	Bodrogkeresztúr	17–21	**Juvenile**–Adult	M	M	√	
HUNG962	4/A_1st child	Mezőkövesd-Patakra járó dűlő	MCA	Bodrogkeresztúr	5–6	Infant I	M	–		
HUNG963	4/A_2nd child	Mezőkövesd-Patakra járó dűlő	MCA	Bodrogkeresztúr	1–6	Infant I	M	–		
HUNG964	4	Mezőkövesd-Patakra járó dűlő	MCA	Bodrogkeresztúr	10–12	Infant II	Failed	–	S,P	
HUNG965	17	Mezőkövesd-Patakra járó dűlő	MCA	Bodrogkeresztúr	40–60	Mature	Failed	**M**	√	
HUNG966	sir 5	Mezőkövesd-Patakra járó dűlő	MCA	Bodrogkeresztúr	30–60	Adult–Mature	Failed	**F**	√	
HUNG914	S1000	Bükkábrány-Bánya XII/A	LCA	Baden	9–12	Infant II	–	–		√
HUNG915	S2044	Bükkábrány-Bánya XII/A	LCA	Baden	15–20	**Juvenile**–Adult	F	F	P,G	√
HUNG917	S1001	Bükkábrány-Bánya XII/A	LCA	Baden	4–7	**Infant I** – Infant II	M	–		OM
HUNG918	S65	Bükkábrány-Bánya XII/B	LCA	Baden	39–50	Adult—Mature	**F**	M	P,G	
HUNG919	S67	Bükkábrány-Bánya XII/B	LCA	Baden	35–45	Adult–Mature	**F**	M	P,G	√
HUNG127	4/2000	Mezőzombor-Községi temető	MBA	Füzesabony	20–39	Adult	F	F	√	OM
HUNG128	3/2000	Mezőzombor-Községi temető	MBA	Füzesabony	10–13	Infant II	M	–	√	
HUNG129	2/2000	Mezőzombor-Községi temető	MBA	Füzesabony	35–44	Adult–Mature	–	M	√	
HUNG130	16/2000	Mezőzombor-Községi temető	MBA	Füzesabony	20–39	Adult	F	**F**	√	BM
HUNG131	41/2001	Mezőzombor-Községi temető	MBA	Füzesabony	34–42	Adult–Mature	–	M	√	
HUNG132	6/2000	Mezőzombor-Községi temető	MBA	Füzesabony	5–7	**Infant I**–Infant II	F	–		√
HUNG133	31/2001	Mezőzombor-Községi temető	MBA	Füzesabony	20–39	Adult	–	M	√	
HUNG134	10/2001	Mezőzombor-Községi temető	MBA	Füzesabony	5–10	Infant I–**Infant II**	M	–		√
HUNG135	56/2001	Mezőzombor-Községi temető	MBA	Füzesabony	35–50	Adult-Mature	–	M	√	
HUNG136	57/2001	Mezőzombor-Községi temető	MBA	Füzesabony	33–46	Adult–Mature	F	F	√	
HUNG147	1	Mezőkeresztes-Csincse-tanya	MBA	Füzesabony	12–14	Infant II	–	–	P	√
HUNG163	S296	Nagyrozvágy-Papdomb	MBA	Füzesabony	35–45	Adult–Mature	–	M	P,G	BM
HUNG933	S109	Vatta-Dobogó	MBA	Füzesabony	8–13	Infant II	F	–	√	√
HUNG934	S257/II	Vatta-Dobogó	MBA	Füzesabony	20–39	Adult	–	?	S,P	BM
HUNG935	S169	Vatta-Dobogó	MBA	Füzesabony	Adult	Adult	F	?	√	
HUNG936	S279	Vatta-Dobogó	MBA	Füzesabony	40–59	Mature	U	M	√	
HUNG937	S257/I	Vatta-Dobogó	MBA	Füzesabony	20–30	Adult	U	?		√
HUNG938	S166	Vatta-Dobogó	MBA	Füzesabony	20–39	Adult	F	?	√	
HUNG137	S62	Felsődobsza-2. lelőhely	LBA	pre-Gáva Period, R BD—Ha A1	34–42	Adult-Mature	M	M	P,G	
HUNG144	1010	Oszlár-Nyárfaszög (M3-32. lelőhely)	LBA	pre-Gáva Period, R BD—Ha A1	20–39	Adult	–	M	P,G	
HUNG177	154. objektum	Mezőkeresztes-Cethalom (M3-10. lelőhely)	LBA	pre-Gáva Period, R BD—Ha A1	6–10	Infant I–**Infant II**	U	–	P	√
HUNG863	S67	Köröm-Kápolnadomb	LBA	Gáva culture	20–39	Adult	–	F	P,G	BM
HUNG967	S64A	Pácin-Alsókenderszer	LBA	pre-Gáva Period, R BD—Ha A1	1–6	Infant I	M	–		
HUNG968	S64B	Pácin-Alsókenderszer	LBA	pre-Gáva Period, R BD—Ha A1	15–39	Juvenile–**Adult**	M	M	P,G	√
HUNG969	S100	Pácin-Alsókenderszer	LBA	pre-Gáva Period, R BD—Ha A1	30–60	Adult–Mature	M	M	P,G	

Details of the site, period, culture and biological (age, sex) information, and the statistical analyses included. Age range category: Infant I (1–6 years), Infant II (7–14 years), Juvenile (15–19 years), Adult (20–39), and Mature (40–59); in bold the age category used for statistical analyses when the sample age (years) range include two age categories^a^. Isotope data was included for period (P), site (S), gender (G) and/or all (√) statistical comparisons. Microwear analyses were performed in buccal (BM), occlusal (OM) or both (√) enamel surfaces. Period abbreviations: Middle Neolithic (MN), Late Neolithic (LN), Middle Copper Age (MCA), Late Copper Age (LCA), Middle Bronze Age (MBA), Late Bronze Age (LBA); Sex abbreviations: female (F), male (M), undetermined (U), not possible to assign (?).

^a^The age category selected was chosen according to the higher number of years of the age range estimation belonging to each category. When age cohort is between Adult and Mature category, no choice was made as both categories were included together as adult in the statistical tests.

## Results

We obtained buccal data from 38 molars (from a total number of 65 molars) from different adult individuals, while 34 molars from different adult individuals were obtained from occlusal microwear (Table 2 and Supplementary Tables S1 and S2). Bone collagen was successfully obtained for all human samples, and for 27 of 29 faunal samples analysed. Three human samples did not meet the acceptable atomic C:N quality range of 2.9–3.6^101^ and their isotopic data were excluded from further analyses (Table 2 and Supplementary Tables S9 and S10).

### Comparison of dental microwear and stable isotope analyses

Within individuals, results from buccal and occlusal microwear analysis were compared to stable isotope data. Spearman correlation results indicated that the majority of microwear variables and stable isotope ratios were not statistically correlated (Table 3, Supplementary Figs. S1 and S2). An exception was δ^15^N, which was correlated with the vertical (NV/BTN) and horizontal index (NH/BTN) in buccal microwear.

**Table 3:** Normality test and Spearman correlations between microwear variables and isotope ratios.

Microwear variables	Normality test			δ^13^C		δ^15^N	
	N	Shapiro–Wilks	p-value	Rho Spearman	p-value	Rho Spearman	p-value
δ^13^C	40	0.746	**< 0.01**	–	–	–	–
δ^15^N	40	0.885	**< 0.01**	–	–	–	–
BTN	40	0.972	0.430	0.051	0.752	− 0.138	0.392
XT	40	0.927	**0.013**	0.291	0.068	− 0.008	0.959
NV/BTN	40	0.969	0.344	− 0.189	0.241	0.347	**0.028**
NH/BTN	40	0.920	**< 0.01**	0.219	0.174	− 0.323	**0.041**
δ^13^C	37	0.649	**< 0.01**	–	–	–	–
δ^15^N	37	0.895	**< 0.01**	–	–	–	–
OTN	35	0.951	0.124	− 0.094	0.590	− 0.153	0.379
Pits	36	0.964	0.290	− 0.089	0.602	− 0.058	0.736
% Pits	35	0.956	0.198	0.162	0.366	0.099	0.581
Area pits	33	0.905	**< 0.01**	0.086	0.622	0.236	0.170

*N* total number of samples included, *BTN* number of striations on the buccal surface, *XT* length of striations, *NV/BTN* vertical index, *NH/BTN* horizontal index, *OTN* total number of striations in the occlusal surface, *%Pits* percentage of pits.

Significant correlations (p < 0.05) in bold.

### Dental microwear analysis

**Figure 2:**
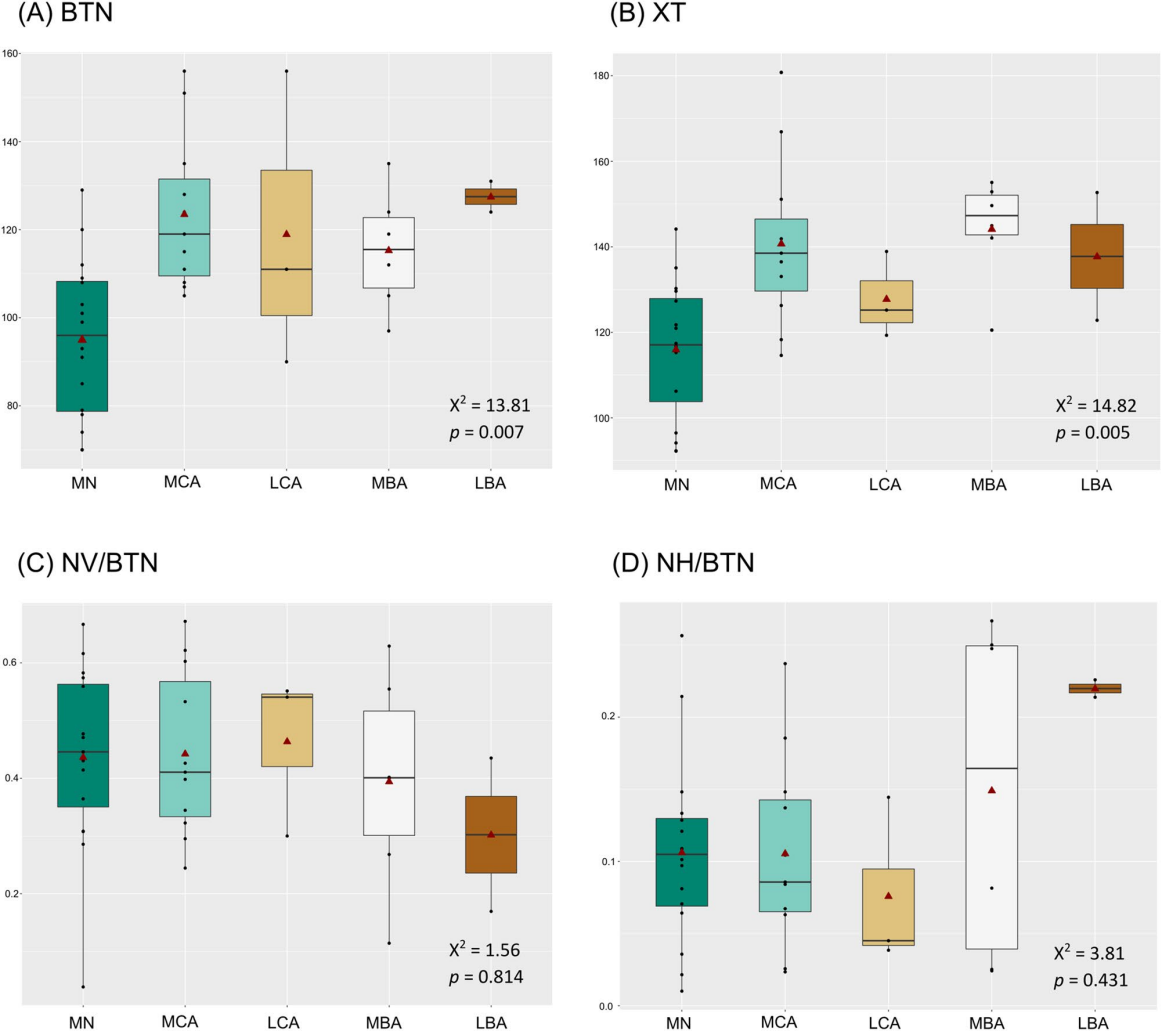
Boxplot showing (**A**) the total number and (**B**) length of buccal striations, (**C**) the vertical and (**D**) horizontal indexes by period. Samples only include individuals ≥ 8 years old (N = 38; see text for explanation). Red triangles show the means, middle horizontal lines represent the medians. Middle Neolithic (MN, n = 16); Middle Copper Age (MCA, n = 11); Late Copper Age (LCA, n = 3); Middle Bronze Age (MBA, n = 6); Late Bronze Age (LBA, n = 2).

Descriptive statistics for the buccal surface variables are summarized in Table 4 and detailed in Supplementary Table S1. Total sample consist on 65 individuals from various ages. From this sample, dental microwear was observed on 45 individuals (38 adults and 7 infants). When comparing samples between periods (N = 38), there were differences between the number of striations (BTN; Kruskal–Wallis: X^2^ = 13.81, *p* = 0.007) and their length (XT; Kruskal–Wallis: X^2^ = 14.82, *p* = 0.005) (Fig. 2, Supplementary Table S3).

**Table 4:** Summary of microwear results of human individuals included in this study.

	Groups	N	Buccal surface				N	Occlusal surface			
			BTN	XT	NV/BTN	NH/BTN		OTN	Pits	Area Pits	%Pits
By period	Middle Neolithic	16	95	116.03	0.44	0.11	16	67	19	32.73	22.47
	Middle Copper Age	11	124	140.77	0.44	0.11	10	66	13	34.48	16.62
	Late Copper Age	3	119	127.81	0.46	0.08	3	51	11	54.6	17.74
	Middle Bronze Age	6	115	144.18	0.39	0.15	4	45	17	38.56	27.37
	Late Bronze Age	2	128	137.76	0.3	0.22	1	81	23	25.33	22.12
By site	Middle Neolithic										
	Bükkábrány-Bánya VII	4	112	121.34	0.47	0.11	4	83	20	35.67	19.05
	Bükkábrány-Bánya X	1	120	129.62	0.31	0.13	1	44	21	24.96	32.31
	Bükkábrány-Bánya XI/A	7	87	115.26	0.51	0.06	7	62	15	35.55	20.66
	Bükkábrány-Bánya XII/A	1	103	127.33	0.58	0.10	1	68	26	25.59	27.66
	Bükkábrány-Bánya XII/B	3	80	102.46	0.21	0.2	3	63	21	27.73	28.16
	Middle Copper Age										
	Bükkábrány-Bánya XI/B	11	124	140.77	0.44	0.11	10	66	13	34.48	16.62
	Late Copper Age										
	Bükkábrány-Bánya XII/A	2	123	122.25	0.43	0.09	2	54	14	47.43	20.13
	Bükkábrány-Bánya XII/B	1	111	138.91	0.54	0.05	1	47	7	68.93	12.96
	Middle Bronze Age										
	Mezőzombor-Községi temető	1	119	155.05	0.55	0.03	1	32	9	38.16	21.95
	Mezőkeresztes-Csincse-tanya	1	105	144.94	0.11	0.27	1	42	16	39.55	27.59
	Nagyrozvágy-Papdomb	1	124	142.06	0.63	0.02	–	–	–	–	–
	Vatta-Dobogó	3	115	141	0	0	2	60	21	38.26	32.58
	Late Bronze Age										
	Köröm-Kápolnadomb	1	124	122.82	0.17	0.23	–	–	–	–	–
	Pácin-Alsókenderszer	1	131	152.69	0.44	0.21	1	81	23	25.33	22.12
By sex	All samples										
	Female	11	117	122.76	0.4	0.11	11	65	14	37.74	18.36
	Male	9	106	135.93	0.53	0.08	7	68	17	34.54	20.51
	Middle Neolithic										
	Female	3	88	100.55	0.37	0.12	3	71	17	28.92	19.20
	Male	4	93	116.74	0.56	0.07	5	68	15	36.63	18.69
	Middle Copper Age										
	Female	3	128	128.73	0.38	0.16	4	74	11	37.74	14.15
	Male	2	117	173.83	0.48	0.05	1	54	21	33.27	28.00
	Middle Bronze Age										
	Female	2	127	137.79	0.48	0.05	2	46	19	33.70	27.27
	Male	1	124	142.06	0.63	0.02	–	–	–	–	–

*N* total number of adult individuals (see text for explanation; *number of adult individuals when comparing by period and site); *BTN* number of striations on the buccal surface; *XT* length of striations in µm; *NV/BTN* vertical index; *NH/BTN* horizontal index; *OTN* total number of striations in the occlusal surface; *%Pits* percentage of pits.

**Figure 3:**
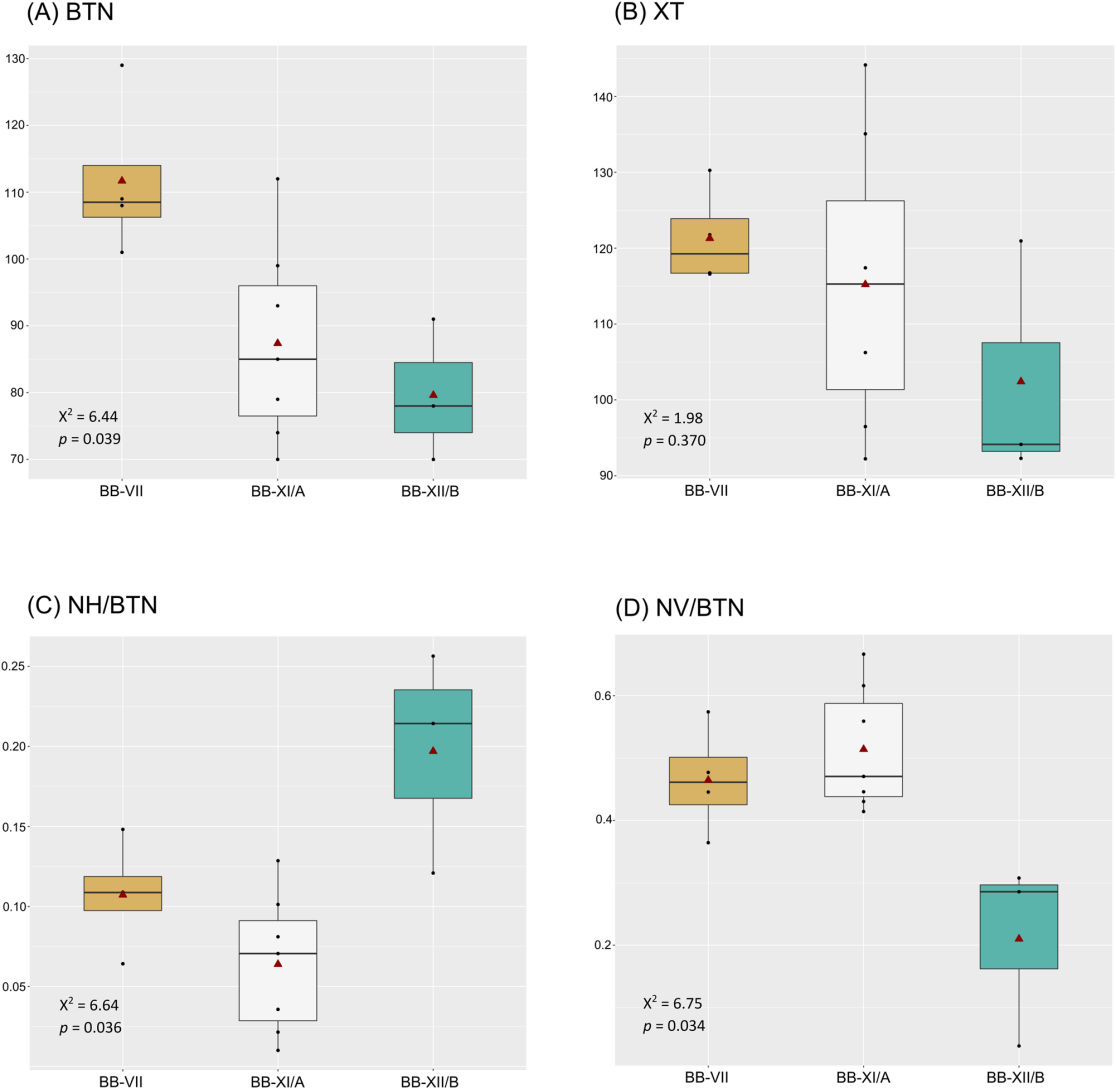
Boxplot representing (**A**) the total number and (**B**) length of striations, (**C**) the vertical and (**D**) horizontal indexes for buccal microwear of Middle Neolithic sites. Samples only include individuals > 8 years old (N = 14; see text for explanation). Red triangles show the means, the horizontal lines represent the medians. Sites: Bükkábrány-Bánya VII (BB-VII, n = 4); Bükkábrány-Bánya XI/A (BB-XI/A, n = 7); Bükkábrány-Bánya XII/B (BB-XII/B, n = 3).

When comparing buccal microwear across sites within each period, only Middle Neolithic samples (N = 14) could be analysed, as only those sites with n ≥ 3 (Bükkábrány-Bánya VII, Bükkábrany-Bánya XI/A and Bükkábrany-Bánya XII/B in Fig. 1B) were included for statistical analyses (see “Methods”). Samples from the Bükkábrány-Bánya VII site had significantly more striations than those from Bükkábrány-Bánya XI/A, (BTN; Kruskal–Wallis: X^2^ = 6.44, *p* = 0.039). We also found differences in the horizontal (NH/BTN; Kruskal–Wallis: X^2^ = 6.65, *p* = 0.036) and vertical indexes (NV/BTN; Kruskal–Wallis: X^2^ = 6.75, *p* = 0.034) between the Bükkábrány-Bánya XI/A and Bükkábrány-Bánya XII/B sites (Fig. 3, Supplementary Table S4).

Differences in buccal microwear between females and males were not significant in any of the comparisons (Supplementary Table S5, Supplementary Figs. S3 and S4). Nevertheless, although non-significant, we found trends in the length of striations (XT) and the horizontal index (NH/BTN) during the Middle Copper Age (Supplementary Fig. S4).

The descriptive data for occlusal surfaces is available in Supplementary Table S2. As in buccal surface, 65 teeth were also analyzed. However, just in 34 adult individuals dental microwear could be observed. In contrast to buccal microwear, we found no significant differences in occlusal dental microwear by period, site, or sex (Supplementary Tables S6–S8, Supplementary Figs. S5–S8).

### Stable isotopes analysis

The carbon and nitrogen stable isotope results of faunal and human samples are summarised in Tables 5 and 6 and are detailed in Supplementary Tables S9 and S10.

**Table 5:** Summary of isotopic results of faunal individuals included in this study.

	Groups	N	δ^13^C‰				δ^15^N‰			
			Mean	SD	Min	Max	Mean	SD	Min	Max
Fauna	Domesticates	18	− 20.5	0.6	− 21.8	− 19.5	8.1	1.0	6.3	10.5
	Wild	4	− 21.6	1.0	− 22.6	− 20.2	6.1	0.4	5.5	6.4
	Fish	1	− 22.9	–	–	–	11.7	–	–	–
Domesticates by species	Cattle	6	− 20.3	0.6	− 21.1	− 19.7	7.6	0.8	6.3	8.4
	Pig	6	− 20.8	0.5	− 21.8	− 20.3	8.9	1.0	7.8	10.5
	Sheep/goat	6	− 20.3	0.7	− 21.3	− 19.5	7.8	0.6	7.4	9.0

Domesticates includes ovicaprids, cattle and pig.

*N* total number of individuals, *SD* standard deviation (σ), *Min* minimum value, *Max* maximum value.

**Table 6:** Summary of isotopic results of human individuals included in this study.

	Groups	N	δ^13^C‰				δ^15^N‰			
			Mean	SD	Min	Max	Mean	SD	Min	Max
By period	Middle Neolithic	40	− 20.2	0.3	− 20.9	− 19.6	11.0	0.8	9.5	13.2
	Middle Copper Age^a^	22	− 20.1	0.3	− 20.9	− 19.6	10.2	0.8	6.9	11.5
	Late Copper Age	4	− 20.4	0.3	− 20.7	− 19.9	11.6	0.7	10.6	12.3
	Middle Bronze Age	17	− 19.4	1.4	− 21.2	− 16.7	10.7	0.9	8.9	12.2
	Late Bronze Age	7	− 16.8	1.3	− 18.1	− 14.9	10.9	1.1	9.8	13.0
By site	Middle Neolithic									
	Bükkábrány-Bánya VII	8	− 20.4	0.2	− 20.5	− 19.9	10.6	0.4	9.9	11.0
	Bükkábrány-Bánya X	1	− 20.9	–	–	–	11.6	–	–	–
	Bükkábrány-Bánya XI/A	13	− 20.3	0.3	− 20.9	− 19.6	11.0	1.0	9.5	13.2
	Bükkábrány-Bánya XII/A	1	− 20.5	–	–	–	11.4	–	–	–
	Bükkábrány-Bánya XII/B	11	− 20.1	0.3	− 20.8	− 19.6	11.5	0.6	10.9	12.5
	Rásonysápberencs-Szőlő alja	3	− 20.0	0.2	− 20.3	− 19.9	9.9	0.3	9.6	10.2
	Arnót-Nagy-bugyik	2	− 20.4	–	–	–	11.4	–	–	–
	Arnót-Arnóti-oldal Dél	1	− 20.0	–	–	–	10.1	–	–	–
	Middle Copper Age									
	Bükkábrány-Bánya XI/B	14	− 20.2	0.4	− 20.9	− 19.6	10.2	1.1	6.9	11.5
	Mezőkövesd-Klementina (Szentistván-Reptér)	2	− 19.9	–	–	–	10.4	–	–	–
	Mezőkövesd-Patakra járó dűlő	6	− 19.9	0.3	− 20.2	− 19.5	10.3	0.3	10.1	10.8
	Late Copper Age									
	Bükkábrány-Bánya XII/A	2	− 20.7	–	–	–	11.5	–	–	–
	Bükkábrány-Bánya XII/B	2	− 20.1	–	–	–	11.9	–	–	–
	Middle Bronze Age									
	Mezőzombor-Községi temető	10	− 19.6	1.5	− 21.2	− 16.7	10.9	1.0	8.9	12.2
	Mezőkeresztes-Csincse-tanya	1	− 16.7	–	–	–	10.6	–	–	–
	Nagyrozvágy-Papdomb	1	− 21.4	–	–	–	11.1	–	–	–
	Vatta-Dobogó	5	− 19.9	1.2	− 21.0	− 18.1	9.9	1.9	6.9	11.5
	Late Bronze Age									
	Felsődobsza-2. lelőhely	1	− 15.2	–	–	–	10.8	–	–	–
	Oszlár-Nyárfaszög (M3-32. lelőhely)	1	− 16.8	–	–	–	10.9	–	–	–
	Mezőkeresztes-Cethalom (M3-10. lelőhely)	1	− 17.5	–	–	–	10.3	–	–	–
	Köröm-Kápolnadomb	1	− 18.0	–	–	–	9.9	–	–	–
	Pácin-Alsókenderszer	3	− 16.8	1.7	− 18.1	− 14.9	11.5	1.6	9.8	13.0
By sex	All samples									
	Female	22	− 19.9	0.8	− 20.7	− 17.2	10.7	0.9	9.5	12.3
	Male	27	− 19.7	1.3	− 15.2	12.5	10.9	0.7	9.8	12.5
	Middle Neolithic									
	Female	7	− 20.2	0.2	− 20.4	− 19.9	10.4	0.8	9.5	11.8
	Male	11	− 20.2	0.2	− 20.5	− 19.7	10.9	0.8	9.9	12.5
	Middle Copper Age									
	Female	5	− 19.9	0.3	− 20.3	− 19.6	10.2	0.3	9.8	10.6
	Male	5	− 19.8	0.2	− 19.9	− 19.5	10.7	0.5	10.1	11.5
	Middle Bronze Age									
	Female	6	− 19.4	1.4	− 20.2	− 17.2	10.9	0.9	9.5	11.5
	Male	7	− 20.5	0.7	− 21.2	− 19.5	11.3	0.5	10.9	12.2

Middle and Bronze Age individuals were previously published in^22^.

*N* total number of individuals (including both adults and young individuals), *SD* standard deviation (σ), *Min* minimum values, *Max* maximum value.

^a^HUNG896 was not included.

The local terrestrial fauna analysed (N = 26; Fig. S9) had a range of δ^13^C of − 22.6 to − 17.9‰ [mean = − 20.5‰ ± 1.0‰ (1σ)], and a range of δ^15^N of 5.5–11.0‰ (mean = 7.9‰ ± 1.4‰ (1σ)), similar to those previously reported in the area^21,102,103^. Significant δ15N values are found among domesticates (Kruskal–Wallis: X^2^ = 6.21, p = 0.044), and particularly pigs show higher enriched δ^15^N values than sheep/goats (Mann–Whitney: W = 32.50, p = 0.024). High δ^15^N values are also seen in the GHP site of Polgár-Ferenci-hát^104^, suggesting they might eat a fair amount of meat and most likely being fed human food scraps.

**Figure 4:**
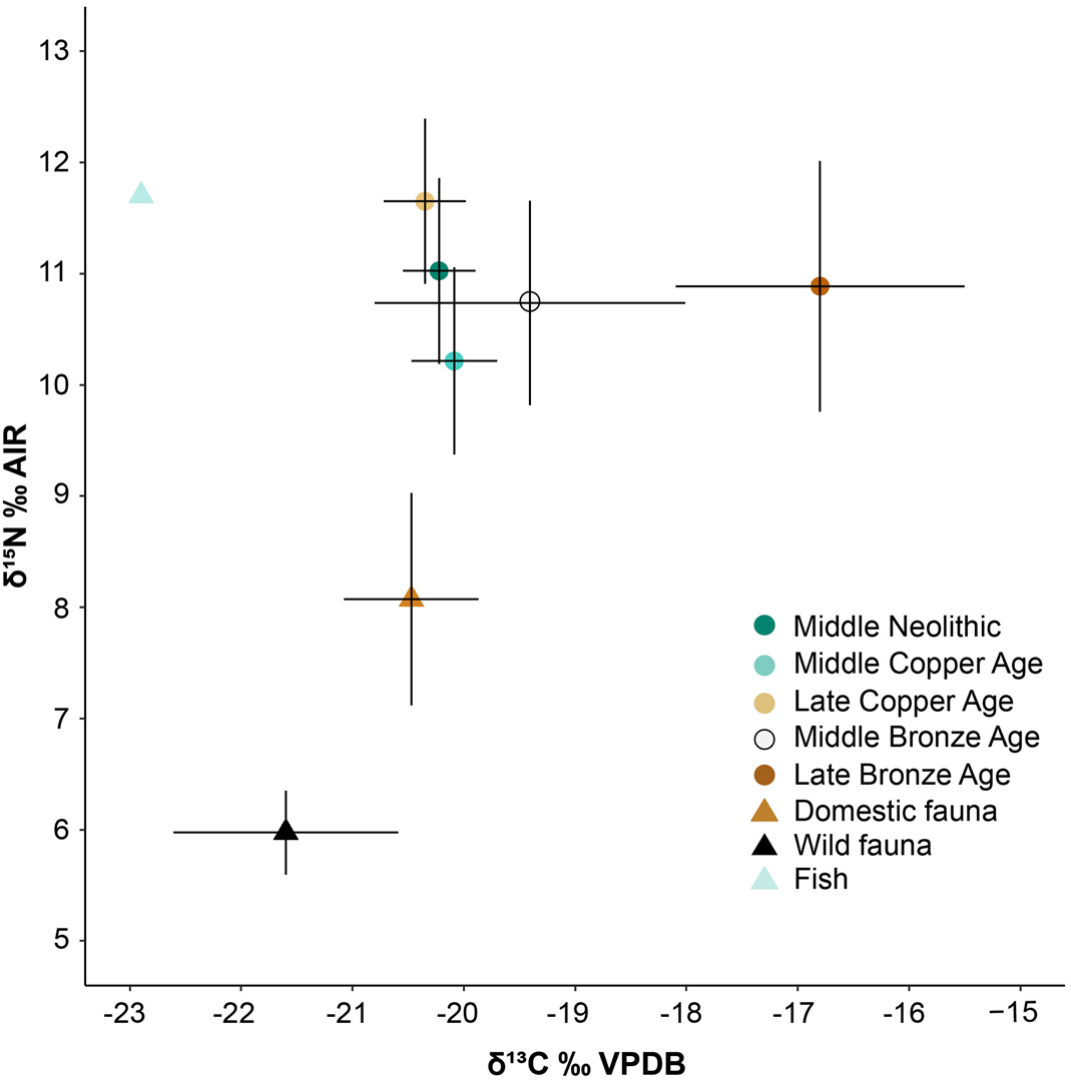
Average values of stable carbon and nitrogen isotope ratios of human and faunal bone collagen from sites analysed in this study. Human samples include both adults and young individuals (N = 89). Human isotopic values from Middle and Late Bronze Age sites were previously published in^22^. Domestic fauna includes ovicaprids, cattle and pigs. Errors bars correspond to the standard deviation (1σ).

The range of δ^13^C values for the human sample, including adults and infants (N = 89; Fig. 4), was − 21.2 to − 14.8‰ [mean = − 19.8‰ ± 1.1‰ (1σ)], and the range of δ^15^N was 6.9–13.2‰ [mean = 10.8‰ ± 0.9‰ (1σ)]. The human sample HUNG896 had an unusually low δ^15^N value (Supplementary Table S10), which indicates low consumption of animal protein and/or exclusively plant protein source^105^, or reflects a positive nitrogen physiological balance (more protein is used for tissue formation and less nitrogen is excreted), as seen in different physiological conditions (e.g. pregnancy, liver disease or growth spurts) in soft tissues (i.e. hair)^82^. Although it still remains uncertain the detection of physiological stress in archaeological skeletal remains^82^, presumably this individual might have suffer some kind of physiological stress occurred during a long term (as it is reflected in bone collagen), or at least episodes of nutritionally deficient diets during early years as suggested by the presence of linear enamel hypoplasia in the anterior teeth^106,107^. That is why we decided to remove this individual from statistical analyses. Some infant individuals showed high δ^15^N values (HUNG892 and HUNG967), most probably due to breastfeeding effect^83,108^. Therefore, Infants I (1–6 years^109^) were also excluded from statistical analyses (see “Methods”).

**Figure 5:**
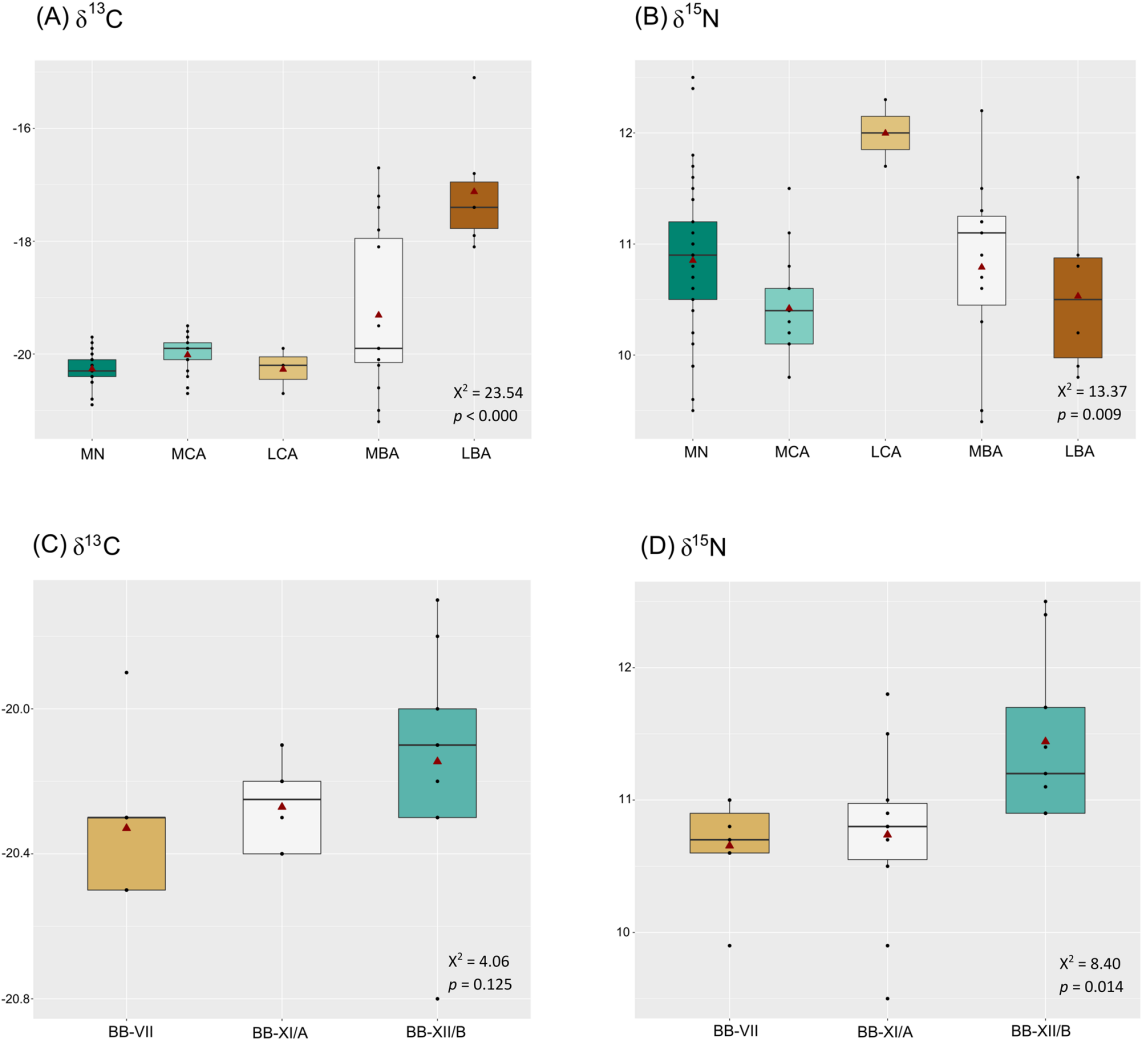
Boxplot showing δ^13^C and δ^15^N values for human samples by period (N = 74) (**A**, **B**) and by Middle Neolithic sites (N = 26) (**C**, **D**). Samples only include individuals ≥ 6 years old (see text for explanation). Red triangles show the means, middle horizontal lines represent the medians. Human isotopic values from Middle and Late Bronze Age sites were previously published in^15^. Middle Neolithic (MN, n = 33); Middle Copper Age (MCA, n = 17); Late Copper Age (LCA, n = 3); Middle Bronze Age (MBA, n = 15); Late Bronze Age (LBA, n = 6). Sites: Bükkábrány-Bánya VII (BB-VII, n = 7); Bükkábrány-Bánya XI/A (BB-XI/A, n = 10); Bükkábrány-Bánya XII/B (BB-XII/B, n = 9).

Ideally, humans’ isotope ratios should be compared relative with other fauna from the same site and specific period of time. However, the faunal sample size available was relatively small to be compared separately for each time period. We decided to group all faunal samples since apparently they do not differ between periods (Supplementary Fig. S9) and the isotopic range found for each of the species are similar to those found in other GHP sites from different periods (Table 5)^103,104^. With the data employed here, most of the human’s samples potentially fall within the assumed isotopic offset range estimated from the domesticated species used here (ovicaprids, cattle, and pigs). When comparing human samples between different periods (N = 74), significant differences appeared between the isotope ratios (δ^13^C Kruskal–Wallis: X^2^ = 23.54, *p* < 0.000; δ^15^N Kruskal–Wallis: X^2^ = 13.37, *p* = 0.009). Individuals from the Middle Neolithic to the Late Copper Age had similar δ^13^C values consistent with a diet based on terrestrial C_3_ resources (Fig. 5). In contrast, individuals from the Late Bronze Age had significantly higher δ^13^C values (p < 0.05) (Supplementary Table S11) than those in preceding periods, in accordance with previous studies^21,22^. Nitrogen isotope ratios presented a wide range of values, suggesting a varying amount of animal protein intake (Fig. 5). Late Copper Age δ^15^N values were significantly higher compared to the rest of the time periods, and the Middle Copper Age samples presented significantly lower δ^15^N values than their Middle Neolithic predecessors (Supplementary Table S12).

When comparing isotope ratios by site for each period, only those sites with n ≥ 3 were included for statistical analyses. Late Neolithic, Late Copper Age and Late Bronze Age periods were not used in this analysis as they were represented by either a single individual per site or one site per period (Table 6). We found significant statistical differences in δ^15^N values between Middle Neolithic sites (Bükkábrány-Bánya VII, Bükkábrany-Bánya XI/A and Bükkábrany-Bánya XII/B in Fig. 1B); Kruskal–Wallis: X^2^ = 8.40, *p* = 0.014), but no differences in δ^13^C values (Kruskal–Wallis: X^2^ = 4.06, *p* = 0.125). Differences were significant between Bükkábrány-Bánya XII/B and the other sites (Fig. 5, Supplementary Table S13). However, we found no significant statistical differences among Middle Copper Age sites (Bükkábrány-Bánya XI/B and 7-Mezőkövesd-Patakra járó dűlő in Fig. 1A; δ^13^C Mann–Whitney: W = 17.50, *p* = 0.599; δ^15^N Mann–Whitney: W = 26.50, *p* = 0.596) (Supplementary Fig. S10). Middle Bronze Age sites (8-Mezőzombor-Községi temető and 11-Vatta-Dobogó in Fig. 1A) only presented significant differences for δ^15^N values (δ^13^C Mann–Whitney: W = 8.00, *p* < 0.090; δ^15^N Mann–Whitney: W = 37.00, *p* = 0.015). Notably, markedly higher δ^13^C values from the Vatta-Dobogó site indicate a signature indicative of C_4_ plant consumption (Supplementary Fig. S11), suggesting a differential intensification of crops during that time period.

Differences between females and males (N = 49) were not statistically significant for either δ^13^C or δ^15^N values (δ^13^C Mann–Whitney: W = 284.5, *p* = 0.808; δ^15^N Mann–Whitney: W = 218.5, *p* = 0.116). These were not statistically different when comparing females and males by period (Supplementary Table S14, Supplementary Fig. S12).

## Discussion

In Middle Neolithic to Late Bronze Age populations that lived in the GHP, dental microwear and stable isotope analyses have been shown to provide complementary information rather than correlated variables. Microwear data from both occlusal and buccal surfaces show the abrasiveness of food, which might be related to cultural and technological changes such as food processing or dietary preferences^42,44,46,53,96,110^. By contrast, isotope data indicate the types of resources consumed by individuals^63,78^. Each approach describes dietary patterns at different levels. Therefore, they can be combined in paleodietary research to explore dietary preferences in past populations at short (occlusal microwear) and long (buccal microwear and stable isotopes combined) timescales, within individuals and across groups^46,89,90^.

There were significant positive and negative correlations between δ^15^N and the buccal variables of vertical (NV/BTN) and horizontal (NH/BTN) index, respectively. This means that individuals with high δ^15^N values (presumably with high meat consumption) are characterized as having a relatively more vertical and less horizontals striations. These results are in accordance with studies suggesting meat-eaters usually present high frequencies of vertical striations on the enamel surface due to the movements of the mandible while chewing meat^49,111^. In contrast, chewing hard foods, such as cereals, requires more horizontal movements^49,111^, which increases the horizontal index.

Generally, we did not find significant differences between periods, sites or sex in occlusal dental microwear. The lack of differences in the occlusal surfaces might be due to the so-called “last supper effect”, which consists of a fast renewal of occlusal enamel, thus recording the diet of the most recent days or weeks^48,93,112^. This contrast with a slower turnover of the buccal surface, which stabilizes at maturity^50,54^, and provides information of the diet over time. Nevertheless, we found interesting trends among the samples on the occlusal surface, which we discuss below.

We observed significant diachronic differences between the Middle Neolithic and the rest of the periods in the buccal microwear analysis, with the exception of Late Copper Age individuals. The low number of striations (BTN) observed from the Middle Neolithic might indicate that foods were highly processed, and thus less abrasive, and/or that a high proportion of consumed foods were soft, such as meat, which left little wear on the enamel surface^44,49,59,96,110,111^. The high nitrogen isotopic values [mean = 11.0 ± 0.8‰ (1σ)] were similar to those found at other Middle Neolithic sites^21,104^. Freshwater resources consumption (e.g. fish) can potentially explain high δ^15^N values, due to longer and complex foodwebs^77,113^, and to its proximity to freshwater resources access for some of the sites. However, although not completely discarded, the consumption of freshwater fish would not necessary lead to significant nitrogen isotope enrichment. Even if the freshwater fish represented 20% of human’s diet, the isotopic effect is quite small (+ 0.7‰)^66^. Additionally, freshwater fish consumption would have resulted in a more ^13^C-depleted values, as documented in other Mesolithic and Early Neolithic individuals from the same geographical area^21,114^. The carbon isotopic signal from bone collagen of Middle Neolithic sites are indicative of a C_3_ terrestrial protein source.

Assuming a range for trophic level enrichment generally accepted in the literature and observed in the archaeological record of the GHP^102,103^ (ca 0–1‰ for δ^13^C and ca 2–3‰ for δ^15^N), high δ^15^N could be explained by high reliance on meat consumption, particularly pig meat with significant high enriched ^15^N ratios among domesticates. However that would have required that 100% of the protein in diet had contributed essentially from terrestrial mammalian fauna^66^, ignoring that other dietary macronutrients, such as carbohydrates and lipids, are required to meet body’s energy requirements^115,116^. Another possibility is the ingestion of young animals meat, which shows higher δ^15^N due to, most probably, nursing signal^117^. Different patterns on bone assemblages were found on Middle Neolithic sites of the northern part of the GHP. Findings in Nagykálló-Harangod yielded several young animals (mostly sheep)^118^, while in Kompolt-Kíser high amounts of elderly individuals were found, probably due to their complex exploitation (skin meet, wool, milk and/or meat)^119^. Unfortunately, the zooarchaeological small sample size makes difficult assess the extent to which livestock exploitation prevailed in the sites presented in this study. High δ^15^N values may be the result of a combined effect of fertilized crops and meat consumption, as suggested elsewhere^21,80,103^. Long-term experimental studies have shown an increase in δ^15^N values in modern manured crops^80,120^. This has led some researchers to suggest that there has been an overestimation of animal protein intake in Neolithic paleodietary reconstructions^121^. Thus, these researchers propose that the combined effects of fertilized crops and animal protein consumption in Early Neolithic European farmers should result in bone collagen δ^15^N values of approximately 9–11‰^80,121^. For Middle Neolithic samples represented here, manured crops consumption, together with animal protein intake, in our opinion, is the hypothesis more feasible to us to explain high δ^15^N values.

However, we found that there are differences in buccal microwear and isotope ratios among Middle Neolithic sites. Particularly, samples from the Bükkábrány-Bánya VII site tended to have more total buccal striations (BTN) when compared to other Middle Neolithic sites, which were significantly different from those found at Bükkábrány-Bánya XI/A. This might indicate that the diet at Bükkábrány-Bánya VII was more abrasive due to the consumption of hard foods and/or to the inclusion of hard extrinsic particles in food^50,53,55^. Bükkábrány-Bánya XII/B samples instead presented the lowest BTN, indicating that the foods in their diets were less abrasive. Although not statistically significant, we observed the same trend for the total number of occlusal striations in both Middle Neolithic sites. This is also in accordance with δ^15^N values, where in generally high isotopic values were observed for all Middle Neolithic sites; in particular, we further found significantly more consumption of meat, a minimally abrasive resource, by the individuals at Bükkábrány-Bánya XII/B. Although we found significant differences between Bükkábrány-Bánya XI/A and Bükkábrány-Bánya XII/B sites in the horizontal and vertical indexes, the values of both indexes within Bükkábrány-Bánya XII/B were similar (around 0.2%), indicating they ate a mixed diet. Differences between the Middle Neolithic sites might have been due to cultural differences, particularly the type of food intake and/or how food was processed (e.g., with millstones) or cooked (e.g., in pottery vessels), as has been observed in other prehistoric populations^37,42,44,122^. It is important to keep in mind that Bükkábrány-Bánya XI/A and Bükkábrány-Bánya XII/B are closer to each other than either of them is to Bükkábrány-Bánya VII^123^ (Fig. 1B), which may have affected cultural and economic patterns, as well as connections across the sites. With the information recovered from the distribution of grave goods, dietary pattern differences between Bükkábrány-Bánya sites cannot be attributed to differences in terms of social status. Further studies are needed to assess whether there were other differences in these settlements.

Both buccal microwear and δ^15^N values suggest that there was a change in dietary preferences among Middle Copper Age individuals. Significantly higher BTN values than those found in Middle Neolithic individuals indicate they were consuming a more abrasive diet such as cereals, or to the presence of exogenous particles, such as grit, as a result of processing or cooking methods^44,50,57,58^. In keeping with this finding, lower δ^15^N values also indicate lower animal protein intake, in accordance with a lower reliance on softer resources, and/or on manured crops. Isotopic faunal offsets suggest that potentially they could have relied on cattle, pig and sheep/goats as an animal protein source, although only ovicaprid remains were recovered from this period in this study. In contrast, Late Copper Age individuals presented significant δ^15^N-rich nitrates compared to other periods, suggesting a higher meat intake (potentially from all domesticated fauna represented in this study), and/or consumption of fertilized crops or animals that consumed them^80,103^. Late Copper Age individuals had lower BTN values, which is related to less abrasive foods, such as meat^110^. Additionally, despite not being significant, there was a tendency for the values of the vertical indexes on the buccal surfaces to be high, which also might indicate that there was more consumption of meat^49^. The nitrogen isotope signal in these Late Copper Age individuals was noticeably higher (mean = 12.0 ± 0.3‰ (1σ)) than those from other Late Copper Age sites^21^. These results are in accordance with findings from other Late Copper Age sites that suggest an increase in animal product use during this period^16^, but not from other Middle Copper Age sites^103^. As Giblin^103^ argued for Early Copper Age site differences, this could be due to regional site differences in meat consumption and nitrogen enrichment, rather than differences in subsistence practices. More Copper Age sites, particularly from the Middle and Late Copper Age, need to be analysed to better understand if the increase in animal products was a general trend or instead a local or regional process.

The few Middle and Late Bronze Age individuals analysed for buccal microwear presented high mean values of BTN and XT variables, which indicates the consumption of an abrasive diet^50,55^ and/or less consumption of processed food^43^. Millet consumption starts to be noticeable in bone collagen as δ^13^C values suggest^22^, although it probably would have been identified in earlier and more individuals if bone or enamel apatite were also tested (see^60^). Higher consumption of this C_4_ plant resource was not probably the cause of the abrasiveness increase, since no significant positive correlations were found for BTN and XT with δ^13^C values. The nitrogen isotope signal, although high (MBA mean: 10.8 ± 0.9‰ (1σ); LBA mean: 10.9 ± 1.1‰ (1σ)), suggests a decreased reliance on animal products (potentially from the livestock represented here), and/or manured crops as compared to Late Copper Age individuals^22^.

There were no significant sex differences among any of the periods analyzed. Despite a limited sample size, there was an overarching trend in XT and NH/TN in the Middle Copper Age individuals for females and males: females presented shorter, more horizontally oriented buccal striations than males, which is consistent with higher cereal intake. Males’ higher δ^15^N values suggest they may have consumed more animal protein than females. This trend is supported by other studies in Holocene populations^45,124^. This issue needs more careful research, and additional studies using dental microwear and stable isotopes should be undertaken to explore potential sex-related differences and their dietary manifestations.

## Conclusion

As demonstrated here, the integration of dental microwear and stable isotope approaches employed within individuals can provide a more complete overview of the dietary preferences of past human populations. Our findings suggest these two proxies are complementary, confirming their significance in paleodietary research, and we encourage future studies its combined use.

In general, the level of abrasiveness in diet, revealed by buccal dental microwear variables, is correlated with the isotopic signal at the individual/local level when interpreting δ^15^N results for meat consumption. In addition, the results obtained here show that changes in C_4_ plant consumption in the prehistoric Hungarian populations did not necessarily entail changes in diet abrasiveness, either in the C_4_ resource itself (grain) or the way it is processed (extrinsic particles). It is the case of Middle Bronze Age sites where higher reliance on millet (C_4_) consumption did not imply differences in the abrasiveness of their diet.

This research demonstrates the importance of studying the variation between different sites from the same time period. We have shown the characteristics of each Middle Neolithic site, and, at the individual level, confirmed that both dietary proxies, dental microwear and stable isotopes, point toward the same interpretation. We also found that previous interpretations regarding an increase in the consumption of animal products by the Middle Copper Age could not be confirmed in the Middle Copper Age sites analysed here.

We found no significant differences in dietary composition between females and males for each period. However, results for Middle Copper Age females and males suggest a differential trend in proportions of cereal and meat consumed. Further research involving bone apatite and/or tooth enamel, as well as the inclusion of more samples would contribute significantly to better understand these results.

## Methods

### Dental microwear analysis

Prior to microscopic analysis, molars were washed with cotton swabs soaked in 96% alcohol, then left to air dry completely^125^. Polyvinylsiloxane dental impression material (Coltene President Ligh Body^©^) was used to mold the tooth crowns. High resolution casts were made using EPO-TEK 301. The replicas were sputter coated with gold, and aluminum tape was attached from the stub to the molar to improve the conductivity of electrons^126^. We analysed the buccal and occlusal surfaces of the molars under an FEI Quanta 600 Environmental Scanning Electron Microscope (ESEM) held in the Scientific and Technical Resources Service of the University Rovira i Virgili (Tarragona, Spain), using previously-published parameters^43^.

#### Analysis of buccal microwear

Micrographs (1024 × 832 pixels) were taken with ESEM at 100× of the middle third of the surface. The images were enhanced to increase the contrast and facilitate the observation of striations^125^ with the free software GIMP (version 2.10.22), (https://gimp.es/). Then, digital images were cropped to cover an area of 0.56 mm^2^ following^125^. Afterwards, the total number of striations in the buccal surface (BTN), the average length (XT, in µm), and their orientation (0°–180°, divided into 45° intervals according to their orientation: horizontal (H) (0°–22.5°; 157.5°–180°), vertical (V) (67.5°–112.5°), mesiodistal (MD) (Lower Left Molar/Upper Right Molar [LL/UR]: 112.5°–157.5°; Upper Left Molar/Lower Right Molar [UL/LR]: 22.5°–67.5°); distomesial (DM) (Lower Left Molar/Upper Right Molar [LL/UR]: 22.5°–67.5°; Upper Left Molar/Lower Right Molar [UL/LR]: 112.5°–157.5°)^50^ were computed using open-source image processing software, Image J^127^ (version 1.52p), (https://imagej.nih.gov/ij/index.html). Two orientation indexes that track broad differences in dietary patterns across groups^49,50^ were calculated: total horizontal/total number (NH/BTN) and total vertical/total number (NV/BTN).

#### Analysis of occlusal microwear

Micrographs (1024 × 832 pixels) were taken with ESEM at 500× of facet 9, which is located on the distobuccal cusp on the first and second molars^128,129^. Facet 9 is a crushing and grinding surface, which experiences both compression and shearing during chewing^130,131^. The images were also enhanced, and the contrast modified using GIMP. Each image was cropped to 700 × 500 pixels, which represents approximately 0.03 mm^2^ of the molar’s surface^112,122^. On this surface, four variables were considered: the number of striations (OTN), the number of pits, their area, and the percentage of pits (Pits/(TN + Pits) × 100)^42^. Striations were defined and measured as in buccal surface, whereas pits are defined as features with a length to width ratio ≤ 4:1^93^. Both features (striations and pits) were computed and recorded with ImageJ.

#### Observer error

There is a potentially high level of inter-observer error of the dental microwear^132^. For that reason, one author (RH) was responsible for the measurement of buccal and occlusal dental microwear.

### Collagen extraction and isotope analyses

Carbon and nitrogen stable isotope analyses were carried out on human bones from different individuals of both sexes and various ages, along with several faunal bones (Table 2 and Supplementary Tables S9 and S10). Previously published isotopic data from Bronze Age samples^22^ (24 individuals) were also included in the analyses. Human postcranial samples were preferentially selected for collagen extraction except in those cases where only skull fragments were available (Supplementary Table S10).

Sample preparation and collagen extractions were performed at the University College Dublin (UCD) School of Archaeology lab facilities and UCD Conway Institute (Dublin, Ireland). Bone fragments were prepared by cutting a sample of approximately 0.2–1.0 g from the original sample material using a handheld rotary tool with a diamond-coated cutting wheel. The outer surfaces were then abraded using a diamond coated burr. Collagen extraction followed a modified Longin method^133^. Bone samples were first weighed and then demineralized in 0.5 M HCl at 4 °C until soft and pliable. Demineralization time varied between bone samples, from just over a week to 3 weeks. Samples were then rinsed in deionized water three times and gelatinized in pH 3 HCl solution at 70 °C for roughly 48 h. The resulting gelatine solutions were filtered using Ezee filters and then freeze-dried. Subsequently, aliquots of approximately 0.35–0.60 mg were prepared in duplicate in tin capsules for mass spectrometry analyses.

Stable isotope analyses were carried out following the routine procedures at Light Stable Isotope Mass Spectrometry Laboratory of the Department of Geological Science at University of Florida (Gainesville, USA). A Thermo Electron DeltaV Advantage isotope ratio mass spectrometer was used coupled with a ConFlo II interface linked to a Carlo Erba NA 1500 CNS Elemental Analyzer. The accuracy and precision of the measurements, based on repeated measurements of two international laboratory standards USGS40 and USGS41, was ± 0.05‰ (1σ) for δ^13^C and ± 0.06‰ (1σ) for δ^15^N. All δ^13^C results are expressed in standard delta notation relative to Vienna PeeDee Belemnite (V-PDB). All δ^15^N results are expressed in standard delta notation relative to air N_2_ (AIR).

### Ancient DNA analyses

Ancient DNA (aDNA) analyses were performed using human petrous bones following standard procedures to determine the sex of some of the individuals (Table 2). The cochlea of one petrous bone from each individual was isolated with a sandlaster^134,135^. Powder was obtained from the isolated cochlea using a mixer mill (Retsch MM400), and aliquots of 50–75 mg were used for DNA extraction. The risk of modern DNA contamination was reduced by carrying out all steps in a lab dedicated to the preparation of ancient bone samples at the UCD Conway Institute of Biomolecular and Biomedical Research (University College Dublin, Ireland), as recommended by^136–138^. While handling ancient bone samples, researchers wore overall suits, double gloves, hair nets, and face masks; all surfaces were cleaned and decontaminated with DNA-ExitusPlus between sample preparation and UV irradiation was also used to ensure effective decontamination took place.

The DNA extraction, library preparation, and sequencing steps for Neolithic and Copper Age samples were performed in a physically separated aDNA lab at UCD and at the University of Vienna, following standard stringent anti-contamination protocols. A detailed description of DNA extraction, library preparation, and sequencing steps is provided in the “Supplementary Information Methods”. For Bronze Age samples, these steps were performed at Harvard University as part of a separate study.

### Statistical analyses

Given the small sample size of some chronological groups, the use of non-parametric statistic was required^139^. Although non-parametric tests were done, with this kind of sample we also relied on overarching trend and data visualization when the groups were small^140,141^.

To investigate the relationship between microwear and stable isotope approaches, Spearman correlation tests were performed between microwear variables, and δ^13^C and δ^15^N, for each individual. Biplot graphs were generated for graphical visualization, including Ordinary Least Squares (OLS) regressions with a 95% confidence interval (Supplementary Figs. S1 and S2).

For general dietary trends, human samples were compared from diachronic (between time periods), synchronic (between sites), and between-sex perspectives by employing non-parametric statistical analyses. Isotopic values from faunal samples were also statistically compared. Kruskal–Wallis tests were chosen when comparing more than two groups (e.g. between periods). Mann–Whitney tests were applied for pairwise comparisons (i.e. compare two groups).

For dental microwear, only individuals 8 years and older were considered for statistical analyses, as children over 8 years have been shown to have similar dietary patterns to adults within the same time periods^92^. For isotope analyses, only data from adults and subadults 6 years and above of known age were considered, as they have been shown to have similar isotopic signals in the same geographical areas and time periods^103^. Boxplots were created to illustrate the general tendencies of the samples. Human isotope ratios were also compared with fauna isotope ratios. Faunal samples from all sites were grouped and compared with human samples for each time period separately. We assumed an isotopic fractionation range from consumed to consumers of a + 2–3‰ for δ^15^N and + 0–1‰ for δ^13^C, as it is the range that generally previous studies have found^103,104^. All statistical data was performed with R^142^, and graphs were generated using the package ggplot2^143^. All data generated are included in this article (and in “Supplementary Information” files).

### Ethics

All necessary permits were obtained for the described study, which complied with all relevant regulations and ethical approval (Herman Ottó Múzeum).

## Supplementary Information

Supplementary Information 1.

Supplementary Information 2.

Supplementary Information 3.

Supplementary Information 4.

Supplementary Information 5.

Supplementary Information 6.

Supplementary Information 7.

## Data Availability

All data generated or analysed during this study are included in this published article (and its “Supplementary Information files”).
