# Supplementary material for: Integrating buccal and occlusal dental microwear with isotope analyses for a complete paleodietary reconstruction of Holocene populations from Hungary
Source: Sci Rep. 2021 Mar 29;11:7034. doi: 10.1038/s41598-021-86369-x (PMC8007593; doi:10.1038/s41598-021-86369-x)

Supplementary Figures S3-S8

Integrating buccal and occlusal dental microwear with isotope analyses for a complete paleodietary reconstruction of Holocene populations from Hungary.

Raquel Hernando[1,2*], Beatriz Gamarra[2,1,3*], Ashley McCall[3], Olivia Cheronet[4,3], Daniel Fernandes[4,5,3], Kendra Sirak[6,7,3], Ryan Schmidt[8,3], Marina Lozano[2,1], Tamás Szeniczey[9,10], Tamás Hajdu[9,10], Annamária Bárány[11], András Kalli[12], Eszter K. Tutkovics[13], Kitti Köhler[14], Krisztián Kiss[9,10], Judit Koós[15], Piroska Csengeri[15], Ágnes Király[14], Antónia Horváth[15], Melinda L. Hajdú[15], Krisztián Tóth[16], Róbert Patay[17], Robin N. M. Feeney[18], Ron Pinhasi[4]

*Corresponding authors: r.hernando90@gmail.com and beagamarra@gmail.com. These authors contributed equally to this work.

[1]Universitat Rovira i Virgili, Departament d'Història i Història de l'Art, Avinguda de Catalunya 35, 43002 Tarragona, Spain.
[2]Institut Català de Paleoecologia Humana i Evolució Social (IPHES), Zona Educacional 4, Campus Sescelades URV (Edifici W3), 43007 Tarragona, Spain.
[3]School of Archaeology and Earth Institute, University College Dublin, Dublin, Ireland.
[4]Department of Evolutionary Anthropology, University of Vienna, Vienna, Austria.
[5]CIAS, Department of Life Sciences, University of Coimbra, 3000-456 Coimbra, Portugal.
[6]Department of Genetics, Harvard Medical School, Boston, MA 02115, USA.
[7]Department of Human Evolutionary Biology, Harvard University, Cambridge, MA 02138, USA
[8]CIBIO-InBIO, Universidade do Porto, Portugal.
[9]Department of Biological Anthropology, Eötvös Loránd University, Budapest, H-1117 Pázmány Péter sétány 1/c.
[10]Department of Anthropology, Hungarian Natural History Museum, Budapest, H-1083, Ludovika tér 2.
[11]Department of Archaeology, Hungarian National Museum, Budapest, H-1088, Múzeum krt. 14-16.
[12]Várkapitányság Integrált Területfejlesztési Központ Nonprofit Zrt., H-1113 Budapest, Daróczi út 3., Hungary.
[13]Rétközi Museum, H-4600 Kisvárda, Csillag u. 5., Hungary.
[14]Institute of Archaeology, Research Centre for the Humanities, Loránd Eötvös Research Network, Budapest, H-1097 Tóth Kálmán utca 4.
[15]Herman Ottó Museum, H- 3529 Miskolc, Görgey Artúr u. 28, Hungary.
[16]Dornyay Béla Museum, H-3100 Salgótarján, Múzeum tér 2., Hungary.
[17]Department of Archaeology, Ferenczy Museum Center, Szentendre, H-2000 Fő tér 2–5.
[18]School of Medicine, University College Dublin, Dublin, Ireland.

Abbreviations: Middle Neolithic (MN); Late Neolithic (LN); Middle Copper Age (MCA); Late Copper Age (LCA); Middle Bronze Age (MBA); Late Bronze Age (LBA).

MN site abbreviations: Bükkábrány-Bánya VII (BB-VII); Bükkábrány-Bánya XI/A (BB-XI/A); Bükkábrány-Bánya XII/B (BB-XII/B).

Figure S3: Boxplot showing (a) the total number and (b) length of striations, (c) the vertical and (d) horizontal index of buccal microwear by sex (males (n = 5): M; females (n = 3): F) of Middle Neolithic individuals. Red triangles show the means, middle horizontal lines represent the medians. W: Mann-Whitney test; *p*: p-value.

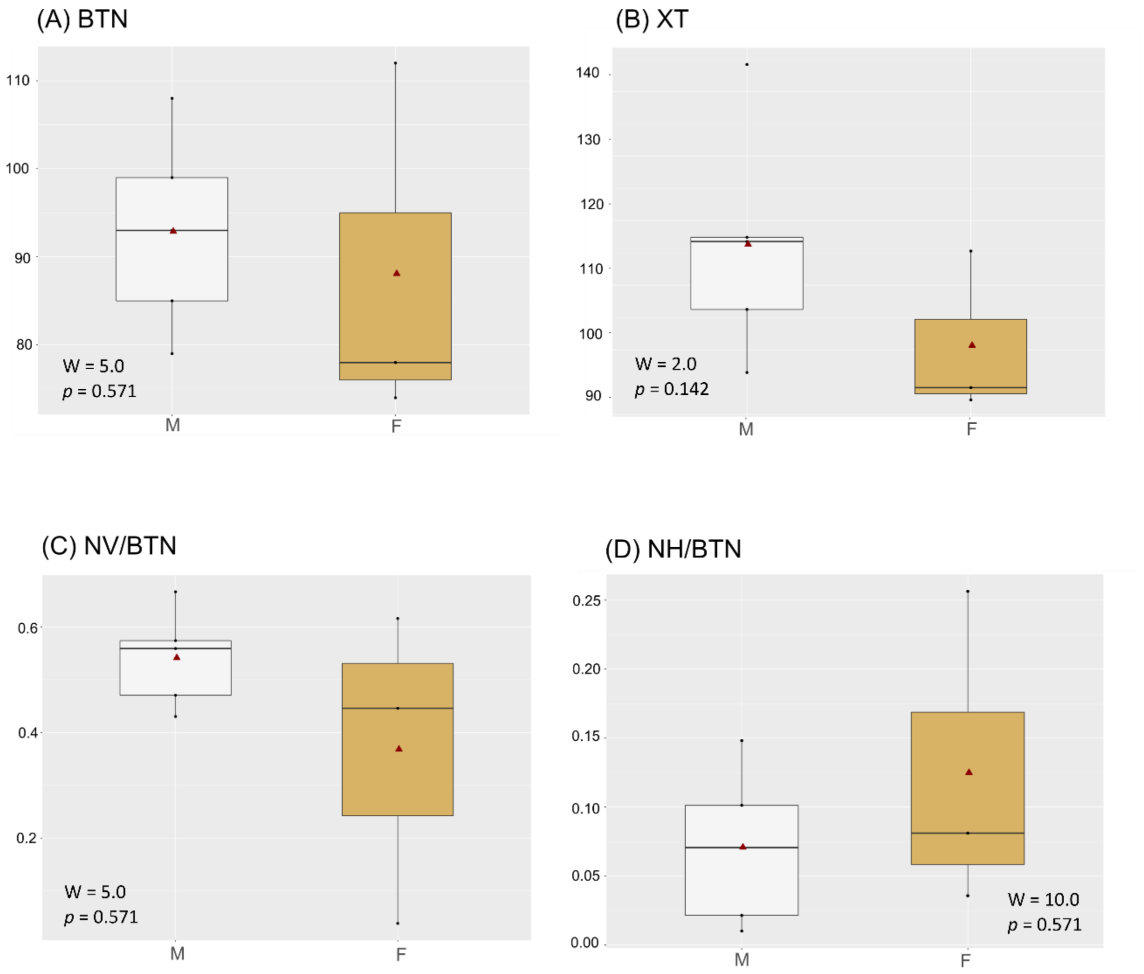

Figure S4: Boxplot showing (a) the total number and (b) length of striations, (c) vertical and (d) horizontal index of buccal microwear by sex (males (n = 2): M; females (n = 3): F) of Middle Copper Age individuals. Red triangles show the means, middle horizontal lines represent the medians. W: Mann-Whitney test; *p*: p-value.

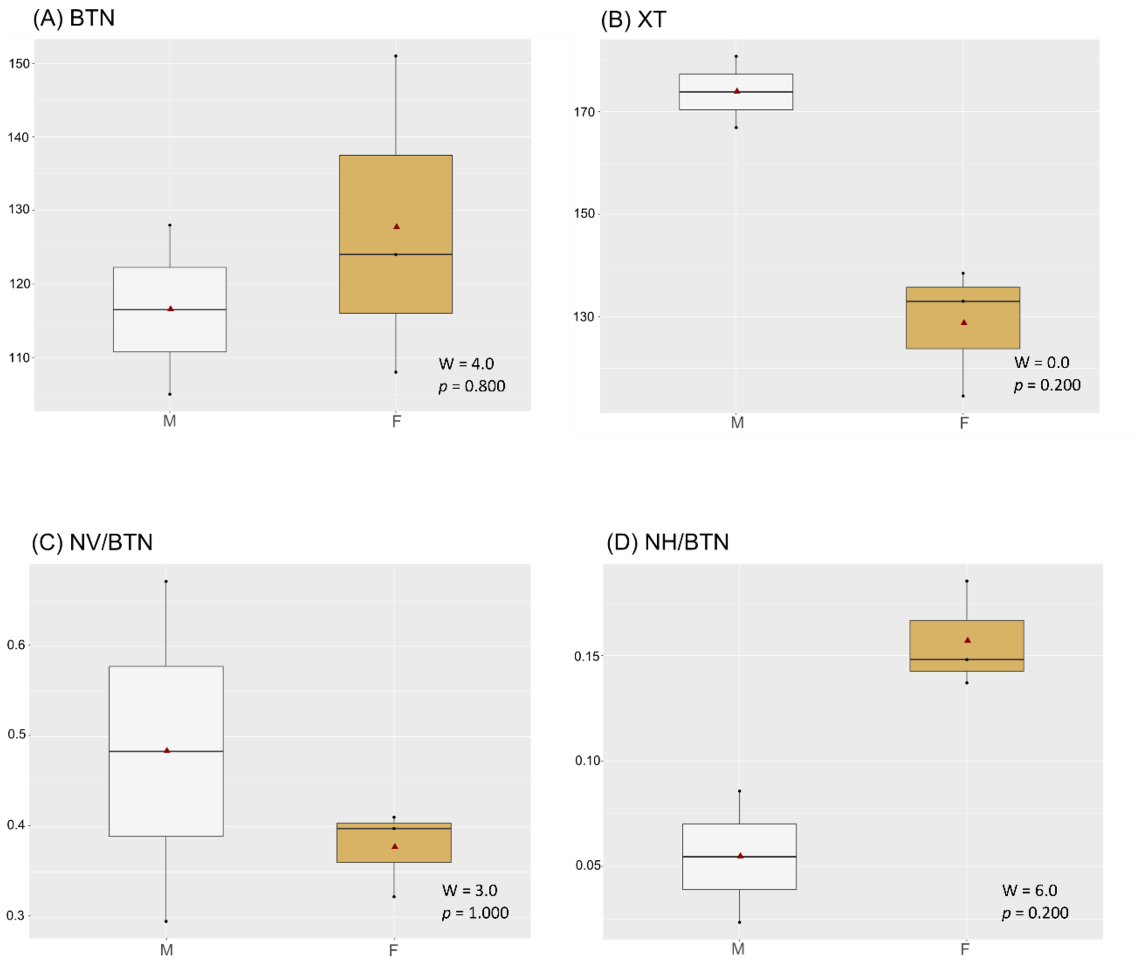

Figure S5: Boxplot showing (a) the total number of striations and (b) pits, (c) area of the pits and (d) percentage of pits on the occlusal surface by period (N = 34). Red triangles show the means, middle horizontal lines represent the medians. Middle Neolithic (MN, n = 16); Middle Copper Age (MCA, n = 10); Late Copper Age (LCA, n = 3); Middle Bronze Age (MBA, n = 4); Late Bronze Age (LBA, n = 1). X^2: Kruskall Wallis test; *p*: p-value.

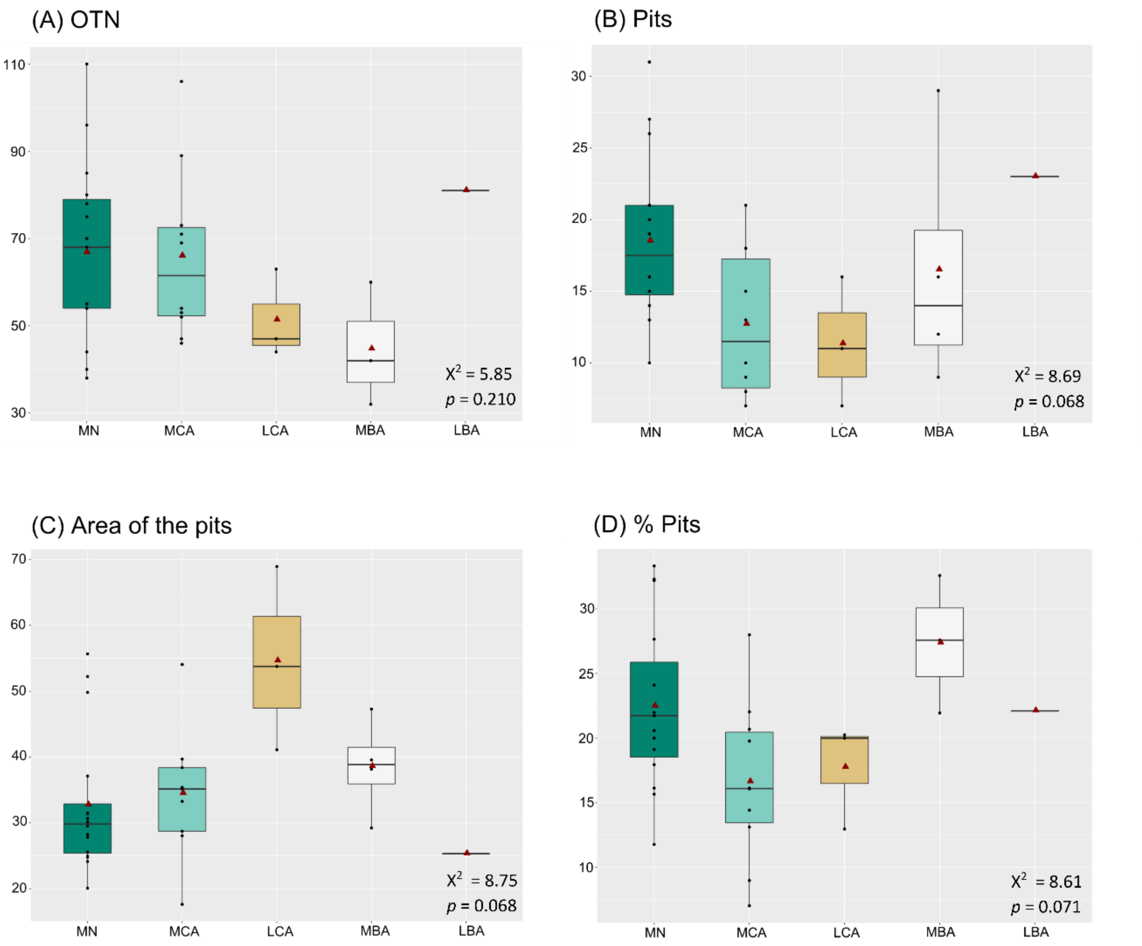

Figure S6: Boxplot showing (a) the total number of striations and (b) pits, (c) area of the pits and (d) percentage of pits on the occlusal surface on the Middle Neolithic (N = 14). Red triangles show the means, middle horizontal lines represent the medians. Sites: Bükkábrány-Bánya VII (BB-VII, n = 4); Bükkábrány-Bánya XI/A (BB-XI/A, n = 7); Bükkábrány-Bánya XII/B (BB-XII/B, n = 3). X^2: Kruskall Wallis test; *p*: p-value.

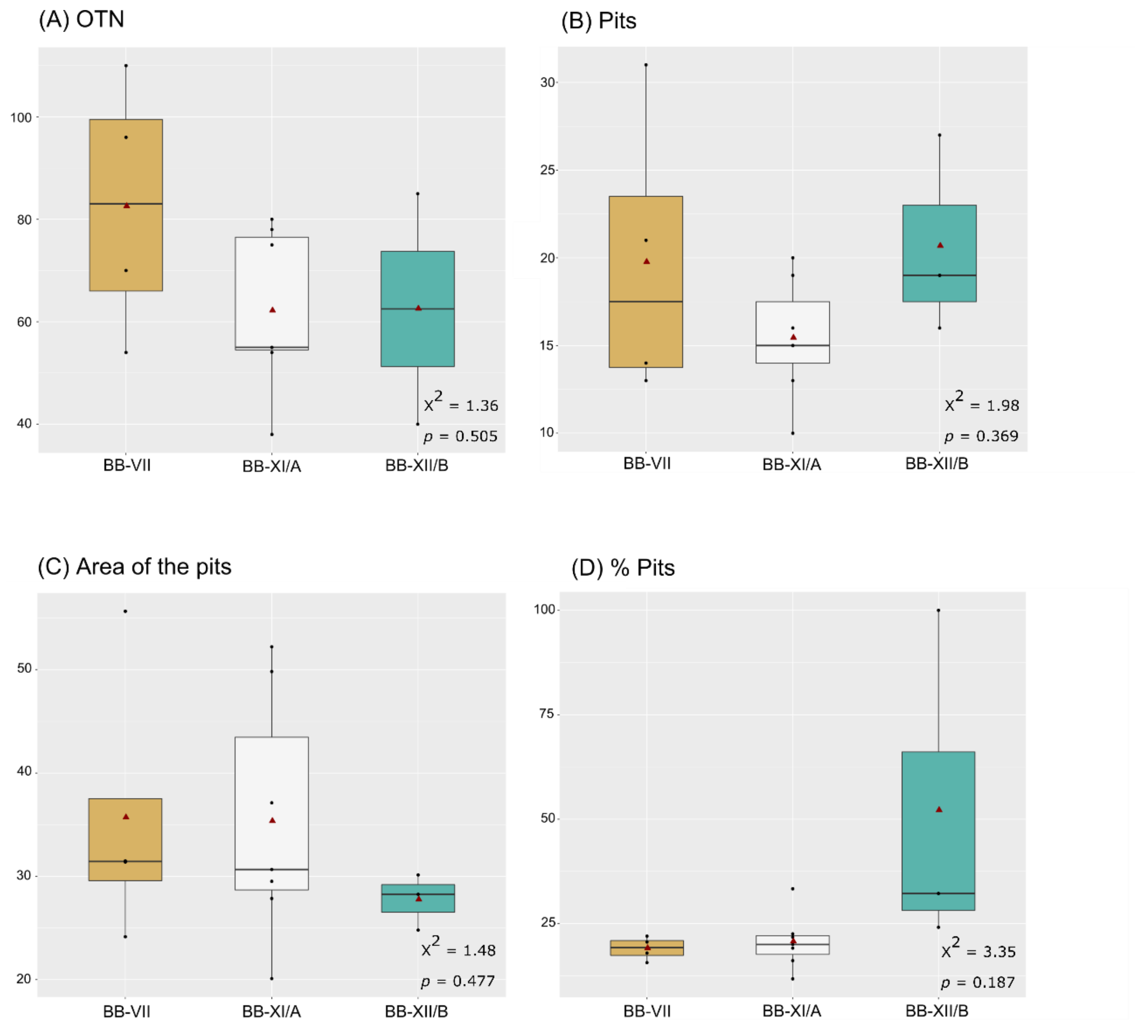

Figure S7: Boxplot showing (a) the total number of striations and (b) pits, (c) area of the pits and (d) percentage of pits on the occlusal surface by sex (males (n = 5): M; females (n = 3): F) in Middle Neolithic individuals. Red triangles show the means, middle horizontal lines represent the medians. W: Mann-Whitney test; *p*: p-value.

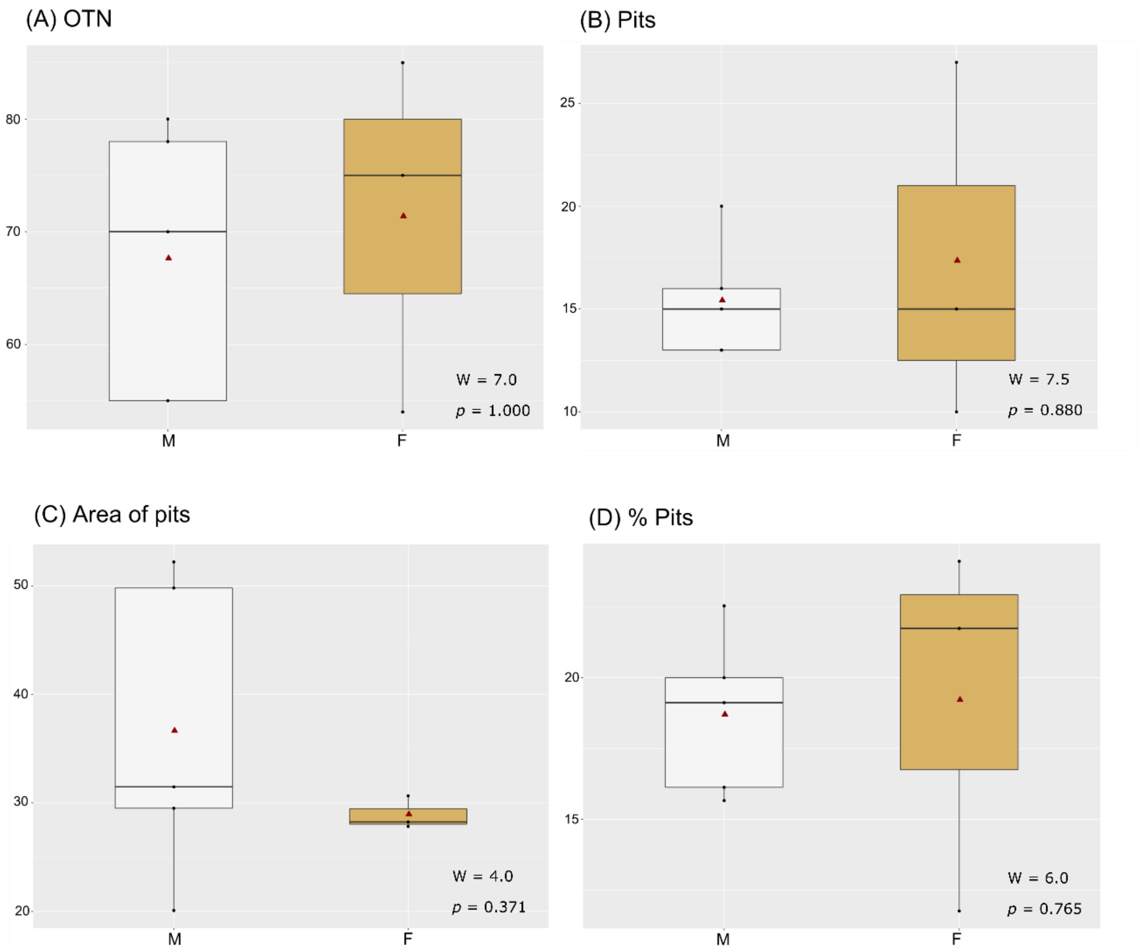

Figure S8: Boxplot showing (a) the total number of striations and (b) pits, (c) area of the pits and (d) percentage of pits on the occlusal surface by sex (males (n = 1): M; females (n = 4): F) in Middle Copper Age individuals. Red triangles show the means, middle horizontal lines represent the medians.

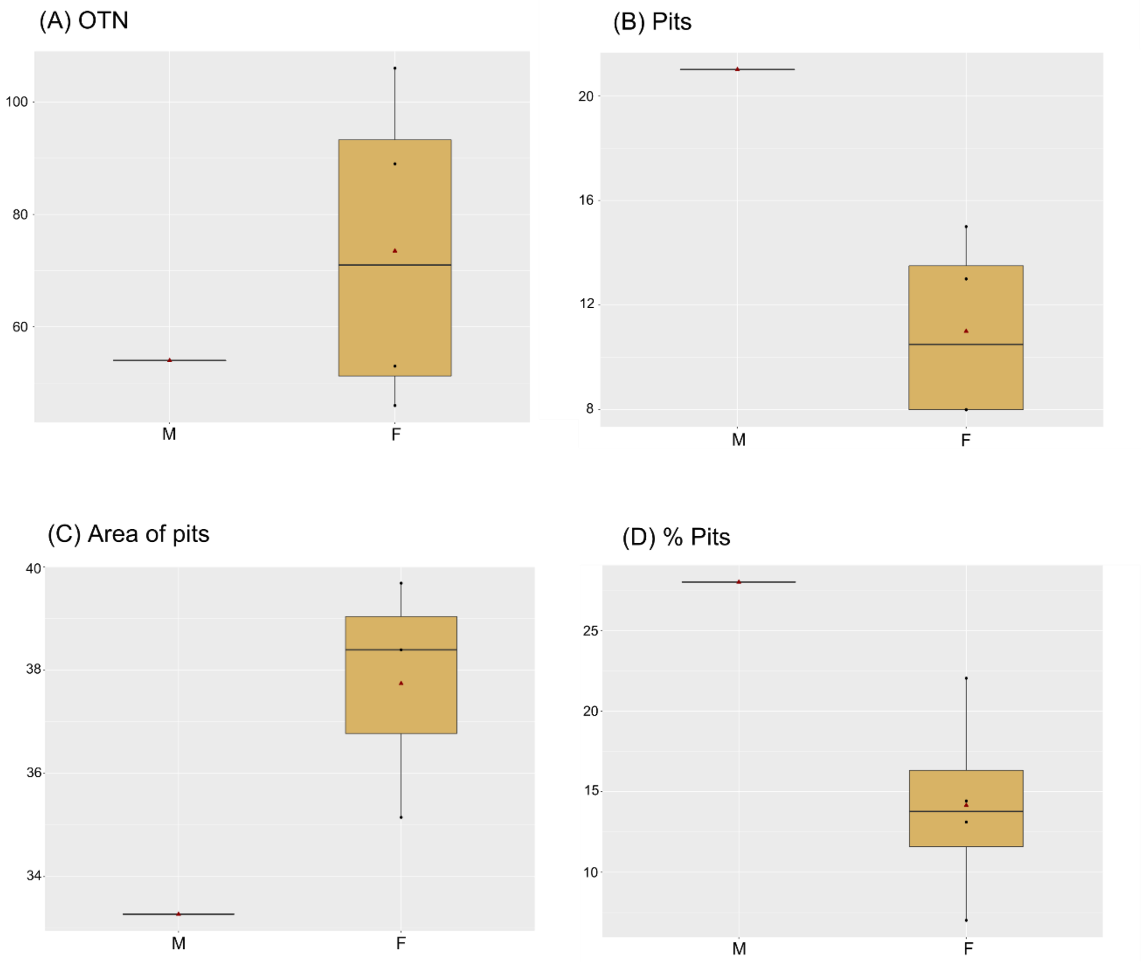

Supplement: Supplementary file 2 — Supplementary Information 2. [file 41598_2021_86369_MOESM2_ESM.pdf]
