# Supplementary material for: Integrating buccal and occlusal dental microwear with isotope analyses for a complete paleodietary reconstruction of Holocene populations from Hungary
Source: Sci Rep. 2021 Mar 29;11:7034. doi: 10.1038/s41598-021-86369-x (PMC8007593; doi:10.1038/s41598-021-86369-x)
Supplement: Supplementary file 3 — Supplementary Information 3. [file 41598_2021_86369_MOESM3_ESM.pdf]

Supplementary Figures S9-S12

Integrating buccal and occlusal dental microwear with isotope analyses for a complete paleodietary reconstruction of Holocene populations from Hungary.

Raquel Hernando[1,2*], Beatriz Gamarra[2,1,3*], Ashley McCall[3], Olivia Cheronet[4,3], Daniel Fernandes[4,5,3], Kendra Sirak[6,7,3], Ryan Schmidt[8,3], Marina Lozano[2,1], Tamás Szeniczey[9,10], Tamás Hajdu[9,10], Annamária Bárány[11], András Kalli[12], Eszter K. Tutkovics[13], Kitti Köhler[14], Krisztián Kiss[9,10], Judit Koós[15], Piroska Csengeri[15], Ágnes Király[14], Antónia Horváth[15], Melinda L. Hajdú[15], Krisztián Tóth[16], Róbert Patay[17], Robin N. M. Feeney[18], Ron Pinhasi[4]

*Corresponding authors: r.hernando90@gmail.com and beagamarra@gmail.com. These authors contributed equally to this work.

[1]Universitat Rovira i Virgili, Departament d'Història i Història de l'Art, Avinguda de Catalunya 35, 43002 Tarragona, Spain.
[2]Institut Català de Paleoecologia Humana i Evolució Social (IPHES), Zona Educacional 4, Campus Sescelades URV (Edifici W3), 43007 Tarragona, Spain.
[3]School of Archaeology and Earth Institute, University College Dublin, Dublin, Ireland.
[4]Department of Evolutionary Anthropology, University of Vienna, Vienna, Austria.
[5]CIAS, Department of Life Sciences, University of Coimbra, 3000-456 Coimbra, Portugal.
[6]Department of Genetics, Harvard Medical School, Boston, MA 02115, USA.
[7]Department of Human Evolutionary Biology, Harvard University, Cambridge, MA 02138, USA
[8]CIBIO-InBIO, Universidade do Porto, Portugal.
[9]Department of Biological Anthropology, Eötvös Loránd University, Budapest, H-1117 Pázmány Péter sétány 1/c.
[10]Department of Anthropology, Hungarian Natural History Museum, Budapest, H-1083, Ludovika tér 2.
[11]Department of Archaeology, Hungarian National Museum, Budapest, H-1088, Múzeum krt. 14-16.
[12]Várkapitányság Integrált Területfejlesztési Központ Nonprofit Zrt., H-1113 Budapest, Daróczi út 3., Hungary.
[13]Rétközi Museum, H-4600 Kisvárda, Csillag u. 5., Hungary.
[14]Institute of Archaeology, Research Centre for the Humanities, Loránd Eötvös Research Network, Budapest, H-1097 Tóth Kálmán utca 4.
[15]Herman Ottó Museum, H- 3529 Miskolc, Görgey Artúr u. 28, Hungary.
[16]Dornyay Béla Museum, H-3100 Salgótarján, Múzeum tér 2., Hungary.
[17]Department of Archaeology, Ferenczy Museum Center, Szentendre, H-2000 Fő tér 2–5.
[18]School of Medicine, University College Dublin, Dublin, Ireland.

Figure S9. Individual stable carbon and nitrogen isotope ratios of human and faunal bone collagen from GHP sites analysed in this study. Human samples include both adults and young individuals. Infant refers to Infant I group; Sub-adults individuals groups Infant II and Juveniles; and Adults include Adults and Mature groups (according to1). Boxes show the expected signature of domesticated fauna samples (ovicaprids, cattle and pigs) in humans's diet (assuming +2-3‰ in nitrogen and +0-1‰ in carbon.

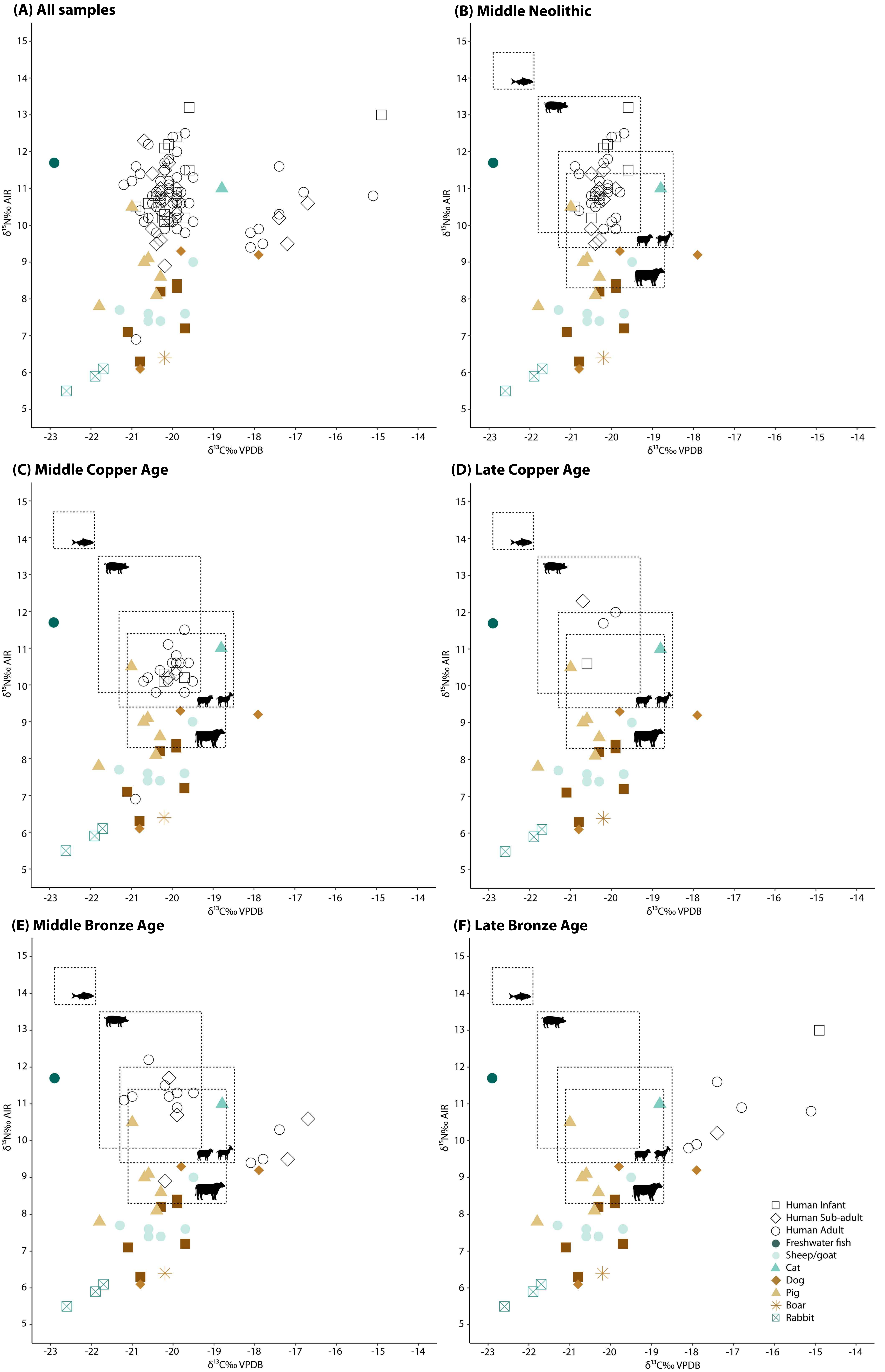

Figure S10: Boxplot showing δ^{13}C and δ^{15}N values for human samples of Middle Copper Age sites (N = 15). Site abbreviations: Bükkábrány-Bánya XI/B (BB-XI/B, n = 11); Mezőkövesd–Patakra járó dűlő (M-P, n = 4). Red triangles show the means, middle horizontal lines represent the medians. W: Mann-Whitney test; *p*: p-values.

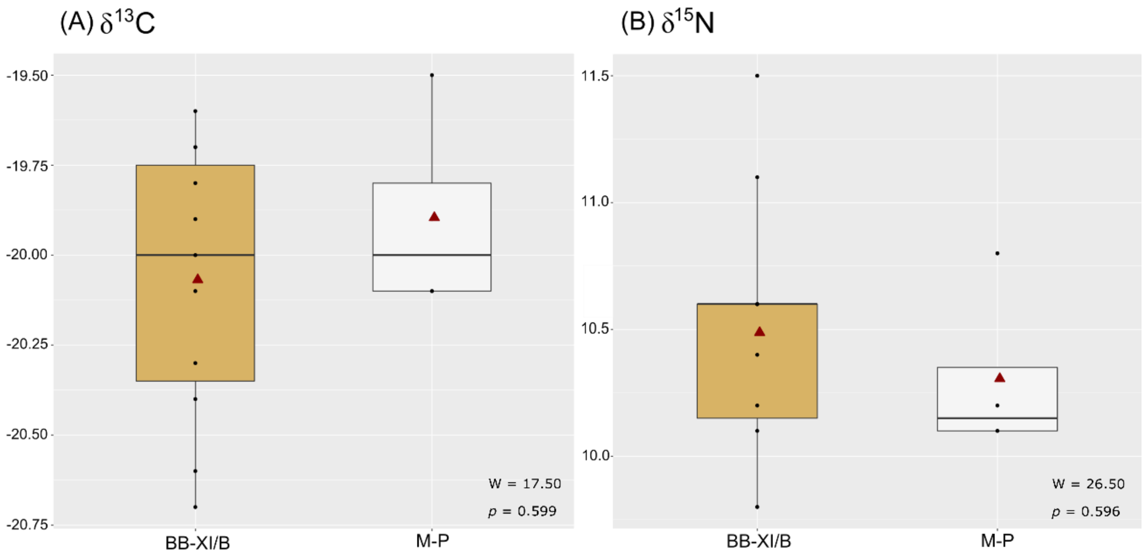

Figure S11: Boxplot showing δ^{13}C and δ^{15}N values for human samples of Middle Bronze Age sites (N = 13). Human isotopic values were previously published in[2]. Site abbreviations: Mezőzombor−Községi temető (M-K, n = 8); Vatta−Dobogó (V-D, n = 5). Red triangles show the means, middle horizontal lines represent the medians. W: Mann-Whitney test; *p*: p-values.

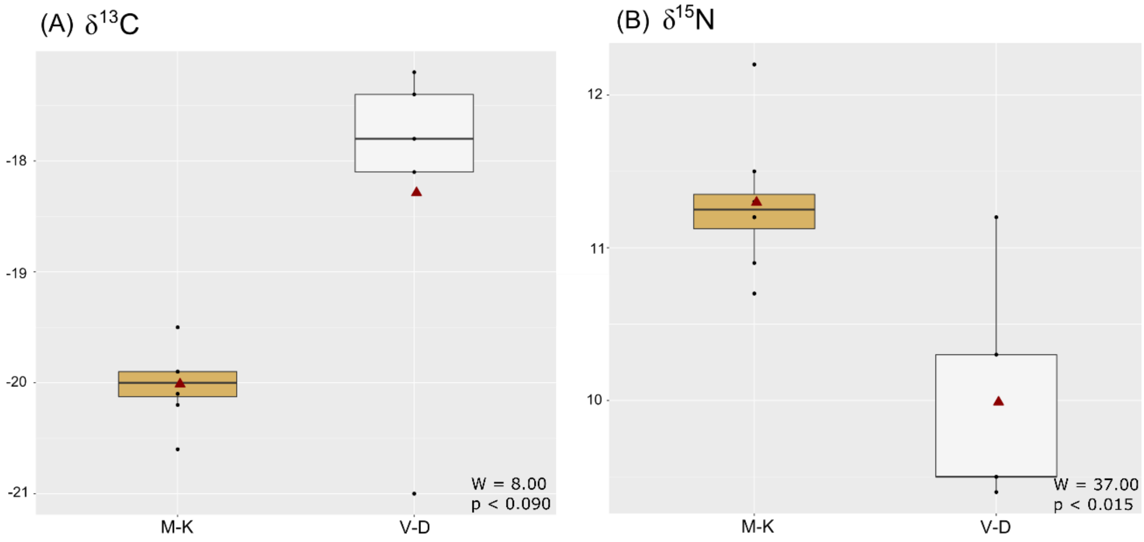

Figure S12: Boxplot showing $\delta^{13}C$ and $\delta^{15}N$ values for human samples by sex (males: M; females: F) for each period. Red triangles show the means, middle horizontal lines represent the medians. Middle Neolithic: M (n = 11), F (n = 7); Middle Copper Age: M (n = 5), F (n = 5); Middle Bronze Age: M (n = 7), F (n = 6).

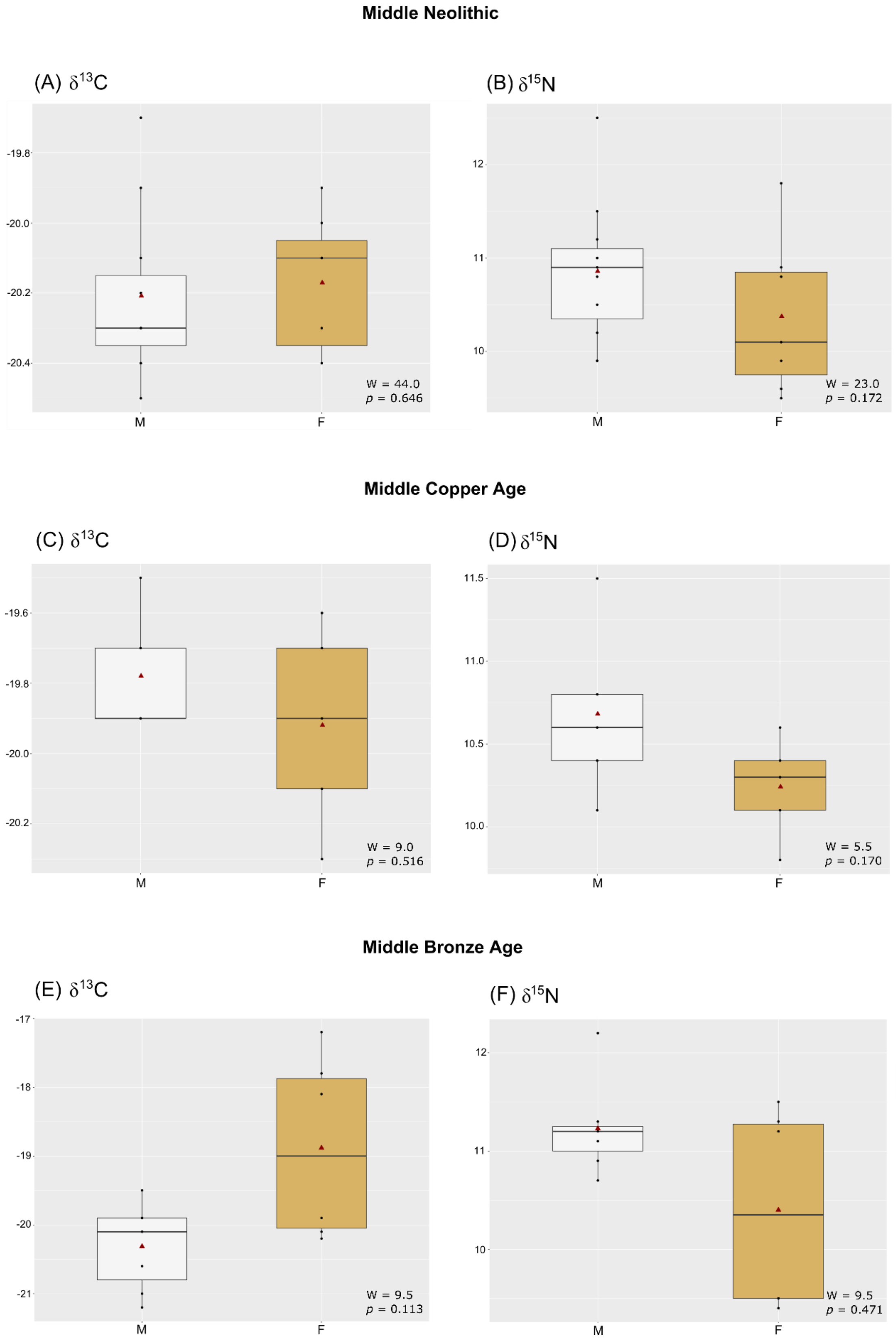

1. Martin, R. & Saller, K. *Lehrbuch der Anthropologie, in systematischer Darstellung*. (Gustav Fischer Verlag, 1957).
2. McCall, A. The Relationship of Stable Isotopes to Great Hungarian Plain Diet and Mobility Through the Neolithic, Copper Age, Bronze Age, and Iron Age. (PhD Dissertation, University College of Dublin, 2020).
