# Supplementary material for: Integrating buccal and occlusal dental microwear with isotope analyses for a complete paleodietary reconstruction of Holocene populations from Hungary
Source: Sci Rep. 2021 Mar 29;11:7034. doi: 10.1038/s41598-021-86369-x (PMC8007593; doi:10.1038/s41598-021-86369-x)
Supplement: Supplementary file 4 — Supplementary Information 4. [file 41598_2021_86369_MOESM4_ESM.pdf]

<u>**Supplementary Information. Archaeological sites descriptions**</u>

Integrating buccal and occlusal dental microwear with isotope analyses for a complete paleodietary reconstruction of Holocene populations from Hungary.

Raquel Hernando[1,2*], Beatriz Gamarra[2,1,3*], Ashley McCall[3], Olivia Cheronet[4,3], Daniel Fernandes[4,5,3], Kendra Sirak[6,7,3], Ryan Schmidt[8,3], Marina Lozano[2,1], Tamás Szeniczey[9,10], Tamás Hajdu[9,10], Annamária Bárány[11], András Kalli[12], Eszter K. Tutkovics[13], Kitti Köhler[14], Krisztián Kiss[9,10], Judit Koós[15], Piroska Csengeri[15], Ágnes Király[14], Antónia Horváth[15], Melinda L. Hajdú[15], Krisztián Tóth[16], Róbert Patay[17], Robin N. M. Feeney[18], Ron Pinhasi[4]

*Corresponding authors: r.hernando90@gmail.com and beagamarra@gmail.com. These authors contributed equally to this work.

[1]Universitat Rovira i Virgili, Departament d'Història i Història de l'Art, Avinguda de Catalunya 35, 43002 Tarragona, Spain.
[2]Institut Català de Paleoecologia Humana i Evolució Social (IPHES), Zona Educacional 4, Campus Sescelades URV (Edifici W3), 43007 Tarragona, Spain.
[3]School of Archaeology and Earth Institute, University College Dublin, Dublin, Ireland.
[4]Department of Evolutionary Anthropology, University of Vienna, Vienna, Austria.
[5]CIAS, Department of Life Sciences, University of Coimbra, 3000-456 Coimbra, Portugal.
[6]Department of Genetics, Harvard Medical School, Boston, MA 02115, USA.
[7]Department of Human Evolutionary Biology, Harvard University, Cambridge, MA 02138, USA
[8]CIBIO-InBIO, Universidade do Porto, Portugal.
[9]Department of Biological Anthropology, Eötvös Loránd University, Budapest, H-1117 Pázmány Péter sétány 1/c.
[10]Department of Anthropology, Hungarian Natural History Museum, Budapest, H-1083, Ludovika tér 2.
[11]Department of Archaeology, Hungarian National Museum, Budapest, H-1088, Múzeum krt. 14-16.
[12]Várkapitányság Integrált Területfejlesztési Központ Nonprofit Zrt., H-1113 Budapest, Daróczi út 3., Hungary.
[13]Rétközi Museum, H-4600 Kisvárda, Csillag u. 5., Hungary.
[14]Institute of Archaeology, Research Centre for the Humanities, Loránd Eötvös Research Network, Budapest, H-1097 Tóth Kálmán utca 4.
[15]Herman Ottó Museum, H- 3529 Miskolc, Görgey Artúr u. 28, Hungary.
[16]Dornyay Béla Museum, H-3100 Salgótarján, Múzeum tér 2., Hungary.
[17]Department of Archaeology, Ferenczy Museum Center, Szentendre, H-2000 Fő tér 2–5.
[18]School of Medicine, University College Dublin, Dublin, Ireland.

Sites description (in alphabetical order)

Arnót-Arnóti-oldal Dél is situated in Borsod-Abaúj-Zemplén county, 2 kms from the eastern part of Miskolc city on the left bank of the river Sajó. The site yielded materials from Neolithic Period (Alföld Linear Pottery culture - ALP), Middle Bronze Age, Late Iron Age (La Téne), Roman Period (Germans) and to Middle Ages. Besides postholes of a house, long pits, a pit using as kiln and a well with wooden lining, 5 graves had been discovered from Middle Neolithic. One grave contained an obsidian blade, and another grave a green slate chisel situated near the head. According to radiocarbon dates of the well, the ALP settlement would be dated to 5211-4964 cal BC[1].

Arnót-Nagy-bugyik is situated in Borsod-Abaúj-Zemplén county, 3 kms from the eastern part of Miskolc city on the floodplain of the river Sajó on a small sandy hill. Large scale rescue excavations were carried out while building M30 connection motorway around Miskolc in 2014-2015. The site yields materials from Neolithic Period (ALP culture), Copper Age (Baden culture), Roman Period (Germans), Early Migration Period, and to Middle Ages. The ALP settlement was dated in 5400 – 5100 cal BC. Long pits and a well with 4 graves were found. In both cases grinding stones had been set close to the the individual's heads.

Bükkábrány – Bánya is situated in North-Hungary, on the territory of Borsod-Abaúj-Zemplén County, 30 km far from Miskolc, between Vatta, Csincse and Bükkábrány villages. The archaeological site is situated geographically at the border of the Great Hungarian Plain and the Bükk Mountains, on the territory of a typical transition zone between plains and moutains, called Borsodi Mezőség. This site is situated close to the valley of the Csincse-brook. In this area large scale rescue excavations were carried out in connection with the operation of Bükkábrány lignite mine. Due to the continuous mining activities, the entire area of the archaeological site is being mined and destroyed along with its natural environment[2]. Therefore, it is not possible to do any on-site examinations or sampling due to the urgent character of the excavation. Nevertheless, this archaeological area has yielded a great amount of interesting archaeological remains, here presented in different sites designations.

Bükkábrány – Bánya VII

The excavation of this site was carried out during 2011-2012. The site yielded materials from the Middle Neolithic ALP and Roman imperial (Sarmatian) period. The ALP occupation is dated to the early phase of the ALP culture and includes 377 Neolithic objects (postholes, extensive clay pits – Längsgrube - with various finds: broken pottery, fauna, stone tools and fragments of wattle and daub, pit complexes, fireplaces and graves). The postholes belonged to long rectangular houses, that were excavated between the clay pits. Eighteen mostly intact graves were excavated from the ALP phase. One half-cuted human skeleton was found in one of clay pit. The settlement was radiocarbon dated around 5480-5210 cal BC.

Bükkábrány – Bánya X

The excavation of this site was carried out during 2015-2016. The site yielded materials from the Middle Neolithic ALP, Late Copper Age, Early/Middle Bronze Age, Late Bronze Age and Iron Age period. The ALP occupation includes 72 Neolithic objects (pits, screes made of mostly wettle and daub, pit complexes, few postholes and three graves). There are no radiocarbon dates from this site.

Bükkábrány – Bánya XIA

The excavation of this site was carried out during 2012-2013. The site yielded materials from the Middle Neolithic (ALP culture), Copper age, Iron age and Roman imperial (Sarmatian) period. The ALP occupation was radiocarbon dated to the early phase of the ALP culture and includes 243 Neolithic objects (postholes, extensive clay pits – Längsgrube - with various finds: broken pottery, fauna, stone tools and fragments of wattle and daub, pit complexes, fireplaces and graves). The postholes belonged to long rectangular houses, that were excavated between the clay pits. Twenty mostly intact graves were excavated from the ALP phase. According to the radiocarbon dates, the settlement dated around 5470-4840 cal BC.

Bükkábrány – Bánya XIB

The excavation of this site was carried out during 2012-2014. The site yielded materials from the Middle Neolithic (ALP culture), Early Copper Age (Tiszapolgár culture), Middle Copper Age (Bodrogkeresztúr and Hunyadihalom cultures), Late Copper Age (Baden culture), Late Iron Age (Celtic/La Tène culture) and Roman imperial (Sarmatian) period. The Middle Copper Age, Bodrogkeresztúr culture includes 39 graves. No radiocarbon dates are available.

Bükkábrány – Bánya XIIA

The excavation of this site was carried out during 2014-2015. The site yielded materials from the Middle Neolithic (ALP culture), Middle Copper Age (Hunyadihalom group), Late Copper Age (Baden culture), Late Iron Age (Celtic/La Tène culture) and Roman imperial (Sarmatian) period. Middle Neolithic ALP human remains were found in a grave. Meanwhile, Late Copper Age human remains came from pits. There are not radiocarbon dates available.

Bükkábrány – Bánya XIIB

The excavation of this site was carried out during 2014-2015. The site yielded materials from the Middle Neolithic, Late Copper Age (Baden culture), Late Iron Age (Celtic/La Tène culture) and Roman imperial (Sarmatian) period. The Middle Neolithic period includes pits, postholes and six graves. The Baden period includes mostly pits, some postholes, fireplace and one well. Middle Neolithic human remains came from graves. Late Copper Age human remains came from pits. There are no radiocarbon dates from these periods are available.

Csincse-Gomba Barna földje (M3- sites 14-16) is situated in the Great Hungarian Plain, south of Borsod-Abaúj-Zemplén County, Northeast Hungary. Rescue excavations were carried out in connection with construction of the M3 motorway at the site, in 1994–1995. The site is situated at both of the banks of the watercourse Csincse. The settlement was first occupied in the Middle Neolithic ALP culture, perhaps in ALP II phase or Early Tiszadob period (no radiocarbon data from the site). Several ALP pits and 6 graves were excavated of which two were selected for analysis in this study. Settlement remains and graves of the Late Copper Age (Baden culture), Early Bronze Age (Makó culture), Roman (Sarmatian) Period also were found on the site[3,4].

Felsődobsza-2 is situated in the Hernád valley, northeast to Miskolc (Borsod-Abaúj-Zemplén county, Northeastern Hungary). In connection with the construction of a flood defense dam, a soil extraction site was established on the southwestern outskirts of the village in 2012. The site covers more than 2 hectares. The excavations (led by Miklós Makoldi) revealed 4 different chronological periods. Besides Middle Neolithic, a Germanic and a Medieval village, and an extensive Late Bronze Age settlement occupied most of the area. More than 150 features were dated to this period(1400/1300 – 900/850 BC), in one of which (S 49), a flexed human corpse (S 62) was found[5].

Köröm-Kápolna-domb is located in the floodplain area of the Sajó stream (Borsod-Abaúj-Zemplén county, Northeastern Hungary. In 1969, 15 features from the Late Bronze Age (Gáva culture, 1200/1100-900/850 BC)) were found during a rescue excavation, conducted by Tibor Kemenczei[6]. Kemenczei mistakenly called the site Rákóczi-domb, that was corrected later by Magdolna B. Hellebrandt[7]. A second rescue excavation took place in 1996. This time, 85 features were associated with the Gáva culture: 5 houses, 20 pits and 11 clay extracting pits, besides 34 burials dated to the Árpádian Era (12-13th century A. D.). Zsolt Gallina conducted a third rescue excavation in 2014. This excavation revealed 69 archaeological features including 39 pits from the Late Bronze Age and seven graves from the Early Medieval times. During the 2014 rescue excavation, two human skeletons were found buried in an abnormal position in the beehive-shaped pits of feature no. SNR 020. The radiocarbon date from a faunal bone remain was dated to 1191 – 944 cal BC (AMS C14 date, calibrated by using Calib 7.0.4, lab code: DeA-11635)[6,7].

Mezőkeresztes-Cethalom (M3- site 10) is situated in the southern part of Borsod-Abaúj-Zemplén county (Northeastern Hungary), 15 kilometres north of Lake Tisza. Due to the construction of M3 motorway, parts of a Late Bronze Age (Gáva culture) settlement was excavated in 1995, in the southeastern vicinities of the town. Feature Nr. 154, a bell-shaped pit, revealed a disturbed skeleton of a child and a dog, as well as a large deer antler. According to the AMS radiocarbon analysis of the samples taken from the human remains, the individual dated between 1379 and 1130 cal BC (calibrated by using Calib 7.0.4, lab code: DeA-11654)[8,9].

Mezőkeresztes -Csincsetanya. The village of Csincse is located in the southern part of Borsod-Abaúj-Zemplén county in north-eastern Hungary, 25 km from Miskolc, in the core area of the Middle Bronze Age Füzesabony culture. In 1968 during the drilling of a hydrologic observation well, in 1 m deep, a skeleton was found in a crouched position. The body was buried with a bowl and two vessels, which suggests that the burial can be dated to the previous advanced, classical period of the Füzesabony culture.

Mezőkövesd-Klementina (Szentistván- airport). The site is located in the southern part of Borsod-Abaúj-Zemplén county, at the confluence of the Great Hungarian Plain and the Bükk

Mountains. In 1993, kerosene removal was carried out on the site of the former Soviet military airport, during which archaeological finds were unearthed. In addition to Neolithic (AVK 1) findings, Copper Age (Tiszapolgár culture, Bodrogkeresztúr culture) and Roman Period objects were found. The finds of the Tiszapolgár culture (Hunyadihalmi group) also contain rare types of gold headdresses.

Mezőkövesd-Patakra járó dűlő. In 2000, at the mining area (No. 1) of Mezőkövesd located in the southern part of Borsod-Abaúj-Zemplén county, archaeological finds and human remains of the Bodrogkeresztúr culture were found. The finds include obsidian arrowheads, stone tools, bone pearls, and fragments of potteries characteristic of the phase ”B” of the Bodrogkeresztúr culture. In addition to the 6 individuals of the Bodrogkeresztúr culture, finds of the Tiszapolgár culture (Hunyadhalmi group), a Bronze Age urn burial and a smaller Roman Period (Sarmatian) cemetery were also found.

Mezőzombor-Községi temető. The village is located in Borsod -Abaúj -Zemplén county, at the confluence of the Great Plain and the mountains, in the Central Tisza region, 41 km east of Miskolc. During grave-digging in the local cemetery, archaeological findings were found, followed by a rescue excavation. Eight skeletons from the Middle Bronze Age (Füzesabony culture) were found in a crouched position. Based on the examination of the findings, the cemetery can be dated to the advanced, classical period of the Füzesabony culture. During the earthworks carried out at the site, secondary findings from different periods were also discovered: Neolithic (AVK. Tiszadob-Bükk culture), Copper Age (Bodrogkeresztúr culture), Early Bronze Age (Nyírség culture), Late Bronze Age (Kyjatice culture) and mainly Roman Period (Sarmatian).

Nagyrozvágy-Pap-domb is located on the northeastern outskirts of Borsod-Abaúj-Zemplén county, next to the Slovak border. Construction of an emergency reservoir was started nearby, as the area is a floodplain next to the Bodrog River. In this connection, a large-scale excavation was started in 2005-2007, as a result of which the settlement of the hitherto unique, late, bodrogszerdahelyi (Streda nad Bodrogom) phase of the Middle Bronze Age Füzesabony culture was discovered. Structural elements referring to houses, fountains, gold and bronze objects, as well as objects of everyday use were found. In addition to the three individuals buried within the

settlement, dispersal human remains were also found. The radiocarbon data from the settlement dated between 1740-1440 cal BC.

Oszlár-Nyárfaszög (M3-32 site) is located in the southern part of Borsod-Abaúj-Zemplén county, on the southeastern border of the village of Oszlár, on the northern edge of the Great Plain, in the area bordered by the rivers Hejő and Tisza. In 1996-97, large-scale excavations were carried out due to the construction work on the M3 motorway, which proved to be one of the largest Late Bronze Age excavations in recent decades. The settlement, which has been inhabited for a long time, is connected to the Carpathian Tumulus culture, the Piliny culture, and the pre - Gáva period (RB BD - HA1). In addition to the burial pits from the Late Bronze Age, early Bronze Age cremation burials (Makó culture) and Roman Period (Sarmatian) inhumation graves were also excavated.

Pácin-Alsókenderszer.Pácin is a village at the northeastern borders of Hungary (Borsod-Abaúj-Zemplén county). During the preventive archeological excavations related to the construction of a floodplain reservoir, a part of a Late Bronze Age settlement was unearthed in 2005-2006. The excavation was conducted by Emese Lovász. Some urn graves and two settlement features (S 64 and S 100) contained human skeletons. According to the AMS radiocarbon analysis of the samples taken from one of the human remains in feature Nr. 64, the individual could be dated to the period between 1192-977 cal BC (calibrated by using Calib 7.0.4, lab code: DeA-11753).

Rásonysápberencs-Szőlő alja is situated in the Cserehát Hills, 30 km northeast from Miskolc, in Borsod-Abaúj-Zemplén County, Northeast Hungary. Rescue excavation was carried out while constructing a reservoir against floods at the site, in 2014. The site is situated at both of the banks of the watercourse Vasonca. The settlement was first occupied in the late phase of the Middle Neolithic ALP culture, in the Tiszadob-Bükk periods, between 5,300–5,000 cal BC. Some ALP houses, several pits and 7 graves were excavated of which three were selected for analyses in this study (including S379 with post construction). Settlement remains of the Early and Late Bronze Age, Iron Age, Roman (Germanic), Avarian and Early Medieval Periods also were yielded at the site[10].

Vatta-Dobogó. The village of Vatta is located in Borsod-Abaúj-Zemplén county, at the confluence of the Great Hungarian Plain and the mountains. The site is located south of the village, next to the Bronze Age tell settlement of Vatta-Testhalom, in the area of the Bükkábrány lignite mine, wherein 2009-2010 water management tasks and stream bed relocations were carried out. During the preventive excavation works, the outer settlement and the protective rampart of the early Bronze Age Hatvan culture and the Middle Bronze Age Füzesabony culture were found. In addition to the settlement, Neolithic, Early Bronze Age and Middle Bronze Age burials have also been unearthed. In this site, objects from the Late Bronze Age Piliny culture and the Roman Period Sarmatians were also found.

References:

1. Csengeri, P. A. Herman Ottó Múzeum régészeti kutatásai 2014-ben. *A Herman Ottó Múzeum Évkönyve* **55**, 141–185 (2016).
2. Kalli, A. & Tutkovics, E. K. Archaeological Research on the Area of the Bükkábrány Lignite Mine in the Valley of the Csincse Stream. *Hungarian Archaeoogy. E-Journal* **Spring**, 1–11 (2017).
3. Wolf, M. Csincse-Gomba Barna földje (M3-as autópálya, 14. lh.) in *év régészeti kutatásai* (ed. Wollák, K.) 61–62 (1995).
4. Zimborán, G. Újkőkori leletek Csincse 14. számú lelőhelyéről. (Unpublished MA Thesis, Eötvös Loránd University, 2002).
5. Makoldi, M. Felsődobsza 2. lelőhely. Régészeti Kutatások Magyarországon in *Archaeological Investigations in Hungary* (eds. Kvassay, J. & Kreiter, A.) (Hungarian National Museum, 2012).
6. Kemenczei, T. *Die Spätbronzezeit Nordostungarns: Archaeologia Hungarica*. (Series Nova L I, Akadémiai Kiado, 1984).
7. Hellebrandt, M. B. A settlement of the Gáva Culture on Köröm-Kápolna-domb in *Yearbook of the Herman Ottó Museum* ,11–124 (2016).
8. Wolf, M. & Simonyi, E. : Előzetes jelentés az M3-as autópálya 10. lelőhelyének

feltárásáról. *Somogyi Múzeumok Közleményei* **11**, 5–32 (1995).

9. Wolf, M. & Simonyi, E. Mezőkeresztes-Cethalom: Kora népvándorláskori temető az V–VI. századból / Gepidic cemetery at Mezőkeresztes-Cethalom. in *Path into the Past* (eds. Raczky, P., Kovács, T. & Anders, A.) 128–132 (1997).

10. Horváth, A., Csengeri, P. & László-Hajdú, M. Rásonysápberencs-Szőlő alja in *A Herman Ottó Múzeum régészeti kutatásai 2014-ben, A Herman Ottó Múzeum Évkönyve LVI* (ed. Csengeri, P.) 168–171 (2016).
