# Supplementary material for: Integrating buccal and occlusal dental microwear with isotope analyses for a complete paleodietary reconstruction of Holocene populations from Hungary
Source: Sci Rep. 2021 Mar 29;11:7034. doi: 10.1038/s41598-021-86369-x (PMC8007593; doi:10.1038/s41598-021-86369-x)
Supplement: Supplementary file 5 — Supplementary Information 5. [file 41598_2021_86369_MOESM5_ESM.pdf]

Supplementary Information Methods

Integrating buccal and occlusal dental microwear with isotope analyses for a complete paleodietary reconstruction of Holocene populations from Hungary.

Raquel Hernando[1,2*], Beatriz Gamarra[2,1,3*], Ashley McCall[3], Olivia Cheronet[4,3], Daniel Fernandes[4,5,3], Kendra Sirak[6,7,3], Ryan Schmidt[8,3], Marina Lozano[2,1], Tamás Szeniczey[9,10], Tamás Hajdu[9,10], Annamária Bárány[11], András Kalli[12], Eszter K. Tutkovics[13], Kitti Köhler[14], Krisztián Kiss[9,10], Judit Koós[15], Piroska Csengeri[15], Ágnes Király[14], Antónia Horváth[15], Melinda L. Hajdú[15], Krisztián Tóth[16], Róbert Patay[17], Robin N. M. Feeney[18], Ron Pinhasi[4]

*Corresponding authors: r.hernando90@gmail.com and beagamarra@gmail.com. These authors contributed equally to this work.

[1]Universitat Rovira i Virgili, Departament d'Història i Història de l'Art, Avinguda de Catalunya 35, 43002 Tarragona, Spain.
[2]Institut Català de Paleoecologia Humana i Evolució Social (IPHES), Zona Educacional 4, Campus Sescelades URV (Edifici W3), 43007 Tarragona, Spain.
[3]School of Archaeology and Earth Institute, University College Dublin, Dublin, Ireland.
[4]Department of Evolutionary Anthropology, University of Vienna, Vienna, Austria.
[5]CIAS, Department of Life Sciences, University of Coimbra, 3000-456 Coimbra, Portugal.
[6]Department of Genetics, Harvard Medical School, Boston, MA 02115, USA.
[7]Department of Human Evolutionary Biology, Harvard University, Cambridge, MA 02138, USA
[8]CIBIO-InBIO, Universidade do Porto, Portugal.
[9]Department of Biological Anthropology, Eötvös Loránd University, Budapest, H-1117 Pázmány Péter sétány 1/c.
[10]Department of Anthropology, Hungarian Natural History Museum, Budapest, H-1083, Ludovika tér 2.
[11]Department of Archaeology, Hungarian National Museum, Budapest, H-1088, Múzeum krt. 14-16.
[12]Várkapitányság Integrált Területfejlesztési Központ Nonprofit Zrt., H-1113 Budapest, Daróczi út 3., Hungary.
[13]Rétközi Museum, H-4600 Kisvárda, Csillag u. 5., Hungary.
[14]Institute of Archaeology, Research Centre for the Humanities, Loránd Eötvös Research Network, Budapest, H-1097 Tóth Kálmán utca 4.
[15]Herman Ottó Museum, H- 3529 Miskolc, Görgey Artúr u. 28, Hungary.
[16]Dornyay Béla Museum, H-3100 Salgótarján, Múzeum tér 2., Hungary.
[17]Department of Archaeology, Ferenczy Museum Center, Szentendre, H-2000 Fő tér 2–5.
[18]School of Medicine, University College Dublin, Dublin, Ireland.

Sex and age-at-death estimation

Our physical anthropological analysis followed the standards set out in human skeletal remains: excavation, analysis, interpretation written by[1]. Sex determination of adult individuals was performed with the examination of sexual dimorphism expressed in the case of 21 morphological traits (Tuber frontale and parietale, glabella and arcus superciliaris, processus mastoideus, protuberantia occipitalis externa, occipital plane, margo supraorbitalis, arcus zygomaticus, corpus mandibulae, mentum, angulus mandibulae and gonion, caput mandibulae, pelvis major, pelvis minor, angulus subpubicus, foramen obturatum, incisura ischiadica major, sacrum, caput femoris, linea aspera, sulcus praeauricularis) with the method implied by[2]. Each of the examinable traits were scored on a scale (-2, -1, 0, +1, +2), where "-2" means hiperfemininity and "+2" means hipermasculinity. After evaluating the scores, a sex for the given individual was determined. The morphological traits of distal humerus[3] and mandibular ramus flexure[4] were also considered. Age-at-death of subadults was estimated based on the development of dentition[5], the metric traits of long bones[6,7] (3,4) and the ossification of apo- and epiphyseal joints[8,9]. For estimating age at death for adults we investigated the morphological traits of pubic symphysis[10], sternal end of ribs[11,12], auricular surface of the ilium[13], and the closure of ectocranial sutures[14]. Finally, age groups were defined according to[15]: Infant I (1-6 years), Infants II (7- 14 years), Juvenile (15-19) and Adult (20-39) and Mature (40-59). However, for statistical purposes, Adult and Mature defined samples were grouped together as adults.

Extraction, library and sequencing methods for Neolithic and Copper Age samples

DNA was extracted from bone powder produced from the cochlear part of the petrous bone following the protocol described by[16], but adapted for the use of High Pure Nucleic Acid Large Volume columns (Roche) instead of the Zymo-Spin V column (Zymo Research) MinElute silica spin column (Qiagen) combination.

Libraries for next-generation sequencing (NGS) were built with all the DNA extracts using a modified version of[17], and amplified through indexing PCR using Accuprime's Pfx Supermix (Life Technology). Negative controls were carried through all steps of extraction, library preparation and indexing. PCR reactions and samples concentrations were assessed on the Agilent 2100 Bioanalyzer following the guidelines of the manufacturer. Based on these concentrations, samples were pooled in equimolar ratios and sequenced on the Illumina NextSeq platform at the UCD Conway Institute of

Biomolecular and Biomedical Research. The samples' sequencing data was processed according to ancient DNA standards. cutadapt v1.12[18] was used to remove sequencing adapters from the biological DNA with the settings -o 1 (minimum overlap) and -m 17 (minimum read length of 17bp), and then aligned those reads to the human reference genome (hg19) using bwa v0.7.5a-r405[19], with disabled seed (-l 1000) and minimum quality score of 30. With samtools v0.1.19-96b5f2294a[20] our reads were restricted to a quality score of above 30 and removed duplicated sequences produced during PCR (polymerase chain reaction). For data authenticity deamination ratios were assessed at the ends of the aligned reads using mapDamage2[21], and estimated contamination using three methods - normalized numbers of reads in blanks[22], mitochondrial mismatches to the consensus sequence[23], and heterozygosity of the X chromosome in male individuals[24]. Samples "passed" by considering a minimum threshold of 5% deamination, maximum contamination of 4% for contamination estimates with a minimum of 10 sites analysed, and finally with a minimum of half of the contamination tests passed (i.e. 1/2, 2/2, 2/3 and 3/3 passed).

References:

1. Ubelaker, D. H. & Buikstra, J. E. *Standards for data collection from human skeletal remains*: *proceedings of a seminar at the Field Museum of Natural History.* (Arkansas Archaeological Survey research series vol. 44, 1994).
2. Éry, K., Kralovánszky, A. & Nemeskéri, J. Történeti népességek rekonstrukciójának reprezentációja (A representative reconstruction of historic population). *Anthropológiai Közlemények* **7,** 41– 90 (1963).
3. Rogers, T. L. A visual method of determining the sex of skeletal remains using the distal humerus. *J. Forensic Sci.* **44**, 57–60 (1999).
4. Loth, S. R. & Henneberg, M. Mandibular ramus flexure: a new morphologic indicator of sexual dimorphism in the human skeleton. *Am. J. Phys. Anthropol.* **99**, 473–485 (1996).
5. AlQahtani, S. J., Hector, M. P. & Liversidge, H. M. Brief communication: The London atlas of human tooth development and eruption. *Am. J. Phys. Anthropol.* **142**, 481–490 (2010).

6. Stloukal, M. & Hanáková, H. Die länge der Längsknochen altslawischer Bevölkerungen unter besonderer Berücksichtigung von Wachstumsfragen. *Homo* **29**, 53–69 (1978).

7. Bernert, Z., Évinger, S. & Hajdu, T. New data on the biological age estimation of children using bone measurements based on historical populations from the Carpathian Basin. *Annales historico-naturales Musei nationalis hungarici* **99**, 199–206 (2007).

8. Schinz, H. . & Case, J. *Roentgen-diagnostics*. (Grune & Stratton, 1952).

9. Ferembach, D., Schwidetzky, I. & Stloukal, M. Empfehlungen für die Alters-und Geschlechtsdiagnose am Skelett. *Homo Gottingen* **30**, 1–32 (1979).

10. Brooks, S. & Suchey, J. M. Skeletal age determination based on the os pubis: a comparison of the Acsádi-Nemeskéri and Suchey-Brooks methods. *Hum. Evol.* **5**, 227–238 (1990).

11. Işcan, M. Y., Loth, S. R. & Wright, R. K. Age estimation from the rib by phase analysis: white males. *J. Forensic Sci.* **29**, 1094–1104 (1984).

12. İşcan, M. Y., Loth, S. R. & Wright, R. K. Age estimation from the rib by phase analysis: white females. *J. Forensic Sci.* **30**, 853–863 (1985).

13. Lovejoy, C. O., Meindl, R. S., Pryzbeck, T. R. & Mensforth, R. P. Chronological metamorphosis of the auricular surface of the ilium: a new method for the determination of adult skeletal age at death. *Am. J. Phys. Anthropol.* **68**, 15–28 (1985).

14. Meindl, R. S. & Lovejoy, C. O. Ectocranial suture closure: a revised method for the determination of skeletal age at death based on the lateral-anterior sutures. *Am. J. Phys. Anthropol.* **68**, 57–66 (1985).

15. Martin, R. & Saller, K. *Lehrbuch der Anthropologie, in systematischer Darstellung*. (Gustav Fischer Verlag, 1957).

16. Dabney, J. *et al.* Complete mitochondrial genome sequence of a Middle Pleistocene cave bear reconstructed from ultrashort DNA fragments. *Proc. Natl. Acad. Sci.* **110**, 15758–15763 (2013).

17. Meyer, M. & Kircher, M. Illumina sequencing library preparation for highly

multiplexed target capture and sequencing. *Cold Spring Harbor Protocols* **2010.6**, pdb-prot5448 (2010).

18. Martin, M. Cutadapt removes adapter sequences from high-throughput sequencing reads. *EMBnet journal* **17**, 10–12 (2011).

19. Li, H. & Durbin, R. Fast and accurate long-read alignment with Burrows–Wheeler transform. *Bioinformatics* **26**, 589–595 (2010).

20. Li, H. *et al.* The sequence alignment/map format and SAMtools. *Bioinformatics* **25**, 2078–2079 (2009).

21. Jónsson, H., Ginolhac, A., Schubert, M., Johnson, P. L. F. & Orlando, L. mapDamage2. 0: fast approximate Bayesian estimates of ancient DNA damage parameters. *Bioinformatics* **29**, 1682–1684 (2013).

22. Gamba, C. *et al.* Genome flux and stasis in a five millennium transect of European prehistory. *Nat. Commun.* **5**, 5257 (2014).

23. Krause, J. *et al.* The complete mitochondrial DNA genome of an unknown hominin from southern Siberia. *Nature* **464**, 894–897 (2010).

24. Korneliussen, T. S., Albrechtsen, A. & Nielsen, R. ANGSD: analysis of next generation sequencing data. *BMC Bioinformatics* **15**, 356 (2014).
