# Supplementary material for: Integrating buccal and occlusal dental microwear with isotope analyses for a complete paleodietary reconstruction of Holocene populations from Hungary
Source: Sci Rep. 2021 Mar 29;11:7034. doi: 10.1038/s41598-021-86369-x (PMC8007593; doi:10.1038/s41598-021-86369-x)
Supplement: Supplementary file 6 — Supplementary Information 6. [file 41598_2021_86369_MOESM6_ESM.pdf]

Supplementary Results: Statistical results of microwear variables. Tables S1-S8

Integrating buccal and occlusal dental microwear with isotope analyses for a complete paleodietary reconstruction of Holocene populations from Hungary.

Raquel Hernando[1,2*], Beatriz Gamarra[2,1,3*], Ashley McCall[3], Olivia Cheronet[4,3], Daniel Fernandes[4,5,3], Kendra Sirak[6,7,3], Ryan Schmidt[8,3], Marina Lozano[2,1], Tamás Szeniczey[9,10], Tamás Hajdu[9,10], Annamária Bárány[11], András Kalli[12], Eszter K. Tutkovics[13], Kitti Köhler[14], Krisztián Kiss[9,10], Judit Koós[15], Piroska Csengeri[15], Ágnes Király[14], Antónia Horváth[15], Melinda L. Hajdú[15], Krisztián Tóth[16], Róbert Patay[17], Robin N. M. Feeney[18], Ron Pinhasi[4]

*Corresponding authors: r.hernando90@gmail.com and beagamarra@gmail.com. These authors contributed equally to this work.

[1]Universitat Rovira i Virgili, Departament d'Història i Història de l'Art, Avinguda de Catalunya 35, 43002 Tarragona, Spain.
[2]Institut Català de Paleoecologia Humana i Evolució Social (IPHES), Zona Educacional 4, Campus Sescelades URV (Edifici W3), 43007 Tarragona, Spain.
[3]School of Archaeology and Earth Institute, University College Dublin, Dublin, Ireland.
[4]Department of Evolutionary Anthropology, University of Vienna, Vienna, Austria.
[5]CIAS, Department of Life Sciences, University of Coimbra, 3000-456 Coimbra, Portugal.
[6]Department of Genetics, Harvard Medical School, Boston, MA 02115, USA.
[7]Department of Human Evolutionary Biology, Harvard University, Cambridge, MA 02138, USA
[8]CIBIO-InBIO, Universidade do Porto, Portugal.
[9]Department of Biological Anthropology, Eötvös Loránd University, Budapest, H-1117 Pázmány Péter sétány 1/c.
[10]Department of Anthropology, Hungarian Natural History Museum, Budapest, H-1083, Ludovika tér 2.
[11]Department of Archaeology, Hungarian National Museum, Budapest, H-1088, Múzeum krt. 14-16.
[12]Várkapitányság Integrált Területfejlesztési Központ Nonprofit Zrt., H-1113 Budapest, Daróczi út 3., Hungary.
[13]Rétközi Museum, H-4600 Kisvárda, Csillag u. 5., Hungary.
[14]Institute of Archaeology, Research Centre for the Humanities, Loránd Eötvös Research Network, Budapest, H-1097 Tóth Kálmán utca 4.
[15]Herman Ottó Museum, H- 3529 Miskolc, Görgey Artúr u. 28, Hungary.
[16]Dornyay Béla Museum, H-3100 Salgótarján, Múzeum tér 2., Hungary.
[17]Department of Archaeology, Ferenczy Museum Center, Szentendre, H-2000 Fő tér 2–5.
[18]School of Medicine, University College Dublin, Dublin, Ireland.

Abbreviations: Middle Neolithic (MN); Late Neolithic (LN); Middle Copper Age (MCA); Late Copper Age (LCA); Middle Bronze Age (MBA); Late Bronze Age (LBA).

MN site abbreviations: Bükkábrány-Bánya VII (BB-VII); Bükkábrány-Bánya XI/A (BB-XI/A); Bükkábrány-Bánya XII/B (BB-XII/B).

Buccal and occlusal abbreviations: Number of striations on the buccal surface (BTN); Length of striations in µm (XT); Vertical Index (NV/BTN); Horizontal Index (NH/BTN); Total number of striations in the occlusal surface (OTN); Percentage of pits (%Pits).

Table S1. Dataset by individual for buccal surface. Abbreviations for tooth types correspond to jaw (upper or lower: U or L), side (left or right: L or R), and position in tooth row (first or second: 1 or 2) for each molar (M). Age in years; Age range category: Infant I (1-6 years), Infant II (7-14 years), Juvenile (15-19 years), Adult (20-39), and Mature (40-59)[1]; in bold the age category used for statistical analyses when the sample age (years) range include two age categories*. Adult diet[2]: see text for explanation. Gender (F= Female, M= Male, NA= No Data); SD: Standard deviation. Variables depend on the orientation (MD: mesio-distal; V: vertical; H: horizontal; DM: disto-mesial).

ID	**Site**	**Period**	**Age**	**Age range category**	**Adult diet**	**Gender**	**Teeth**	**BTN**	**NDM**	**NH**	**NMD**	**NV**	**XT**	**SD**	**NV/BTN**	**NH/BTN**
HUNG871	Bükkábrány-Bánya VII	MN	7-8	Infant II	no	NA	LRM1	77	6	5	14	52	134.05	127.25	0.68	0.06
HUNG872	Bükkábrány-Bánya VII	MN	15-20	Juvenile	yes	M	LLM1	108	12	16	18	62	116.74	77.19	0.57	0.15
HUNG873	Bükkábrány-Bánya VII	MN	15-25	Juvenile-**Adult**	yes	NA	LRM1	129	8	14	60	47	121.77	94.24	0.36	0.11
HUNG877	Bükkábrány-Bánya VII	MN	20-30	Adult	yes	NA	LRM1	109	19	7	31	52	130.26	113.72	0.48	0.06
HUNG878	Bükkábrány-Bánya VII	MN	20-40	Adult	yes	NA	LRM1	101	1	11	44	45	116.58	94.12	0.45	0.11
HUNG880	Bükkábrány-Bánya X	MN	Adult	Adult	yes	NA	LRM2	120	4	16	63	37	129.62	-	0.31	0.13
HUNG882	Bükkábrány-Bánya XI/A	MN	1-6	Infant I	no	NA	LRM1	92	16	9	25	42	115.38	74.70	0.46	0.10
HUNG884	Bükkábrány-Bánya XI/A	MN	30-40	Adult	yes	M	ULM1	93	13	2	26	52	106.24	65.53	0.56	0.02
HUNG885	Bükkábrány-Bánya XI/A	MN	35-45	Adult-Mature	yes	F	URM1	74	20	6	15	33	115.27	-	0.45	0.08
HUNG888	Bükkábrány-Bánya XI/A	MN	11-13	Infant II	yes	NA	LRM1	70	20	9	12	29	135.07	83.83	0.41	0.13
HUNG889	Bükkábrány-Bánya XI/A	MN	20-30	Adult	yes	M	LRM2	79	8	8	29	34	96.48	75.18	0.43	0.10

HUNG893	Bükkábrány-Bánya XI/A	MN	11-12	Infant II	yes	M	LLM1	99	14	1	18	66	117.41	97.47	0.67	0.01
HUNG894	Bükkábrány-Bánya XI/A	MN	25-30	Adult	yes	F	LRM2	112	12	4	27	69	92.23	57.87	0.62	0.04
HUNG895	Bükkábrány-Bánya XI/A	MN	Adult	Adult	yes	M	LRM1	85	8	6	31	40	144.15	119.82	0.47	0.07
HUNG913	Bükkábrány-Bánya XII/A	MN	9-12	Infant II	yes	NA	LLM1	103	6	10	27	60	127.33	94.52	0.58	0.10
HUNG920	Bükkábrány-Bánya XII/B	MN	Adult	Adult	yes	NA	LRM1	91	26	11	24	28	92.29	61.04	0.31	0.12
HUNG922	Bükkábrány-Bánya XII/B	MN	Adult	Adult	yes	NA	LRM1	70	15	15	20	20	120.95	86.36	0.29	0.21
HUNG924	Bükkábrány-Bánya XII/B	MN	Adult	Adult	yes	F	URM2	78	41	20	14	3	94.14	62.99	0.04	0.26
HUNG896	Bükkábrány-Bánya XI/B	MCA	Adult	Adult	yes	NA	LRM2	119	45	8	25	41	118.29	72.15	0.34	0.07
HUNG897	Bükkábrány-Bánya XI/B	MCA	Adult	Adult	yes	M	LLM1	128	15	3	24	86	180.78	105.77	0.67	0.02
HUNG898	Bükkábrány-Bánya XI/B	MCA	Adult	Adult	yes	F	ULM1	108	14	16	35	43	138.51	107.25	0.40	0.15
HUNG899	Bükkábrány-Bánya XI/B	MCA	20-40	Adult	yes	F	LRM2	151	5	28	56	62	114.61	73.67	0.41	0.19
HUNG901	Bükkábrány-Bánya XI/B	MCA	Adult	Adult	yes	NA	LLM1	107	16	9	25	57	126.27	67.16	0.53	0.08
HUNG903	Bükkábrány-Bánya XI/B	MCA	17-25	Juvenile-**Adult**	yes	F	ULM1	124	6	17	61	40	133.06	105.35	0.32	0.14
HUNG904	Bükkábrány-Bánya XI/B	MCA	45-49	Mature	yes	M	LRM2	105	14	9	51	31	166.89	122.08	0.30	0.09
HUNG908	Bükkábrány-Bánya XI/B	MCA	Adult	Adult	yes	NA	LRM1	156	11	4	47	94	141.87	76.21	0.60	0.03
HUNG909	Bükkábrány-Bánya XI/B	MCA	Adult	Adult	yes	NA	LRM1	111	13	7	22	69	151.11	94.87	0.62	0.06
HUNG910	Bükkábrány-Bánya XI/B	MCA	Adult	Adult	yes	NA	LRM2	135	25	32	45	33	140.62	97.59	0.24	0.24

HUNG911	Bükkábrány-Bánya XI/B	MCA	5-7	Infant I-**Infant II**	no	NA	LLM1	157	34	35	73	15	112.76	76.55	0.10	0.22
HUNG912	Bükkábrány-Bánya XI/B	MCA	Adult	Adult	yes	NA	ULM1	115	2	12	52	49	136.49	97.77	0.43	0.10
HUNG914	Bükkábrány-Bánya XII/A	LCA	9-12	Infant II	yes	NA	LRM1	90	2	13	48	27	119.30	94.52	0.30	0.14
HUNG915	Bükkábrány-Bánya XII/A	LCA	15-20	**Juvenile-**Adult	yes	F	LLM1	156	26	6	38	86	125.20	82.97	0.55	0.04
HUNG919	Bükkábrány-Bánya XII/B	LCA	35-45	Adult-Mature	yes	F	ULM1	111	2	5	44	60	138.91	91.57	0.54	0.05
HUNG130	Mezőzombor-Községi temető	MBA	20-39	Adult	yes	F	URM1	119	31	3	19	66	155.05	91.98	0.55	0.03
HUNG132	Mezőzombor-Községi temető	MBA	5-7	**Infant I-**Infant II	no	F	LRM1	122	18	4	26	74	123.91	62.67	0.61	0.03
HUNG134	Mezőzombor-Községi temető	MBA	5-10	Infant I-**Infant II**	no	M	LRM1	165	18	0	23	124	136.47	94.10	0.75	0.00
HUNG147	Mezőkeresztes -Csincsetanya	MBA	12-14	Infant II	yes	NA	ULM1	105	18	28	47	12	144.94	79.21	0.11	0.27
HUNG163	Nagyrozvágy-Papdomb	MBA	35-45	Adult-Mature	yes	M	URM2	124	33	3	10	78	142.06	95.83	0.63	0.02
HUNG933	Vatta-Dobogó	MBA	8-13	Infant II	yes	F	LLM1	135	50	11	20	54	120.52	65.27	0.40	0.08
HUNG934	Vatta-Dobogó	MBA	20-39	Adult	yes	NA	LLM1	97	16	24	31	26	149.64	113.52	0.27	0.25
HUNG937	Vatta-Dobogó	MBA	20-30	Adult	yes	NA	LRM1	112	9	28	30	45	152.87	112.98	0.40	0.25
HUNG177	Mezőkeresztes-Cethalom (M3-10. lelőhely)	LBA	6-10	Infant I-**Infant II**	no	NA	LRM1	115	19	5	14	77	212.46	148.81	0.67	0.04
HUNG863	Köröm-Kápolna-domb	LBA	20- 39	Adult	yes	F	ULM1	124	39	28	36	21	122.82	90.90	0.17	0.23
HUNG968	Pácin-Alsókenderszer	LBA	15-39	Juvenile-**Adult**	yes	M	ULM1	131	17	28	29	57	152.69	115.15	0.44	0.21

*The age category selected was chosen according to the higher number of years of the age range estimation belonging to each category. When age cohort is between Adult and Mature category, no choice was made as both categories were included together as adult in the statistical tests.

Table S2. Dataset by individual for occlusal surface. Abbreviations for tooth types correspond to jaw (upper or lower: U or L), side (left or right: L or R), and position in tooth row (first or second: 1 or 2) for each molar (M). Age in years; Age range category: Infant I (1-6 years), Infant II (7-14 years), Juvenile (15-19 years), Adult (20-39), and Mature (40-59)[1]; in bold the age category used for statistical analyses when the sample age (years) range include two age categories*. Adult diet[2]: see text for explanation. Gender (F= Female, M= Male, NA= No Data); SD: Standard deviation.

ID	Site	Period	Age	Age range category	Adult diet	Gender	Teeth	OTN	Pits	Area pits	SD Area	%PITS
HUNG871	Bükkábrány-Bánya VII	MN	7-8	Infant II	no	NA	LRM1	59	39	35.22	21.35	39.80
HUNG872	Bükkábrány-Bánya VII	MN	15-20	Juvenile	yes	M	LLM1	70	13	31.49	11.64	15.66
HUNG873	Bükkábrány-Bánya VII	MN	15-25	Juvenile - **Adult**	yes	NA	LRM1	54	14	55.67	23.95	20.59
HUNG877	Bükkábrány-Bánya VII	MN	20-30	Adult	yes	NA	LRM1	110	31	24.15	13.31	21.99
HUNG878	Bükkábrány-Bánya VII	MN	20-40	Adult	yes	NA	LRM1	96	21	31.39	17.00	17.95
HUNG880	Bükkábrány-Bánya X	MN	Adult	Adult	yes	NA	LRM2	44	21	24.96	16.64	32.31
HUNG884	Bükkábrány-Bánya XI/A	MN	30-40	Adult	yes	M	ULM1	55	13	52.23	50.71	19.12
HUNG885	Bükkábrány-Bánya XI/A	MN	35-45	Adult-Mature	yes	F	URM1	54	15	27.85	16.95	21.74
HUNG888	Bükkábrány-Bánya XI/A	MN	11-13	Infant II	yes	NA	LRM1	38	19	37.12	28.98	33.33
HUNG889	Bükkábrány-Bánya XI/A	MN	20-30	Adult	yes	M	LRM2	78	15	20.09	8.32	16.13
HUNG890	Bükkábrány-Bánya XI/A	MN	8-9	Infant II	no	F	URM1	61	30	17.62	12.34	32.97
HUNG893	Bükkábrány-Bánya XI/A	MN	11-12	Infant II	yes	M	LLM1	55	16	49.83	29.95	22.54
HUNG894	Bükkábrány-Bánya XI/A	MN	25-30	Adult	yes	F	LRM2	75	10	30.66	20.43	11.76
HUNG895	Bükkábrány-Bánya XI/A	MN	Adult	Adult	yes	M	LRM1	80	20	29.52	23.57	20.00
HUNG913	Bükkábrány-Bánya XII/A	MN	9-12	Infant II	yes	NA	LLM2	68	26	25.59	10.42	27.66
HUNG920	Bükkábrány-Bánya XII/B	MN	Adult	Adult	yes	NA	LRM1	40	19	24.79	18.36	32.20
HUNG924	Bükkábrány-Bánya XII/B	MN	Adult	Adult	yes	F	URM2	85	27	28.26	14.85	24.11
HUNG932	Bükkábrány-Bánya XII/B	MN	12-15	Juvenile	yes	NA	LLM2	NA	16	30.13	16.55	NA
HUNG896	Bükkábrány-Bánya XI/B	MCA	Adult	Adult	yes	NA	LRM2	52	10	28.04	11.35	16.13

HUNG897	Bükkábrány-Bánya XI/B	MCA	Adult	Adult	yes	M	LLM1	54	21	33.27	20.11	28.00
HUNG898	Bükkábrány-Bánya XI/B	MCA	Adult	Adult	yes	F	ULM1	46	13	NA	NA	22.03
HUNG899	Bükkábrány-Bánya XI/B	MCA	20-40	Adult	yes	F	LRM2	89	15	38.39	25.80	14.42
HUNG903	Bükkábrány-Bánya XI/B	MCA	17-25	Juvenile-**Adult**	yes	F	ULM1	106	8	35.15	13.08	7.02
HUNG904	Bükkábrány-Bánya XI/B	MCA	45-49	Mature	yes	F	LRM2	53	8	39.69	16.18	13.11
HUNG908	Bükkábrány-Bánya XI/B	MCA	Adult	Adult	yes	NA	LRM1	69	18	17.64	8.30	20.69
HUNG909	Bükkábrány-Bánya XI/B	MCA	Adult	Adult	yes	NA	LRM1	73	18	28.74	13.46	19.78
HUNG910	Bükkábrány-Bánya XI/B	MCA	Adult	Adult	yes	NA	LRM2	71	7	54.05	33.88	8.97
HUNG911	Bükkábrány-Bánya XI/B	MCA	5-7	**Infant I**-Infant II	no	NA	LLM1	45	23	30.77	13.21	33.82
HUNG912	Bükkábrány-Bánya XI/B	MCA	Adult	Adult	yes	NA	ULM1	47	9	35.39	23.38	16.07
HUNG914	Bükkábrány-Bánya XII/A	LCA	9-12	Infant II	yes	NA	LRM1	44	11	53.75	48.65	20.00
HUNG915	Bükkábrány-Bánya XII/A	LCA	15-20	**Juvenile**-Adult	yes	F	LLM1	63	16	41.11	27.05	20.25
HUNG917	Bükkábrány-Bánya XII/A	LCA	4-7	**Infant I**-Infant II	no	M	URM1	35	NA	NA	NA	NA
HUNG919	Bükkábrány-Bánya XII/B	LCA	35-45	Adult-Mature	yes	F	ULM1	47	7	68.93	49.02	12.96
HUNG127	Mezőzombor-Községi temető	MBA	20-39	Adult	yes	F	LLM2	32	9	38.16	28.68	21.95
HUNG132	Mezőzombor-Községi temető	MBA	5-7	**Infant I**-Infant II	no	F	LRM1	54	11	34.37	18.73	16.92
HUNG134	Mezőzombor-Községi temető	MBA	5-10	Infant I-**Infant II**	no	M	LRM1	NA	5	61.30	29.05	NA
HUNG147	Mezőkeresztes -Csincsetanya	MBA	12-14	Juvenile	yes	NA	ULM1	42	16	39.55	32.98	27.59
HUNG933	Vatta-Dobogó	MBA	8-13	Infant II	yes	F	LLM1	60	29	29.23	18.60	32.58
HUNG937	Vatta-Dobogó	MBA	20-30	Adult	yes	NA	LRM1	NA	12	47.28	30.00	NA
HUNG177	Mezőkeresztes-Cethalom (M3-10. lelőhely)	LBA	6-10	Infant I-**Infant II**	no	NA	LRM1	41	9	35.33	15.78	NA
HUNG968	Pácin-Alsókenderszer	LBA	15-39	Juvenile- **Adult**	yes	M	ULM1	81	23	25.33	16.53	22.12

*The age category selected was chosen according to the higher number of years of the age range estimation belonging to each category. When age cohort is between Adult and Mature category, no choice was made as both categories were included together as adult in the statistical tests.

Table S3. Mann-Whitney comparisons of buccal variables by period (N = 38). Lower diagonal: W-test values; upper diagonal: p-values. Significant values ($p<0.05$) in bold. Middle Neolithic (MN, n = 16); Middle Copper Age (MCA, n = 11); Late Copper Age (LCA, n = 3); Middle Bronze Age (MBA, n = 6); Late Bronze Age (LBA, n = 2).

Buccal microwear variables		MN	MCA	LCA	MBA	LBA
BTN	**MN**	-	**0.002**	0.240	**0.032**	**0.042**
	**MCA**	23.5	-	0.755	0.450	0.553
	**LCA**	13.0	14.0	-	0.897	0.773
	**MBA**	18.5	25.0	8.0	-	0.314
	**LBA**	1.0	7.5	2.0	2.5	-
XT	**MN**	-	**0.004**	0.240	**0.004**	0.232
	**MCA**	29.0	-	0.350	0.291	0.278
	**LCA**	13.0	10.0	-	0.093	0.773
	**MBA**	8.0	22.0	2.0	-	0.243
	**LBA**	5.0	11.0	2.0	5.0	-

Table S4. Mann-Whitney comparisons of buccal variables by Middle Neolithic sites (N = 14). Lower diagonal: W-test values; upper diagonal: p- values. Significant values ($p<0.05$) in bold. Sites: Bükkábrány-Bánya VII (BB-VII, n = 4); Bükkábrány-Bánya XI/A (BB-XI/A, n = 7); Bükkábrány-Bánya XII/B (BB-XII/B, n = 3).

Buccal microwear variables		BB-VII	BB-XI/A	BB-XII/B
BTN	**BB-VII**	-	**0.047**	0.052
	**BB-XI/A**	3.0	-	0.424
	**BB-XII/B**	0.0	6.5	-
NV/TN	**BB-VII**	-	0.705	0.052
	**BB-XI/A**	11.5	-	**0.023**
	**BB-XII/B**	0.0	0.0	-
NH/TN	**BB-VII**	-	0.155	0.108
	**BB-XI/A**	6.0	-	**0.040**
	**BB-XII/B**	1.0	1.0	-

Table S5. Mann-Whitney comparison of buccal variables between males (M) and females (F). N= Total number of individuals. For graphical representation by period see Figures S3 and S4.

**Gender**		**W-test**	**p-value**
All samples (N=20)			
(M=9 F=11)	**BTN**	63.5	0.304
	**XT**	35.0	0.294
	**NV/TN**	25.0	0.067
	**NH/TN**	66.5	0.208
By period			
Middle Neolithic (N=8)	**BTN**	5.0	0.571
(M=5, F=3)	**XT**	2.0	0.142
	**NV/TN**	5.0	0.571
	**NH/TN**	10.0	0.571
Middle Copper Age (N=5)	**BTN**	4.0	0.800
(M=2, F=3)	**XT**	0.0	0.200
	**NV/TN**	3.0	1.000
	**NH/TN**	6.0	0.200

Table S6. Kruskal-Wallis test performed in occlusal variables by period (N=34). For graphical representation see Figure S5.

**Occlusal variable**	X^2	df	p-value
OTN	5.85	4	0.210
PITS	8.69	4	0.068
AREA PITS	8.75	4	0.068
% PITS	8.61	4	0.071

Table S7. Kruskal-Wallis test performed in occlusal variables by Middle Neolithic sites (N=14). For graphical representation see Figure S6.

**Occlusal variable**	X^2	df	p-value
OTN	1.36	2	0.505
PITS	1.98	2	0.369
AREA PITS	1.48	2	0.477
% PITS	3.35	2	0.187

Table S8. Mann-Whitney comparison of occlusal variables between males (M) and females (F). N= Total number of individuals. For graphical representation by period see Figure S7.

		W test	p-value
**All samples** (N=18)	**OTN**	31.5	0.555
(M=7, F=11)	**PITS**	23.5	0.186
	**AREA PITS**	29.0	0.591
	**% PITS**	29.0	0.415
**By period**			
Middle Neolithic (N=9)	**OTN**	7.0	1.000
(M=5, F=3)	**PITS**	7.5	0.880
	**AREA PITS**	4.0	0.371
	**% PITS**	6.0	0.765

1. Martin, R. & Saller, K. *Lehrbuch der Anthropologie, in systematischer Darstellung*. (Gustav Fischer Verlag, 1957).

2. Hernando, R. *et al.* Inferring childhood dietary maturation using buccal and occlusal deciduous molar microwear: a case study from the recent prehistory of the Iberian Peninsula. *Archaeol. Anthropol. Sci.* **12**, 30 (2020).
