# Supplementary material for: Integrating buccal and occlusal dental microwear with isotope analyses for a complete paleodietary reconstruction of Holocene populations from Hungary
Source: Sci Rep. 2021 Mar 29;11:7034. doi: 10.1038/s41598-021-86369-x (PMC8007593; doi:10.1038/s41598-021-86369-x)
Supplement: Supplementary file 7 — Supplementary Information 7. [file 41598_2021_86369_MOESM7_ESM.pdf]

Supplementary Results: Statistical analyses of isotope results. Tables S9-S14.

Integrating buccal and occlusal dental microwear with isotope analyses for a complete paleodietary reconstruction of Holocene populations from Hungary.

Raquel Hernando[1,2*], Beatriz Gamarra[2,1,3*], Ashley McCall[3], Olivia Cheronet[4,3], Daniel Fernandes[4,5,3], Kendra Sirak[6,7,3], Ryan Schmidt[8,3], Marina Lozano[2,1], Tamás Szeniczey[9,10], Tamás Hajdu[9,10], Annamária Bárány[11], András Kalli[12], Eszter K. Tutkovics[13], Kitti Köhler[14], Krisztián Kiss[9,10], Judit Koós[15], Piroska Csengeri[15], Ágnes Király[14], Antónia Horváth[15], Melinda L. Hajdú[15], Krisztián Tóth[16], Róbert Patay[17], Robin N. M. Feeney[18], Ron Pinhasi[4]

*Corresponding authors: r.hernando90@gmail.com and beagamarra@gmail.com. These authors contributed equally to this work.

[1]Universitat Rovira i Virgili, Departament d'Història i Història de l'Art, Avinguda de Catalunya 35, 43002 Tarragona, Spain.
[2]Institut Català de Paleoecologia Humana i Evolució Social (IPHES), Zona Educacional 4, Campus Sescelades URV (Edifici W3), 43007 Tarragona, Spain.
[3]School of Archaeology and Earth Institute, University College Dublin, Dublin, Ireland.
[4]Department of Evolutionary Anthropology, University of Vienna, Vienna, Austria.
[5]CIAS, Department of Life Sciences, University of Coimbra, 3000-456 Coimbra, Portugal.
[6]Department of Genetics, Harvard Medical School, Boston, MA 02115, USA.
[7]Department of Human Evolutionary Biology, Harvard University, Cambridge, MA 02138, USA
[8]CIBIO-InBIO, Universidade do Porto, Portugal.
[9]Department of Biological Anthropology, Eötvös Loránd University, Budapest, H-1117 Pázmány Péter sétány 1/c.
[10]Department of Anthropology, Hungarian Natural History Museum, Budapest, H-1083, Ludovika tér 2.
[11]Department of Archaeology, Hungarian National Museum, Budapest, H-1088, Múzeum krt. 14-16.
[12]Várkapitányság Integrált Területfejlesztési Központ Nonprofit Zrt., H-1113 Budapest, Daróczi út 3., Hungary.
[13]Rétközi Museum, H-4600 Kisvárda, Csillag u. 5., Hungary.
[14]Institute of Archaeology, Research Centre for the Humanities, Loránd Eötvös Research Network, Budapest, H-1097 Tóth Kálmán utca 4.
[15]Herman Ottó Museum, H- 3529 Miskolc, Görgey Artúr u. 28, Hungary.
[16]Dornyay Béla Museum, H-3100 Salgótarján, Múzeum tér 2., Hungary.
[17]Department of Archaeology, Ferenczy Museum Center, Szentendre, H-2000 Fő tér 2–5.
[18]School of Medicine, University College Dublin, Dublin, Ireland.

Abbreviations: Middle Neolithic (MN); Late Neolithic (LN); Middle Copper Age (MCA); Late Copper Age (LCA); Middle Bronze Age (MBA); Late Bronze Age (LBA).

MN site abbreviations: Bükkábrány-Bánya VII (BB-VII); Bükkábrány-Bánya XI/A (BB-XI/A); Bükkábrány-Bánya XII/B (BB-XII/B).

Table S9. Isotope results of the faunal samples included in this study. Details of each sample are included.

ID	Species	Site	Period	Inventory num	Bone element	δ¹⁵N AIR‰	δ¹³C PDB‰	N%	C%	C/N
HUNGF_34	cattle	Bükkábrány Bánya site VII	MN	325	rib	8.3	-19.9	14.8	41.4	3.3
HUNGF_39	sheep/goat	Bükkábrány Bánya site VII	MN	208	rib	7.4	-20.6	16.3	45.0	3.2
HUNGF_40	pig	Bükkábrány Bánya site VII	MN	47	scapula	10.5	-21.0	14.8	43.2	3.4
HUNGF_41	aurochs	Bükkábrány Bánya site VII	MN	90	humerus					**no collagen**
HUNGF_42	cattle	Bükkábrány Bánya site VII	MN	208	rib	7.2	-19.7	15.9	43.8	3.2
HUNGF_44	pig	Bükkábrány Bánya site VII	MN	208	rib	9.0	-20.7	15.6	43.4	3.2
HUNGF_19	sheep/goat	Mezőkövesd-Patakra járó dűlő	MCA	grave 4/a	humerus	7.7	-21.3	13.5	38.4	3.3
HUNGF_20	sheep/goat	Mezőkövesd-Patakra járó dűlő	MCA	grave 6.	rib	7.6	-20.6	15.1	43.9	3.4
HUNGF_36	sheep/goat	Bükkábrány Bánya site XII/B	LCA	89	rib	7.6	-19.7	15.4	42.6	3.2
HUNGF_46	wild pig/boar	Bükkábrány Bánya site XII/A	LCA	888	metatarsus	6.4	-20.2	15.8	43.9	3.3
HUNGF_48	hare	Bükkábrány Bánya site XII/A	LCA	882	pelvis	6.1	-21.7	15.6	42.8	3.2
HUNGF_49	cattle	Bükkábrány Bánya site XII/A	LCA	938	rib	7.1	-21.1	15.8	45.8	3.4
HUNGF_50	dog	Bükkábrány Bánya site XII/A	LCA	1103	mandible	9.3	-19.8	7.1	20.1	3.3
HUNGF_51	hare/rabbit	Bükkábrány Bánya site XII/A	LCA	881	radius	5.9	-21.9	15.2	42.0	3.2
HUNGF_52	pig	Bükkábrány Bánya site XII/A	LCA	824	rib	9.1	-20.6	15.7	43.3	3.2
HUNGF_53	sheep/goat	Bükkábrány Bánya site XII/A	LCA	900	rib	9.0	-19.5	15.6	43.7	3.3
HUNGF_1	cattle	Vatta-Dobogó	MBA	339	rib	8.2	-20.3	15.0	42.1	3.3
HUNGF_2	dog	Vatta-Dobogó	MBA	290/1.	metapodium	6.1	-20.8	15.5	43.7	3.3
HUNGF_3	cattle	Vatta-Dobogó	MBA	290/1.	rib	6.3	-20.8	15.9	44.1	3.2
HUNGF_4	pig	Vatta-Dobogó	MBA	290/1.	rib	8.1	-20.4	15.7	44.3	3.3
HUNGF_5	pig	Vatta-Dobogó	MBA	290/II.	rib	8.6	-20.3	15.5	43.3	3.3
HUNGF_8	fish	Vatta-Dobogó	MBA	290/II.	vertebra	11.7	-22.9	16.1	43.2	3.1
HUNGF_10	hare	Vatta-Dobogó	MBA	290/II.	humerus	5.5	-22.6	15.6	43.9	3.3
HUNGF_27	pig	Pácin-Alsókenderszer	LBA	157	humerus	7.8	-21.8	15.5	43.8	3.3
HUNGF_28	dog	Pácin-Alsókenderszer	LBA	106	metatarsus	9.2	-17.9	15.8	43.3	3.2
HUNGF_29	sheep/goat	Pácin-Alsókenderszer	LBA	106	rib	7.4	-20.3	15.6	42.8	3.2
HUNGF_30	horse	Pácin-Alsókenderszer	LBA	59	metapodium					**no collagen**
HUNGF_31	cattle	Pácin-Alsókenderszer	LBA	50	rib	8.4	-19.9	15.6	42.7	3.2
HUNGF_32	cat	Pácin-Alsókenderszer	LBA	50	ulna	11.0	-18.8	15.9	43.7	3.2

Table S10. Isotope results of the human samples included in this study. Details of each samples are included. Age range category: Infant I (1-6 years), Infant II (7-14 years), Juvenile (15-19 years), Adult (20-39), and Mature (40-59)[1]; in bold the age category used for statistical analyses when the sample age (years) range include two age categories*. Age at death abbreviations: not possible to assign (?). Sex abbreviations: female (F), male (M).

ID	**Site**	**Period**	**Years**[b]	**Age Range Category**	**Sex**[c]	**Bone element**	**δ¹⁵N AIR‰**	**δ¹³C PDB‰**	**N%**	**C%**	**C/N**
HUNG870	Bükkábrány-Bánya VII	MN	30-34	Adult		long bone	10.8	-20.5	14.8	41.4	3.2
HUNG872	Bükkábrány-Bánya VII	MN	15-20	Juvenile	M	long bone	11.0	-20.3	15.3	42.8	3.3
HUNG873	Bükkábrány-Bánya VII	MN	15-25	Juvenile-**Adult**		cranial fragment	10.6	-20.3	15.1	43.2	3.3
HUNG874	Bükkábrány-Bánya VII	MN	8-9	Infant II	M	temporal fragment	9.9	-20.5	14.8	42.6	3.3
HUNG876	Bükkábrány-Bánya VII	MN	15-20	Juvenile-**Adult**		cranial fragment	11.0	-19.9	13.7	38.3	3.4
HUNG877	Bükkábrány-Bánya VII	MN	20-30	Adult		tibia	10.7	-20.3	14.0	41.2	3.4
HUNG878	Bükkábrány-Bánya VII	MN	20-40	Adult		femur	10.6	-20.5	14.4	42.4	3.3
HUNG879	Bükkábrány-Bánya VII	MN	3-5	Infant I		rib	10.2	-20.5	15.3	44.0	3.2
HUNG880	Bükkábrány-Bánya X	MN	Adult	Adult		long bone	11.6	-20.9	8.3	25.0	3.3
HUNG882	Bükkábrány-Bánya XI/A	MN	1-6	Infant I		long bone	10.5	-20.9	13.5	41.3	3.4
HUNG883	Bükkábrány-Bánya XI/A	MN	30-50	Adult-Mature	M	long bone	10.8	-20.3	13.5	37.8	3.2
HUNG884	Bükkábrány-Bánya XI/A	MN	30-40	Adult	M	petrous fragment	10.5	-20.4	14.5	40.7	3.3
HUNG885	Bükkábrány-Bánya XI/A	MN	35-45	Adult-Mature	F	cranial fragment	10.8	-20.4	10.8	31.0	3.2
HUNG887	Bükkábrány-Bánya XI/A	MN	35-45	Adult-Mature	M	rib	11.0	-20.1	14.8	41.5	3.2
HUNG888	Bükkábrány-Bánya XI/A	MN	11-13	Infant II		long bone	10.7	-20.2	14.1	40.6	3.3
HUNG889	Bükkábrány-Bánya XI/A	MN	20-30	Adult	M	long bone	9.9	-20.2	13.7	39.0	3.3
HUNG890	Bükkábrány-Bánya XI/A	MN	8-9	Infant II	F	temporal fragment	9.5	-20.4	14.3	41.6	3.3

HUNG891	Bükkábrány-Bánya XI/A	MN	1-6	Infant I	F	petrous fragment	12.2	-20.1	14.6	41.5	3.3
HUNG892	Bükkábrány-Bánya XI/A	MN	1-3	Infant I	F	petrous fragment	13.2	-19.6	15.1	42.1	3.3
HUNG893	Bükkábrány-Bánya XI/A	MN	11-12	Infant II	M	petrous fragment	11.5	-20.2	15.3	42.7	3.2
HUNG894	Bükkábrány-Bánya XI/A	MN	25-30	Adult	F	petrous fragment	11.8	-20.1	15.1	43.2	3.3
HUNG895	Bükkábrány-Bánya XI/A	MN	Adult	Adult	M	long bone	10.9	-20.4	9.9	28.2	3.3
HUNG913	Bükkábrány-Bánya XII/A	MN	9-12	Infant II		long bone	11.4	-20.5	14.5	41.3	3.3
HUNG920	Bükkábrány-Bánya XII/B	MN	Adult	Adult		cranial fragment	11.1	-20.1	14.3	41.6	3.3
HUNG921	Bükkábrány-Bánya XII/B	MN	Adult	Adult	M	cranial fragment	11.2	-20.3	15.1	41.9	3.3
HUNG922	Bükkábrány-Bánya XII/B	MN	Adult	Adult		cranial fragment	10.9	-19.8	14.3	39.9	3.4
HUNG923	Bükkábrány-Bánya XII/B	MN	20-30	Adult		cranial fragment	12.4	-20.0	13.1	37.0	3.3
HUNG924	Bükkábrány-Bánya XII/B	MN	Adult	Adult	F	long bone	10.9	-20.1	14.5	41.2	3.4
HUNG926	Bükkábrány-Bánya XII/B	MN	Adult	Adult	M	rib	12.5	-19.7	15.2	43.5	3.4
HUNG927	Bükkábrány-Bánya XII/B	MN	Adult	Adult		long bone	**10.6**	**-22.8**	**2.8**	**11.4**	**4.8**
HUNG928	Bükkábrány-Bánya XII/B	MN	1-6	Infant I	F	petrous fragment	11.5	-19.6	14.2	40.2	3.4
HUNG929	Bükkábrány-Bánya XII/B	MN	Adult	Adult		long bone	11.7	-20.2	6.1	18.3	3.5
HUNG930	Bükkábrány-Bánya XII/B	MN	Adult	Adult		cranial fragment	11.4	-20.8	11.3	33.8	3.6
HUNG931	Bükkábrány-Bánya XII/B	MN	1-6	Infant I	F	petrous fragment	12.1	-20.2	14.0	39.0	3.3
HUNG932	Bükkábrány-Bánya XII/B	MN	12-15	**Infant II-** Juvenile		cranial fragment	10.9	-20.3	15.0	43.2	3.3
HUNG941	Rásonysápberencs-Szőlő alja	MN	Adult	Adult	M	petrous fragment	10.2	-19.9	14.7	40.7	3.3
HUNG942	Rásonysápberencs-Szőlő alja	MN	Adult	Adult	F	petrous fragment	9.9	-19.9	14.8	40.8	3.3

HUNG943	Rásonysápberencs-Szőlő alja	MN	9-12	Infant II	F	rib	9.6	-20.3	14.9	41.5	3.4
HUNG948	Csincse-Gomba Barna földje (M3-site 14-16	MN	Adult	Adult	F	petrous fragment	**10.2**	**-21.6**	**5.7**	**18.6**	**3.8**
HUNG953	Arnót-Nagy-bugyik	MN	20-35	Adult		petrous fragment	10.4	-20.8	15.5	43.2	3.4
HUNG955	Arnót-Nagy-bugyik	MN	2-5	Infant I	M	petrous fragment	12.4	-19.9	14.8	42.6	3.3
HUNG956	Arnót-Arnóti-oldal Dél	MN	30-50	Adult-Mature	F	rib	10.1	-20.0	12.4	34.7	3.3
HUNG896	Bükkábrány-Bánya XI/B	MCA	Adult	Adult		long bone	6.9	-20.9	12.4	35.5	3.3
HUNG897	Bükkábrány-Bánya XI/B	MCA	Adult	Adult	M	petrous fragment	11.5	-19.7	14.9	41.8	3.3
HUNG898	Bükkábrány-Bánya XI/B	MCA	Adult	Adult	F	long bone	10.6	-19.6	15.1	42.2	3.3
HUNG899	Bükkábrány-Bánya XI/B	MCA	20-40	Adult	F	petrous fragment	10.4	-20.3	14.5	42.4	3.3
HUNG900	Bükkábrány-Bánya XI/B	MCA	Adult	Adult		long bone	10.1	-20.7	3.9	11.7	3.4
HUNG901	Bükkábrány-Bánya XI/B	MCA	Adult	Adult		long bone	10.6	-19.8	12.1	34.3	3.2
HUNG902	Bükkábrány-Bánya XI/B	MCA	Adult	Adult		long bone	11.1	-20.1	15.1	42.6	3.3
HUNG903	Bükkábrány-Bánya XI/B	MCA	17-25	Adult	F	long bone	9.8	-19.7	13.9	38.6	3.3
HUNG904	Bükkábrány-Bánya XI/B	MCA	45-49	Mature	M	cranial fragment	10.6	-19.9	13.5	37.6	3.3
HUNG908	Bükkábrány-Bánya XI/B	MCA	Adult	Adult		parietal fragment	10.6	-20.0	13.1	37.2	3.3
HUNG909	Bükkábrány-Bánya XI/B	MCA	Adult	Adult		cranial fragment	9.8	-20.4	14.2	41.2	3.5
HUNG910	Bükkábrány-Bánya XI/B	MCA	Adult	Adult		long bone	10.2	-20.6	15.2	42.7	3.5
HUNG911	Bükkábrány-Bánya XI/B	MCA	5-7	Infant I		long bone	10.1	-20.2	14.1	40.8	3.2
HUNG939	Mezőkövesd-Klementina (Szentistván-Reptér)	MCA	12-18	Infant II-**Juvenile**	F	rib	10.3	-19.9	15.7	43.3	3.3
HUNG940	Mezőkövesd-Klementina (Szentistván-Reptér)	MCA	Adult	Adult	M	temporal fragment	10.4	-19.9	15.2	42.9	3.2

HUNG961	Mezőkövesd-Patakra járó dűlő	MCA	17-21	**Juvenile-** Adult	M	long bone	10.1	-19.5	15.6	43.0	3.2
HUNG962	Mezőkövesd-Patakra járó dűlő	MCA	5-6	Infant I	M	petrous fragment	10.2	-19.7	15.3	42.7	3.2
HUNG963	Mezőkövesd-Patakra járó dűlő	MCA	1-6	Infant I	M	petrous fragment	10.3	-20.2	13.2	37.4	3.2
HUNG964	Mezőkövesd-Patakra járó dűlő	MCA	10-12	Infant II		ulna	10.2	-20.1	15.0	41.7	3.2
HUNG965	Mezőkövesd-Patakra járó dűlő	MCA	40-60	Mature	M	metacarpal	10.8	-19.9	10.4	30.0	3.4
HUNG966	Mezőkövesd-Patakra járó dűlő	MCA	30-60	Adult-Mature	F	long bone	10.1	-20.1	14.7	42.2	3.3
HUNG915	Bükkábrány-Bánya XII/A	LCA	15-20	**Juvenile-** Adult	F	petrous fragment	12.3	-20.7	15.2	42.3	3.3
HUNG917	Bükkábrány-Bánya XII/A	LCA	4-7	**Infant I –** Infant II	M	cranial fragment	10.6	-20.6	12.3	36.8	3.5
HUNG918	Bükkábrány-Bánya XII/B	LCA	39-50	Adult-Mature	F	cranial fragment	12.0	-19.9	14.6	41.4	3.3
HUNG919	Bükkábrány-Bánya XII/B	LCA	35-45	Adult-Mature	F	petrous fragment	11.7	-20.2	15.4	43.3	3.3
HUNG127 [a]	Mezőzombor-Községi temető	MBA	20-39	Adult	F	metatarsal	11.5	-20.2	15.4	43.4	3.3
HUNG128 [a]	Mezőzombor-Községi temető	MBA	8-13	Infant II	M	metatarsal	10.7	-19.9	15.2	42.5	3.3
HUNG129 [a]	Mezőzombor-Községi temető	MBA	35-44	Adult-Mature	M	metacarpal	11.3	-19.5	15.3	43.2	3.3
HUNG130 [a]	Mezőzombor-Községi temető	MBA	20-39	Adult	F	metatarsal	11.2	-20.1	15.6	43.0	3.2
HUNG131 [a]	Mezőzombor-Községi temető	MBA	34-42	Adult-Mature	M	metacarpal	12.2	-20.6	15.0	42.4	3.3
HUNG132 [a]	Mezőzombor-Községi temető	MBA	5-7	**Infant I-** InfantII	F	metatarsal	8.9	-20.2	15.4	43.2	3.3
HUNG133 [a]	Mezőzombor-Községi temető	MBA	20-39	Adult	M	metatarsal	11.2	-20.1	15.0	42.1	3.3
HUNG134 [a]	Mezőzombor-Községi temető	MBA	5-10	Infant I- **Infant II**	M	metatarsal	11.7	-20.1	15.3	42.4	3.2

HUNG135 [a]	Mezőzombor-Községi temető	MBA	35-50	Adult-Mature	M	metatarsal	10.9	-19.9	15.2	42.5	3.2
HUNG136 [a]	Mezőzombor-Községi temető	MBA	33-46	Adult-Mature	F	metatarsal	11.3	-19.9	15.6	43.5	3.3
HUNG147 [a]	Mezőkeresztes - Csincsetanya	MBA	12-14	Infant II		metacarpal	10.6	-16,7	15.4	42.9	3.3
HUNG163 [a]	Nagyrozvágy-Papdomb	MBA	35-45	Adult-Mature	M	metacarpal	11.1	-21.2	14.1	42.8	3.5
HUNG933 [a]	Vatta-Dobogó	MBA	8-13	Infant II	F	cranial fragment	9.5	-17.2	14.5	41.5	3.3
HUNG934 [a]	Vatta-Dobogó	MBA	20-39	Adult		femur	10.3	-17.4	11.2	31.7	3.3
HUNG935 [a]	Vatta-Dobogó	MBA	Adult	Adult	F	long bone	9.5	-17.8	15.9	44.7	3.3
HUNG936 [a]	Vatta-Dobogó	MBA	40-59	Mature	M	long bone	11.2	-21.0	6.8	20.6	3.5
HUNG937	Vatta-Dobogó	MBA	20-30	Adult		long bone	**11.1**	**-18.7**	**12.0**	**40.2**	**3.9**
HUNG938 [a]	Vatta-Dobogó	MBA	20-39	Adult	F	rib	9.4	-18.1	15.8	44.5	3.3
HUNG137 [a]	Felsődobsza-2. lelőhely	LBA	34-42	Adult-Mature	M	metatarsal	10.8	-15.1	15.4	42.8	3.3
HUNG144 [a]	Oszlár-Nyárfaszög (M3-32. lelőhely)	LBA	20-39	Adult	M	metacarpal	10.9	-16.8	15.6	43.9	3.3
HUNG177 [a]	Mezőkeresztes-Cethalom (M3-10. lelőhely)	LBA	6-10	Infant I-**Infant II**		metacarpal	10.2	-17.4	15.1	42.3	3.3
HUNG863 [a]	Köröm-Kápolna-domb	LBA	20- 39	Adult	F	cranial fragment	9.9	-17.9	14.0	39.1	3.3
HUNG967 [a]	Pácin-Alsókenderszer	LBA	1-6	Infant I	M	rib	12.97	-14.85	15.1	42.0	3.2
HUNG968 [a]	Pácin-Alsókenderszer	LBA	15-39	Juvenile-**Adult**	M	cranial fragment	9.8	-18.1	14.3	40.1	3.3
HUNG969 [a]	Pácin-Alsókenderszer	LBA	30-60	Adult-Mature	M	cranial fragment	11.6	-17.4	9.0	25.6	3.3

*The age category selected was chosen according to the higher number of years of the age range estimation belonging to each category. When age cohort is between Adult and Mature category, no choice was made as both categories were included together as adult in the statistical tests.

[a] Data from[1].
[b] Approximations of years' range were given when possible.
[c] Sex-assigned according to DNA and osteological information. See main text for explanation.

Table S11. Mann-Whitney comparisons of $\delta^{13}C$ values by period (N = 74). Lower diagonal: W-test values; upper diagonal: p-values. Significant values ($p<0.05$) in bold. Middle Neolithic (MN, n = 33); Middle Copper Age (MCA, n = 17); Late Copper Age (LCA, n = 3); Middle Bronze Age (MBA, n = 15); Late Bronze Age (LBA, n = 6).

	MN	MCA	LCA	MBA	LBA
**MN**	-	**0.008**	0.885	**0.014**	**<0.001**
**MCA**	152.0	-	0.262	0.447	**<0.001**
**LCA**	46.5	14.5	-	0.283	**0.028**
**MBA**	137.5	107.0	13.0	-	**0.019**
**LBA**	0.0	0.0	0.0	14.5	-

Table S12. Mann-Whitney comparisons of $\delta^{15}N$ values by period (N = 74). Lower diagonal: W-test values; upper diagonal: p-values. Significant values ($p<0.05$) in bold. Middle Neolithic (MN, n = 33); Middle Copper Age (MCA, n = 17); Late Copper Age (LCA, n = 3); Middle Bronze Age (MBA, n = 15); Late Bronze Age (LBA, n = 6).

	MN	MCA	LCA	MBA	LBA
**MN**	-	**0.015**	**0.017**	0.903	0.320
**MCA**	161.0	-	**0.008**	0.061	0.778
**LCA**	7.5	0.0	-	**0.017**	**0.028**
**MBA**	241.5	77.5	2.0	-	0.483
**LBA**	73.0	46.5	0.0	35.5	-

Table S13. Mann-Whitney comparisons of δ¹⁵N values in Middle Neolithic sites (N= 26). Lower diagonal: W-test values; upper diagonal: p-values. Significant values ($p<0.05$) in bold. Sites: Bükkábrány-Bánya VII (BB-VII, n = 7); Bükkábrány-Bánya XI/A (BB-XI/A, n = 10); Bükkábrány-Bánya XII/B (BB-XII/B, n = 9).

	**BB-VII**	**BB-XI/A**	**BB-XII/B**
**BB-VII**	-	0.731	**0.008**
**BB-XI/A**	31.0	-	**0.027**
**BB-XII/B**	6.0	17.5	-

Table S14. Mann-Whitney comparisons of $\delta^{13}C$ and $\delta^{15}N$ values between males (M) and females (F). N= Number of individuals. For graphical representation by period see Figures S12.

		W-test	p-value
**All samples (N=49)**	$\delta^{13}C$	284.5	0.808
(M=27, F=22)	$\delta^{15}N$	218.5	0.116
**By period**			
Middle Neolithic (N=18)			
(M=11, F=7)	$\delta^{13}C$	44.0	0.646
	$\delta^{15}N$	23.0	0.172
Middle Copper Age			
(N=10); (M=5, F=5)	$\delta^{13}C$	9.0	0.516
	$\delta^{15}N$	5.5	0.170
Middle Bronze Age			
(N=13); (M=7, F=6)	$\delta^{13}C$	9.5	0.113
	$\delta^{15}N$	15.5	0.471

1. Martin, R. & Saller, K. *Lehrbuch der Anthropologie, in systematischer Darstellung*. (Gustav Fischer Verlag, 1957).
